# Hydrazone Activation in the Aminocatalytic Cascade Reaction for the Synthesis of Tetrahydroindolizines

**DOI:** 10.1021/acs.orglett.3c03911

**Published:** 2024-01-24

**Authors:** Justyna Kowalska, Beata Łukasik, Sebastian Frankowski, Łukasz Albrecht

**Affiliations:** † Institute of Organic Chemistry, Lodz University of Technology, Żeromskiego 116, 90-924 Łódź, Poland

## Abstract

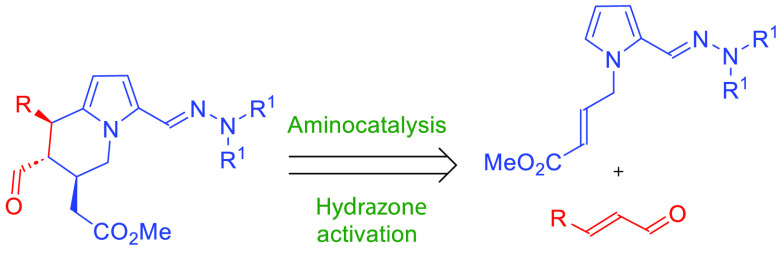

In this Letter, we demonstrate the usefulness of hydrazone activation for the synthesis of biologically relevant tetrahydroindolizines. A pyrrol-derived hydrazone bearing a Michael acceptor moiety in the *N*-alkyl side chain has been designed with the aim of participating in the aminocatalytic cascade reaction leading to the annulation of the new six-membered heterocyclic scaffold. The application of (*S*)-(−)-α,α-diphenyl-2-pyrrolidinemethanol trimethylsilyl ether as the aminocatalyst allows for the iminium ion–enamine-mediated cascade to proceed in a fully stereoselective manner.

Nitrogen heterocycles constitute privileged structures present in many natural products and pharmacologically active molecules.^1^ Among them, compounds containing a substituted pyrrole ring are of particular importance to medicinal and synthetic chemists.^2^ Tetrahydroindolizine and its derivatives are a unique group of pyrrole-ring-containing heterocycles possessing an additional tetrahydropyridine with interesting biological properties. Representative examples of tetrahydroindolizines relevant for the life-science industry are shown in the Figure 1.^3−6^

**Figure 1:**
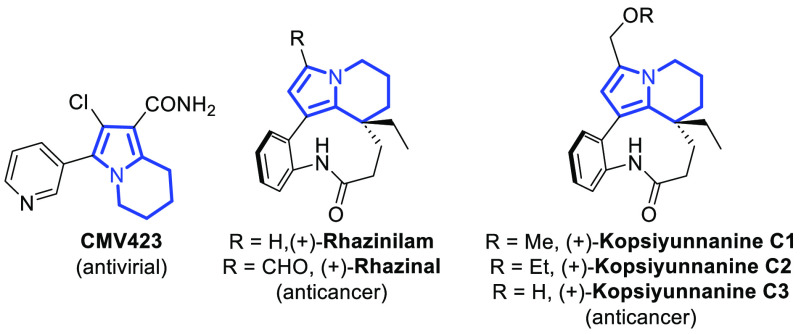
Representative examples of biologically relevant tetrahydroindolizine derivatives.

In classical organic synthesis, polar reactions between an electron-rich nucleophile and an electron-poor electrophile constitute a common approach. Already in the 1960s, Corey and Seebach introduced a concept of umpolung, allowing new bond-forming processes to be obtained following the principle of polarity inversion of functional groups.^7^ One way to access umpolung of the carbonyl group relies on the application of the hydrazone activation phenomenon (Scheme 1, top).^8,9^ It should be noted that *N*,*N*-dialkyl hydrazones of aldehydes are isoelectronic with the corresponding enamines and therefore are considered as aza-enamines (Scheme 1, middle).^10^ Moreover, the nucleophilicity enhancement provided by the hydrazone moiety can be effectively transferred over the conjugated double bond system,^11,12^ with *N*,*N*-dialkyl hydrazones derived from heteroaromatic aldehydes being particularly useful group of vinylogous reactants (Scheme 1, bottom).^13^

**Scheme 1:**
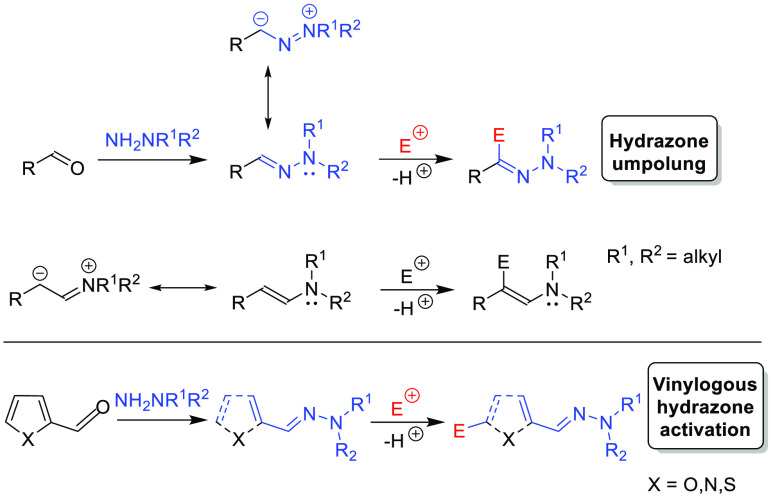
Principles of Umpolung and the Vinylogous Hydrazone Strategy in Asymmetric Synthesis

Recently, we demonstrated that hydrazone activation of (hetero)aromatic aldehydes constitutes a useful tool for the development of new organocatalytic transformations.^14−16^ It was shown that such a strategy can be efficiently employed in the asymmetric Friedel–Crafts reaction, (3 + 2)-cycloaddition (for the synthesis of 2,3-dihydro-1*H*-pyrrolyzines),^15^ or asymmetric [4 + 2]-cycloaddition with hydrazone derived from 9-carboxyanthracene, leading to the formation of the biologically relevant dihydro-9,10-ethaneanthracene scaffold.^16^

Given our interest in the hydrazone activation of (hetero)aromatic aldehydes and the particular usefulness of pyrrole derivatives for the development of new reaction pathways, we turned our attention to tetrahydroindolizine derivatives. It was anticipated that the introduction of a Michael acceptor moiety in the *N*-alkyl side chain present at the nitrogen atom of pyrrole-derived hydrazone would lead to an interesting reactant. It should be able to participate in a reaction cascade with the appropriately selected Michael acceptor, involving a Friedel–Crafts reaction followed by annulation of the new six-membered heterocyclic ring via an intramolecular Michael reaction.

Herein, we present our studies on the application of asymmetric aminocatalysis in the synthesis of highly functionalized tetrahydroindolizines. The reaction of hydrazone derivative **1** bearing a Michael acceptor moiety in the side-chain with α,β-unsaturated aldehydes **2** realized under aminocatalytic conditions^17^ proceeded in a cascade manner and resulted in the annulation of the new heterocyclic scaffold (Scheme 2).

**Scheme 2:**
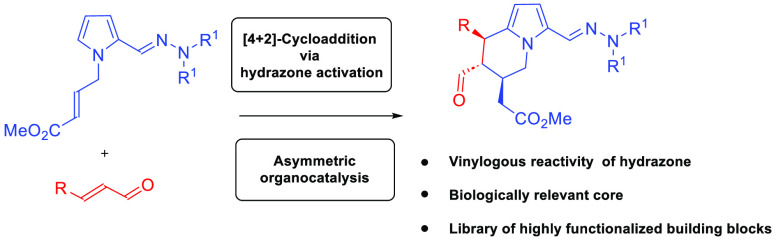
Presentation of Our Synthetic Goals

Optimization studies were performed using hydrazone **1a** and cinnamaldehyde **2a** as model substrates (Table 1). Initially, catalyst screening was performed (Table 1, entries 1–7) in dichloromethane at room temperature in the presence of trifluoroacetic acid as the cocatalyst (with the exception of the catalyst **3f** that was employed as hydrochloride). The course of the reaction was controlled by analyzing ^1^H NMR spectra of the crude reaction mixture after 24 h. For catalysts **3a**, **3b**, **3f**, and **3g**, only Friedel–Crafts alkylation took place, and no formation of the desired **4a** was observed (Table 1, entries 1, 2, 6, and 7, respectively). The remainder of the tested prolinol derivatives **3c**–**e** allowed us to obtain a mixture of compound **5a** and its cyclization product **4a** (Table 1, entries 3–5, respectively). Encouraged by the excellent diastereo- and enantiomeric excesses of the isolated compound **4a** in the presence of the catalyst **3d**, we decided to select it for further optimization studies that included the analysis of additive and solvents effects. Co-catalyst screening (Table 1, entries 7–9) indicated that both benzoic and acetic acid promoted the cyclization to **4a**, with acetic acid providing better conversion (Table 1, entry 9). Subsequent solvent screening (Table 1, entries 9–14) performed in the presence of acetic acid as the cocatalyst indicated dichloromethane or toluene as the best reaction medium, with toluene providing higher diastereoselectivity (Table 1, entry 14). The use of molecular sieves increased the efficiency of the studied sample (Table 1 entries 15 and 16), with the effect being particularly pronounced in the case of toluene (Table 1, entry 16).

**Table 1:**
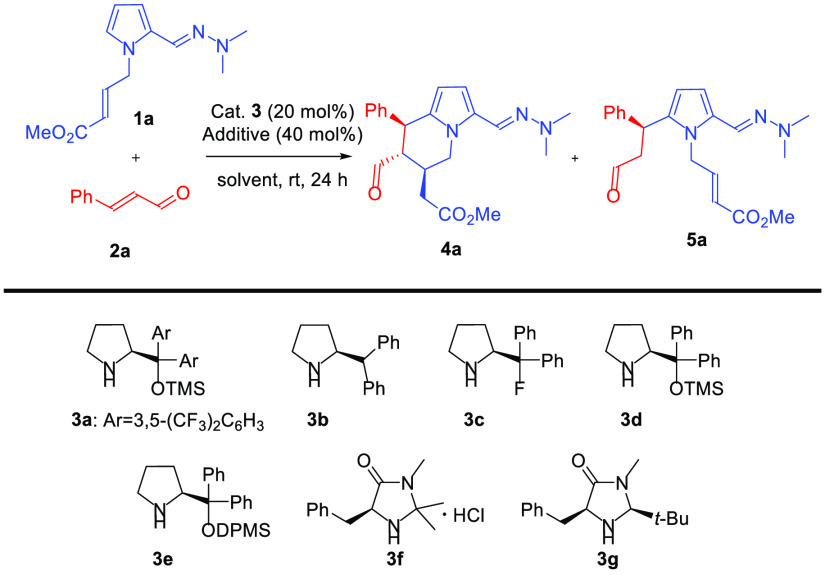
Aminocatalytic Asymmetric Synthesis of Tetrahydroindolizine **4a**: Optimization Studies^a^

	cat.	add.	solvent	conv. **4a**/**5a**[%]^b^	dr **4a**^c^	er **4a**^d^
1	**3a**	TFA	CH_2_Cl_2_	0:84		
2	**3b**	TFA	CH_2_Cl_2_	0:16		
3	**3c**	TFA	CH_2_Cl_2_	10:60	n.d.	n.d.
4	**3d**	TFA	CH_2_Cl_2_	25:35	40:1	>99:1
5	**3e**	TFA	CH_2_Cl_2_	28:34	25:1	99:1
6	**3f**		CH_2_Cl_2_	0:95		
7	**3g**	TFA	CH_2_Cl_2_	0:95		
8	**3d**	BzOH	CH_2_Cl_2_	40:0	32:1	97:3
9	**3d**	AcOH	CH_2_Cl_2_	58:0	25:1	98:2
10	**3d**	AcOH	CHCl_3_	15:0	n.d.	n.d.
11	**3d**	AcOH	DCE	39:0	26:1	98:2
12	**3d**	AcOH	PhCH_3_	37:0	>50:1	98:2
13	**3d**	AcOH	CH_3_CN	62:14	21:1	98:2
14	**3d**	AcOH	EtOH	>99:0	6.5:1	96:4
15^e^	**3d**	AcOH	CH_2_Cl_2_	68:0	18:1	98:2
16^e^	**3d**	AcOH	PhCH_3_	>99:0 (97)	>50:1	98:2

a Reactions were performed on a 0.05 mmol scale using **1a** (1.2 equiv) and **2a** (1 equiv) in 0.2 mL of the solvent for 24 h.

b Conversion was determined by ^1^H NMR of a crude reaction mixture. Isolated yield is given in parentheses.

c Determined by ^1^H NMR of a crude reaction mixture.

d Determined by chiral stationary phase UPC^2^.

e Reaction carried out in the presence of 3 Å molecular sieves.

Having an optimized synthesis procedure, we proceeded to determine the applicability range of the developed aminocatalytic reaction of the hydrazone pyrrole derivative **1** with α,β- unsaturated aldehydes **2a**–**o** (Table 2). It was found that regardless of the position and the electronic nature of the substituent on the phenyl ring of the aromatic α,β-unsaturated aldehydes **2a**–**k** (Table 2, entries 2–11, respectively), all target compounds **4a**–**k** were obtained in high yields and in a highly diastereo- and enantioselective manner. Furthermore, optimized reaction conditions were successfully applied to aliphatic enals **2l**–**o** (Table 2, entries 12–15, respectively), with the reaction outcome being unbiased toward both the length of the aliphatic chain (Table 2, entries 12 and 13) as well as the presence of functional groups (Table 2, entries 14 and 15). The reaction proved readily scalable, with very good results obtained for aldehyde **2a** at 1 mmol scale using only 5 mol % catalyst **3d** (Table 2, entry 16). During further studies, morpholine-derived hydrazone **1b** was employed, providing product **4p** with comparable results (Table 2, entry 17).

**Table 2:**
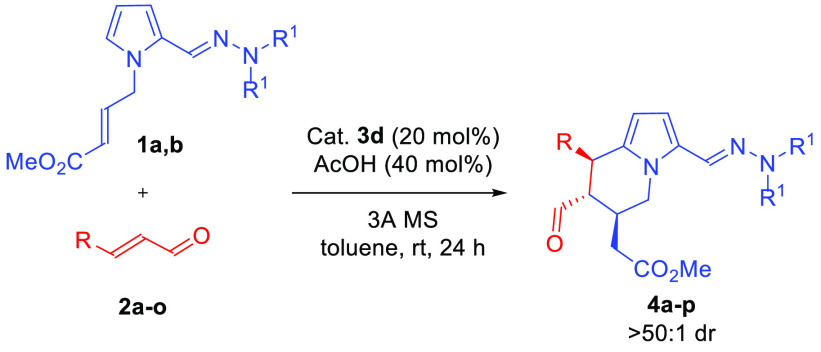
Aminocatalytic Asymmetric Synthesis of Tetrahydroindolizines **4**: Scope Studies^a^

	R	R^1^	R^1^	yield [%]^b^	er^c^
1	Ph	CH_3_	CH_3_	97	98:2
2	4-MeOC_6_H_4_	CH_3_	CH_3_	89	98:2
3	3-MeOC_6_H_4_	CH_3_	CH_3_	78	98:2
4	2-MeOC_6_H_4_	CH_3_	CH_3_	88	96:4
5	4-CNC_6_H_4_	CH_3_	CH_3_	97	>99:1
6	4-CF_3_C_6_H_4_	CH_3_	CH_3_	87	>99:1
7	4-NO_2_OC_6_H_4_	CH_3_	CH_3_	93	98:2
8	4-MeC_6_H_4_	CH_3_	CH_3_	83	98:2
9	4-ClC_6_H_4_	CH_3_	CH_3_	87	98:2
10	2,4-Cl_2_C_6_H_3_	CH_3_	CH_3_	92	99:1
11	2-naphthyl	CH_3_	CH_3_	89	97:3
12^d^	Me	CH_3_	CH_3_	76	99:1
13^e^	Et	CH_3_	CH_3_	72	>99:1
14^e^	(*Z*)-3-hexenyl	CH_3_	CH_3_	56	>99:1
15^e^	BnOCH_2_	CH_3_	CH_3_	43	98:2
16^f^	Ph	CH_3_	CH_3_	77	95:5
17^g^	Ph	–(CH_2_)_2_O(CH_2_)_2_–		86	95:5

a Reactions were performed on a 0.1 mmol scale using **1** (1.2 equiv), **2a**–**o** (1 equiv), catalyst **3d** (20 mol %), and acetic acid (40 mol %) in toluene (0.4 mL) at room temperature for 24 h.

b The isolated yield is given.

c Determined by chiral stationary phase UPC^2^.

d The reaction was carried out with five equivalents of aldehyde **2l** at 0 °C for 2 days and overnight at room temperature.

e The reaction was carried out with two portions of aldehydes **2l–o** at 0 °C for 2 days and overnight at room temperature.

f The reaction was proceeded on a 1 mmol scale using **1a** (1.2 equiv), **2a** (1 equiv), catalyst **3d** (5 mol %), and acetic acid (40 mol %) in toluene (4 mL) at room temperature for 24 h.

g The reaction was performed using **1b** (1.2 equiv), **2a** (1 equiv), catalyst **3d** (20 mol %), and acetic acid (40 mol %) in toluene (0.4 mL) at room temperature for 96 h.

In the course of further studies, cycloadduct **4a** was subjected to chemoselective transformations (Scheme 3). Initially, unmasking of the hydrazone moiety using *meta*-chloroperoxybenzoic acid in dichloromethane was performed, affording nitrile **6a** in 96% yield. Nitrile **6a** was employed in the further transformations. Reductive amination of the aldehyde group in **6a** gave product **7a** in a good yield. The reaction was realized under mild conditions using sodium cyanoborohydride as the reduction reagent. Furthermore, products **8a** and **9a** containing an additional six-membered ring were efficiently obtained following a two-step protocol performed in a one-pot sequence. δ-Lactam **8a** was obtained in 45% yield, while the reaction providing δ-lactone **9a** was slightly more efficient (57% yield). All products were obtained as single diastereoisomers.

**Scheme 3:**
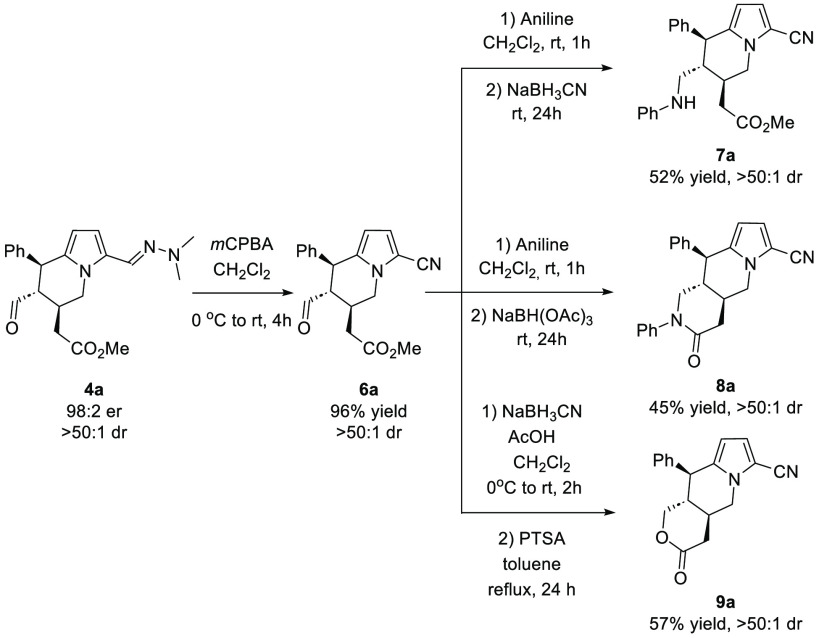
Stereoselective Transformations of Tetrahydroindolizine **4a**

The absolute configuration of product **4g** was confirmed by the X-ray structural analysis (for details, see the SI).^18^ The stereochemistry of compounds **4a**–**o** has been assigned by analogy assuming the same reaction mechanism. The mechanism and stereochemical model of the studied reaction was proposed (Scheme 4). The catalytic cycle of the reaction is initiated with the condensation of aminocatalyst **3d** with α,β-unsaturated aldehydes **2**, which results in the formation of the appropriate iminium ion **10**. The Friedel–Crafts reaction of hydrazone **1a** with iminium ion **10** is facilitated by the donating effect provided by the hydrazone moiety in **1a**. The stereochemical outcome of the addition is governed by the steric shielding principle exerted by the bulky substituent present in the C-2 position of the pyrrolidine ring in **10**. Subsequently, the cyclization of **11** takes place via the enamine-mediated Michael reaction. It is postulated that the reaction proceeds via a chairlike transition state the controls the diastereoselectivity of the process. Finally, the hydrolysis of intermediate **12** occurs, releasing the catalyst **3d** with the restoration of the carbonyl moiety in **4**.

**Scheme 4:**
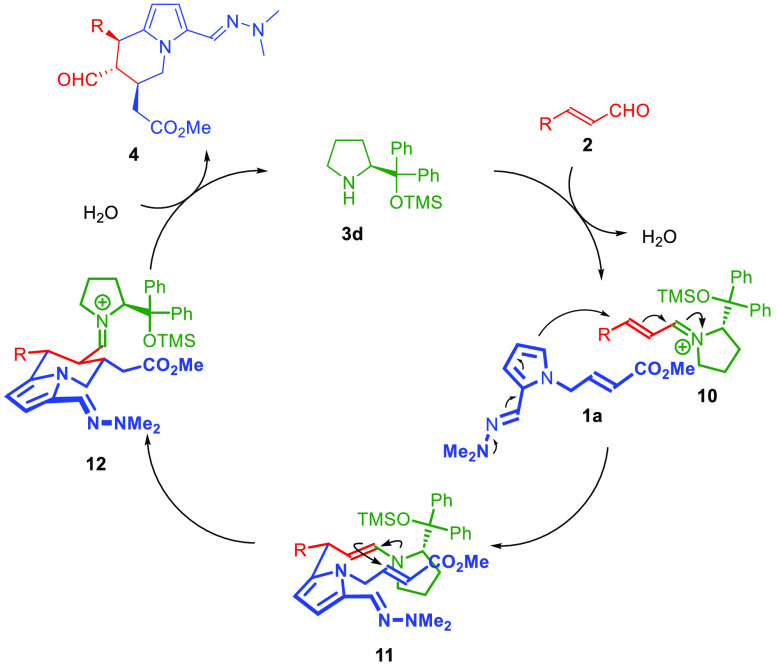
Aminocatalytic Asymmetric Synthesis of Tetrahydroindolizines **4**: Scope Studies

In conclusion, a new aminocatalytic reaction of a pyrrol-derived hydrazone bearing a Michael acceptor moiety in the *N*-alkyl side chain with α,β-unsaturated aldehydes was developed. The synthesis of the target products proceeded by Friedel–Crafts alkylation and subsequent cyclization via a Michael reaction. Target products were obtained with very good yields and excellent diastereo- and enantioselectivities. The possibility of using the obtained desired products for further modifications leading to useful polycyclic compounds was also demonstrated.

## Data Availability

The data underlying this study are available in the published article and its Supporting Information.

## Supporting Information Available

Experimental procedures, characterization of the products, crystal and X-ray data for **4g**, NMR data, and UPC^2^ traces (PDF)
