# Supplementary material for: Hydrazone Activation in the Aminocatalytic Cascade Reaction for the Synthesis of Tetrahydroindolizines
Source: Org Lett. 2024 Jan 24;26(4):814–8. doi: 10.1021/acs.orglett.3c03911 (PMC10845150; doi:10.1021/acs.orglett.3c03911)
Supplement: Supplementary file 1 — ol3c03911_si_001.pdf [file ol3c03911_si_001.pdf]

Hydrazone Activation in the Aminocatalytic Cascade Reactivity for the Synthesis of Tetrahydroindolizines

Justyna Kowalska[a], Beata Łukasik[a], Sebastian Frankowski[a] and Łukasz Albrecht[a*]

[a] Institute of Organic Chemistry, Department of Chemistry

Lodz University of Technology

Zeromskiego 116, 90-924 Lodz, Poland

E-mail: lukasz.albrecht@p.lodz.pl

Contents

1. General methods S2
2. Synthesis of substrates S3
   2.1 Synthesis of (*E*)-methyl 4-(2-((*E*)-(2,2-dimethylhydrazono)methyl)-1*H*-pyrrol-1-yl)but-2-enoate (**1a**) S3
   2.2. Synthesis of (*E*)-methyl 4-(2-((*E*)-(morpholinoimino)methyl)-1*H*-pyrrol-1-yl)- but-2-enoate (**1b**) S3
3. Aminocatalytic asymmetric synthesis of tetrahydroindolizines **4** – general procedure S5
4. Synthesis of methyl 2-((6*S*,7*S*,8*S*)-3-((*E*)-(2,2-dimethylhydrazono)methyl)-7-formyl-8-phenyl-5,6,7,8-tetrahydroindolizin-6-yl)acetate (**4a**) – 1 mmol scale experiment S12
5. Synthesis of methyl 2-((6*S*,7*S*,8*S*)-3-cyano-7-formyl-8-phenyl-5,6,7,8-tetrahydroindolizin-6-yl)acetate (**6a**) S13
6. Synthesis of methyl 2-((6*S*,7*S*,8*S*)-3-cyano-8-phenyl-7-((phenylamino)methyl)-5,6,7,8-tetrahydroindolizin-6-yl)acetate (**7a**) S14
7. Synthesis of (4a*S*,10*S*,10a*S*)-3-oxo-2,10-diphenyl-1,2,3,4,4a,5,10,10a-octahydropyrrolo[1,2-*b*][2,6]naphthyridine-7-carbonitrile (**8a**) S15
8. Synthesis of (4a*S*,10*S*,10a*S*)-3-oxo-10-phenyl-3,4,4a,5,10,10a-hexahydro-1*H*-pyrano[4,3-*f*]indolizine-7-carbonitrile (**9a**) S16
9. Crystal and X-ray data for methyl 2-((6*S*,7*S*,8*S*)-3-((*E*)-(2,2-dimethylhydrazono)-methyl)-7-formyl-8-(4-nitrophenyl)-5,6,7,8-tetrahydroindolizin-6-yl)acetate (**4g**) S17
10. NMR data S19
11. UPC^2 or HPLC traces S41

1. General methods

Unless otherwise noted, analytical grade solvents and commercially available reagents were used without further purification. Analytical thin layer chromatography (TLC) was performed using pre-coated aluminum-backed plates (Merck Kieselgel 60 F254) and visualized by ultraviolet irradiation. Silica gel (Silica gel 60, 230-400 mesh, Fluka) was used for column chromatography. NMR spectra were acquired on a Bruker Ultra Shield 700 instrument, running at 700 MHz for ^{1}H and 176 MHz for ^{13}C or on a Jeol 400YH instrument, running at 400 MHz for ^{1}H and 101 MHz for ^{13}C, respectively. Chemical shifts (δ) were reported as part per million (ppm) in δ scale relative to residual solvent signals ($CDCl_3$: 7.26 ppm for ^{1}H NMR, 77.00 ppm for ^{13}C NMR). Multiplicities were given as: s (singlet); br s (broaded singlet); d (doublet); dd (doublet of doublets); ddd (doublet of doublet of doublets). Coupling constants (*J*) were reported in Hertz (Hz). High-resolution mass spectra (HRMS) were obtained on Bruker ESI-Q-TOF Impact II spectrometer using electrospray (ESI+) ionization. Optical rotations were measured on a Perkin-Elmer 241 polarimeter and $[\alpha]_D$ values are given in deg•cm•g^{-1}•dm^{-1}; concentration *c* is listed in g•(100 mL)$^{-1}$. The enantiomeric ratio (er) of the products were determined by Ultra Performance Convergence Chromatography (UPCC) or HPLC using Daicel Chiralpak IA, IB and IC columns as chiral stationary phases. IR spectra were recorded on a Brucker Alpha FT-IR spectrometer.

2. Synthesis of substrates

2.1 Synthesis of (*E*)-methyl 4-(2-((*E*)-(2,2-dimethylhydrazono)methyl)-1*H*-pyrrol-1-yl)but-2-enoate (1a)

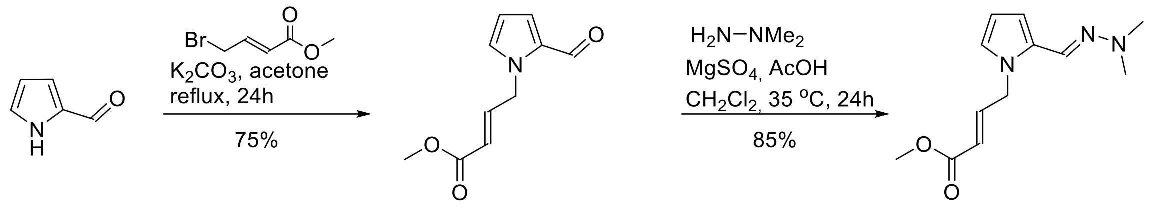

1a

Scheme S1.

The reaction was carried out following the modified literature procedure.[1] To a solution of 2-pyrrolecarbaldehyde (1.0 equiv., 8 mmol, 768 mg) in acetone (64 mL), methyl *trans*-4-bromo-2-butenoate (3.0 equiv., 24 mmol, 2.4 mL) and K_2CO_3 (3.6 equiv., 28.8 mmol, 3.97 g) were added and the resulting mixture was refluxed overnight. After this time the reaction mixture was allowed to cool down to room temperature and toluene (16 mL) was added. The precipitate was filtered off and the filtrate was concentrated in vacuo and purified by column chromatography (petroleum ether/ethyl acetate 6:1) to give (*E*)-methyl 4-(2-formyl-1*H*-pyrrol-1-yl)but-2-enoate in 75% yield as a yellow solid. Its NMR spectra were in accordance with the literature data.[1]

To the solution of (*E*)-methyl 4-(2-formyl-1*H*-pyrrol-1-yl)but-2-enoate (1.0 equiv., 5.85 mmol, 1.13 g) in dichloromethane (24 mL), $MgSO_4$ (5.0 equiv., 29.2 mmol, 4.8 g), acetic acid (3 mL) and *N*,*N*-dimethylhydrazine (3.0 equiv., 17.5 mmol, 1.9 mL) were added and the resulting mixture was refluxed overnight. After this time the reaction mixture was allowed to cool down to room temperature and precipitate was filtered off. The filtrate was concentrated in vacuo and purified by column chromatography (petroleum ether/ethyl acetate 3:1) to give **1** in 85% yield as a green oil. ^{1}H NMR (700 MHz, $CDCl_3$) δ 7.05 (dt, J = 15.6 Hz, J = 4.7 Hz, 1H), 6.58 (dd, J = 2.7 Hz, J = 1.8 Hz, 1H), 6.25 (dd, J = 3.6 Hz, J = 1.8 Hz, 1H), 6.15 (dd, J = 3.7 Hz, J = 2.7 Hz, 1H), 5.53 (dt, J = 15.6 Hz, J = 1.9 Hz, 1H), 5.10 (dd, J = 4.7 Hz, J = 2.0 Hz, 2H), 3.69 (s, 3H), 2.81 (s, 6H). ^{13}C NMR (176 MHz, $CDCl_3$) δ 166.7, 145.6, 129.3, 128.5, 124.5, 121.2, 112.1, 108.7, 51.6, 49.3, 43.2 (2C).

2.2 Synthesis of (*E*)-methyl 4-(2-((*E*)-(morpholinoimino)methyl)-1*H*-pyrrol-1-yl)but-2-enoate (1b)

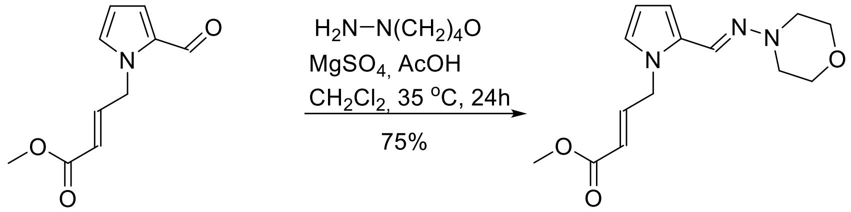

1b

Scheme S2.

[1] Srinivasulu, V.; Shehadeh, I.; Khanfar, M. A.; Malik, O. G.; Tarazi, K.; Abu-Yousef, I. A.; Sebastian, A.; Baniowda, N.; O'Connor, M. J.; Al-Tel, T. H. *J. Org. Chem.* **2019**, *84*, 934–948.

To the solution of (*E*)-methyl 4-(2-formyl-1*H*-pyrrol-1-yl)but-2-enoate (1.0 equiv., 0.5 mmol, 0.1 g) in dichloromethane (2 mL), $MgSO_4$ (5.0 equiv., 2.5 mmol, 0.4 g), acetic acid (0.25 mL) and 4-aminomorpholine (2.0 equiv., 1 mmol, 152 μL) were added and the resulting mixture was refluxed overnight. After this time the reaction mixture was allowed to cool down to room temperature and precipitate was filtered off. The filtrate was concentrated in vacuo and purified by column chromatography (petroleum ether/ethyl acetate 3:1) to give **1b** in 75% yield (108 mg) as a yellowish oil. ^{1}H NMR (700 MHz, $CDCl_3$) δ 7.58 (s, 1H), 7.04 (dt, J = 15.6 Hz, J = 4.6 Hz, 1H), 6.74 – 6.58 (m, 1H), 6.41 – 6.28 (m, 1H), 6.17 (dd, J = 3.7 Hz, J = 2.7 Hz, 1H), 5.51 (dt, J = 15.6 Hz, J = 1.9 Hz, 1H), 5.12 (dd, J = 4.7 Hz, J = 1.9 Hz, 2H), 4.01 – 3.79 (m, 4H), 3.70 (s, 3H), 3.10 – 2.96 (m, 4H). ^{13}C NMR (176 MHz, $CDCl_3$) δ 166.5, 145.2, 131.6, 128.3, 125.4, 121.2, 113.8, 108.8, 66.4 (2C), 52.3 (2C), 51.5, 49.4. HRMS (ESI) m/z [M+H]+ Calcd. for $C_{14}H_{20}N_3O_3$: 278.1499 ; found: 278.1498.

3. Aminocatalytic asymmetric synthesis of tetrahydroindolizines 4 - general procedure

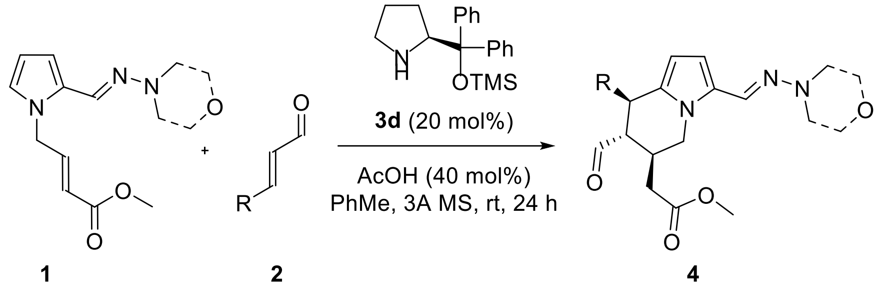

Scheme S3.

In an ordinary 4 mL glass vial, equipped with a magnetic stirring bar and a screw cap, the corresponding *N*,*N*-dialkylhydrazone **1** (1.2 equiv. 0.12 mmol) and appropriate *α*,*β*-unsaturated aldehyde **2** (1.0 equiv., 0.1 mmol) were dissolved in toluene (0.4 mL). (*S*)-(–)-*α*,*α*-Diphenyl-2-pyrrolidinemethanol trimethylsilyl ether (**3d**) (20 mol%, 0.01 mmol, 6.5 mg) and acetic acid (40 mol%, 0.02 mmol, 2.4 μL) were added and resulting mixture was stirred overnight. Crude products **4** were purified by the column chromatography on silica gel using petroleum ether/ethyl acetate 8:1 as an eluent.

Methyl 2-((6*S*,7*S*,8*S*)-3-((*E*)-(2,2-dimethylhydrazono)methyl)-7-formyl-8-phenyl-5,6,7,8-tetrahydroindolizin-6-yl)acetate (**4a**)

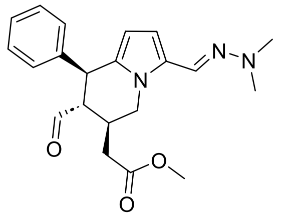

Following the general procedure, compound **4a** was isolated in 97% yield (35.6 mg) as a light-yellow oil. ^{1}H NMR (700 MHz, $CDCl_3$) *δ* 9.60 (d, *J* = 3.4 Hz, 1H), 7.33 – 7.29 (m, 3H), 7.27 (d, *J* = 1.3 Hz, 1H), 7.24 (dd, *J* = 8.2 Hz, *J* = 1.4 Hz, 2H), 6.16 (d, *J* = 3.7 Hz, 1H), 5.48 (dd, *J* = 3.7 Hz, *J* = 1.3 Hz, 1H), 4.78 (dd, *J* = 13.6 Hz, *J* = 5.1 Hz, 1H), 4.35 (d, *J* = 10.9 Hz, 1H), 4.01 (dd, *J* = 13.6 Hz, *J* = 10.2 Hz, 1H), 3.69 (s, 3H), 2.93 (tdt, *J* = 10.4 Hz, *J* = 7.0 Hz, *J* = 5.5 Hz, 1H), 2.88 – 2.83 (m, 1H), 2.84 (s, 6H), 2.52 (dd, *J* = 16.3 Hz, *J* = 5.7 Hz, 1H), 2.42 (dd, *J* = 16.3 Hz, 7.1 Hz, 1H). ^{13}C NMR (176 MHz, $CDCl_3$) δ 203.1, 171.9, 140.6, 133.7, 129.3, 128.9 (4C), 128.7, 127.6, 111.4, 107.2, 57.1, 52.0, 48.9, 43.5, 43.3 (2C), 36.4, 32.6. The er was determined by UPC^2 using a chiral Chiralpack IA column gradient from 100% CO_2 up to 40%; *i*-PrOH, flow rate = 2.2 mL/min τ_{major} = 3.53 min, τ_{minor} = 3.59 min, (98:2 er). $[\alpha]_D^{21}$ = + 44,8 (*c* 1.0, CH_2Cl_2). IR: $\tilde{\nu}$ = 2955, 2820, 1720, 1439, 1158, 1013, 753, 699 cm^{-1}. HRMS (ESI) m/z $[M+H]^+$ Calcd. for $C_{21}H_{26}N_3O_3^+$: 368.1969; found: 368.1967.

Methyl 2-((6*S*,7*S*,8*S*)-3-((*E*)-(2,2-dimethylhydrazono)methyl)-7-formyl-8-(4-methoxyphenyl)-5,6,7,8-tetrahydroindolizin-6-yl)acetate (**4b**)

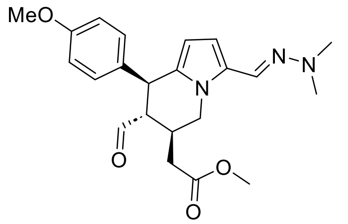

Following the general procedure, compound **4b** was isolated in 89% yield (35.4 mg) as a light-yellow oil. ^{1}H NMR (700 MHz, $CDCl_3$) *δ* 9.58 (d, *J* = 3.5 Hz, 1H), 7.29 (s, 1H), 7.17 – 7.14 (m, 2H), 6.86 – 6.83 (m, 2H), 6.16 (d, *J* = 3.7 Hz, 1H), 5.48 (dd, *J* = 3.7 Hz, *J* = 1.3 Hz, 1H), 4.77 (dd, *J* = 13.6 Hz, *J* = 5.2 Hz, 1H), 4.30 (d, *J* = 11.0 Hz, 1H), 3.98 (dd, *J* = 13.6 Hz, *J* = 10.3 Hz, 1H), 3.79 (s, 3H), 3.69 (s, 3H), 2.92 (tdt, *J* = 10.4 Hz, *J* = 7.1 Hz, *J* = 5.5 Hz, 1H), 2.83 (s, 6H), 2.79 (ddd, *J* = 11.1 Hz, *J* = 10.1 Hz, *J* = 3.4 Hz, 1H), 2.51 (dd, *J* = 16.3 Hz, *J* = 5.8 Hz, 1H), 2.41 (dd, *J* = 16.3 Hz, *J* = 7.1 Hz, 1H). ^{13}C NMR (176 MHz, $CDCl_3$) *δ* 203.3, 172.0, 159.0, 134.1, 132.5,

129.9 (2C), 129.4, 128.7, 114.3 (2C), 111.4, 107.1, 57.2, 55.4, 52.0, 48.9, 43.4 (2C), 42.8, 36.4, 32.6. The er was determined by UPC^2 using a chiral Chiralpack IB column gradient from 100% CO_2 up to 40%; *i*-PrOH, flow rate = 2.2 mL/min τ_{major} = 3.59 min, τ_{minor} = 3.72 min, (98:2 er). $[\alpha]_D^{21}$ = - 230,1 (*c* 1.0, CH_2Cl_2). IR: $\tilde{\nu}$ = 2952, 2838, 1722, 1513, 1438, 1248, 1176, 1160, 1015, 761 cm^{-1}. HRMS (ESI) m/z $[M+H]^+$ Calcd. for $C_{22}H_{28}N_3O_4^+$: 398.2074; found: 398.2075.

Methyl 2-((6*S*,7*S*,8*S*)-3-((*E*)-(2,2-dimethylhydrazono)methyl)-7-formyl-8-(3-methoxyphenyl)-5,6,7,8-tetrahydroindolizin-6-yl)acetate (**4c**)

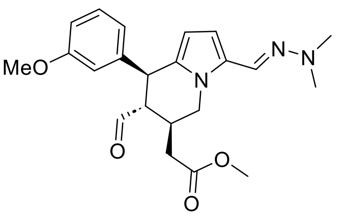

Following the general procedure, compound **4c** was isolated in 78% yield (31.0 mg) as a light-yellow oil. ^{1}H NMR (700 MHz, $CDCl_3$) δ 9.60 (d, *J* = 3.3 Hz, 1H), 7.29 (s, 1H), 7.22 (t, *J* = 7.9 Hz, 1H), 6.83 (d, *J* = 7.7 Hz, 1H), 6.81 – 6.77 (m, 2H), 6.17 (d, *J* = 3.7 Hz, 1H), 5.53 (dd, *J* = 3.7 Hz, *J* = 1.2 Hz, 1H), 4.77 (dd, *J* = 13.6, *J* = 5.1 Hz, 1H), 4.32 (d, *J* = 10.8 Hz, 1H), 3.99 (dd, *J* = 13.6 Hz, *J* = 10.2 Hz, 1H), 3.77 (s, 3H), 3.69 (s, 3H), 2.92 (tdt, *J* = 10.3 Hz, *J* = 6.9 Hz, *J* = 5.5 Hz, 1H), 2.84 (s, 6H), 2.84 – 2.80 (m, 1H), 2.52 (dd, *J* = 16.3 Hz, *J* = 5.7 Hz, 1H), 2.42 (dd, *J* = 16.3 Hz, *J* = 7.0 Hz, 1H). ^{13}C NMR (176 MHz, $CDCl_3$) δ 203.0, 172.0, 160.0, 142.2, 133.4, 129.9, 129.3, 128.7, 121.2, 114.6, 112.9, 111.4, 107.2, 57.0, 55.4, 52.0, 48.9, 43.6, 43.4 (2C), 36.4, 32.6. The er was determined by UPC^2 using a chiral Chiralpack IA column gradient from 100% CO_2 up to 40%; *i*-PrOH, flow rate = 2.2 mL/min τ_{major} = 3.70 min, τ_{minor} = 3.81 min, (98:2 er). $[\alpha]_D^{21}$ = - 159,0 (*c* 1.0, CH_2Cl_2). IR: $\tilde{\nu}$ = 2953, 2820, 1719, 1484, 1437, 1401, 1273, 1245, 1157, 1015, 756, 694 cm^{-1}. HRMS (ESI) m/z $[M+H]^+$ Calcd. for $C_{22}H_{28}N_3O_4^+$: 398.2074; found: 398.2084.

Methyl 2-((6*S*,7*S*,8*S*)-3-((*E*)-(2,2-dimethylhydrazono)methyl)-7-formyl-8-(2-methoxyphenyl)-5,6,7,8-tetrahydroindolizin-6-yl)acetate (**4d**)

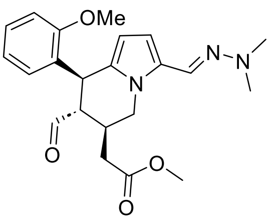

Following the general procedure, compound **4d** was isolated in 88% yield (35.0 mg) as a light-yellow oil. ^{1}H NMR (700 MHz, $CDCl_3$) δ 9.59 (d, *J* = 3.6 Hz, 1H), 7.31 (s, 1H), 7.23 (ddd, *J* = 8.2 Hz, *J* = 7.4 Hz, *J* = 1.7 Hz, 1H), 7.12 (dd, *J* = 7.5 Hz, *J* = 1.7 Hz, 1H), 6.91 – 6.86 (m, 2H), 6.18 (d, *J* = 3.7 Hz, 1H), 5.54 (dd, *J* = 3.6 Hz, *J* = 1.2 Hz, 1H), 4.86 – 4.78 (m, 2H), 3.95 (dd, *J* = 13.5 Hz, *J* = 10.1 Hz, 1H), 3.77 (s, 3H), 3.67 (s, 3H), 2.93 (dqd, *J* = 10.0 Hz, *J* = 4.8 Hz *J* = 2.0 Hz, 1H), 2.83 (s, 6H), 2.78 (td, *J* = 9.9 Hz, *J* = 3.6 Hz, 1H), 2.51 (dd, *J* = 16.3 Hz, *J* = 5.7 Hz, 1H), 2.37 (dd, *J* = 16.3 Hz, *J* = 7.4 Hz, 1H). ^{13}C NMR (176 MHz, $CDCl_3$) δ 202.9, 172.1, 157.1, 133.3, 129.8, 129.8, 129.6, 128.6, 128.3, 121.1, 111.5, 111.1, 106.5, 56.9, 55.6, 52.0, 48.5, 43.4 (2C), 36.5, 35.8, 32.3. The er was determined by UPC^2 using a chiral Chiralpack IC column gradient from 100% CO_2 up to 40%; *i*-PrOH, flow rate = 2.2 mL/min τ_{major} = 3.09 min, τ_{minor} = 3.18 min, (96:4 er). $[\alpha]_D^{21}$ = - 65,0 (*c* 1.0, CH_2Cl_2). IR: $\tilde{\nu}$ = 2951, 2846, 1719, 1491, 1460, 1438, 1236, 1192, 1158, 1018, 754 cm^{-1}. HRMS (ESI) m/z $[M+H]^+$ Calcd. for $C_{22}H_{28}N_3O_4^+$: 398.2074; found: 398.2079.

Methyl 2-((6*S*,7*S*,8*S*)-8-(4-cyanophenyl)-3-((*E*)-(2,2-dimethylhydrazono)methyl)-7-formyl-5,6,7,8-tetrahydroindolizin-6-yl)acetate (**4e**)

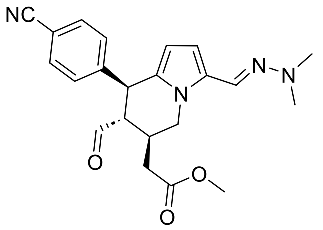

Following the general procedure, compound **4e** was isolated in 97% yield (38.0 mg) as a light-yellow oil. ^{1}H NMR (700 MHz, $CDCl_3$) δ 9.62 (d, *J* = 3.2 Hz, 1H), 7.63 – 7.59 (m, 2H), 7.38 – 7.35 (m, 2H), 7.26 (s, 1H), 6.15 (d, *J* = 3.7 Hz, 1H), 5.42 (dd, *J* = 3.7 Hz, *J* = 1.2 Hz, 1H), 4.76 (dd, *J* = 13.7 Hz, *J* = 5.0 Hz, 1H), 4.46 (d, *J* = 10.6 Hz, 1H), 4.04 (dd, *J* = 13.7 Hz, *J* = 10.1 Hz, 1H), 3.69 (s, 3H), 2.93 – 2.87 (m, 1H), 2.85 (s, 6H), 2.85 – 2.81 (m, 1H), 2.51 (dd, *J* = 16.4 Hz, *J* = 5.8 Hz, 1H), 2.46 (dd, *J* = 16.4 Hz, *J* = 6.7 Hz, 1H). ^{13}C NMR (176 MHz, $CDCl_3$) δ 202.1, 171.7, 146.6, 132.7 (2C), 131.8, 129.8 (2C), 129.2, 128.6, 118.7, 111.6, 111.3, 107.4, 56.9, 52.1, 48.8, 43.2 (2C), 43.0, 36.2, 32.8. The er was determined by UPC^2 using a chiral Chiralpack IB column gradient from 100% CO_2 up to 40%; *i*-PrOH, flow rate = 2.2 mL/min τ_{major} = 3.88 min, τ_{minor} = 4.21 min, (99,5:0,5 er). $[\alpha]_D^{21}$ = - 103,2 (*c* 1.0, CH_2Cl_2). IR: $\tilde{\nu}$ = 2954, 2855, 2230, 1725, 1475, 1438, 1018, 879, 750, 556 cm^{-1}. HRMS (ESI) m/z $[M+H]^+$ Calcd. for $C_{22}H_{25}N_4O_3^+$: 393.1921; found: 393.1919.

Methyl 2-((6*S*,7*S*,8*S*)-3-((*E*)-(2,2-dimethylhydrazono)methyl)-7-formyl-8-(4-(trifluoro-methyl)phenyl)-5,6,7,8-tetrahydroindolizin-6-yl)acetate (**4f**)

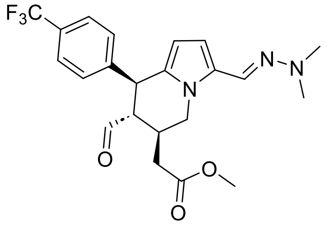

Following the general procedure, compound **4f** was isolated in 87% yield (37.9 mg) as a light-yellow oil. ^{1}H NMR (700 MHz, $CDCl_3$) δ 9.62 (d, *J* = 3.2 Hz, 1H), 7.57 (d, *J* = 8.0 Hz, 2H), 7.37 (d, *J* = 8.1 Hz, 2H), 7.28 (s, 1H), 6.16 (d, *J* = 3.7 Hz, 1H), 5.45 (dd, *J* = 3.7, *J* = 1.3 Hz, 1H), 4.77 (dd, *J* = 13.6 Hz, *J* = 5.0 Hz, 1H), 4.45 (d, *J* = 10.6 Hz, 1H), 4.04 (dd, *J* = 13.6 Hz, *J* = 10.1 Hz, 1H), 3.69 (s, 3H), 2.95 – 2.89 (m, 1H), 2.88 – 2.86 (m, 1H), 2.85 (s, 6H), 2.53 (dd, *J* = 16.4 Hz, *J* = 5.8 Hz, 1H), 2.45 (dd, *J* = 16.4 Hz, *J* = 6.8 Hz, 1H). ^{13}C NMR (176 MHz, $CDCl_3$) δ 202.4, 171.8, 145.1, 132.4, 129.92 (q, *J* = 32.4 Hz), 129.3 (2C), 129.1, 128.9, 125.9 (q, *J* = 3.6 Hz), 124.9, 123.4, 111.4, 107.4, 57.0, 52.1, 48.9, 43.3 (2C), 43.0, 36.3, 32.8. The er was determined by UPC^2 using a chiral Chiralpack IB column gradient from 100% CO_2 up to 40%; *i*-PrOH, flow rate = 2.2 mL/min τ_{major} = 2.95 min, τ_{minor} = 3.29 min, (99,5:0,5 er). $[\alpha]_D^{21}$ = + 106,4 (*c* 1.0, CH_2Cl_2). IR: $\tilde{\nu}$ = 2924, 2852, 1732, 1477, 1439, 1323, 1199, 1162, 1122, 1066, 1020, 878, 750 cm^{-1}. HRMS (ESI) m/z $[M+H]^+$ Calcd. for $C_{22}H_{25}F_3N_3O_3^+$: 436.1842; found: 436.1842.

Methyl 2-((6*S*,7*S*,8*S*)-3-((*E*)-(2,2-dimethylhydrazono)methyl)-7-formyl-8-(4-nitrophenyl)-5,6,7,8-tetrahydroindolizin-6-yl)acetate (**4g**)

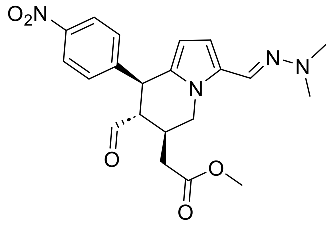

Following the general procedure, compound **4g** was isolated in 93% yield (38.3 mg) as a light-yellow oil. ^{1}H NMR (700 MHz, $CDCl_3$) δ 9.65 (d, *J* = 3.1 Hz, 1H), 8.19 – 8.16 (m, 2H), 7.44 – 7.41 (m, 2H), 7.27 (s, 1H), 6.16 (d, *J* = 3.7 Hz, 1H), 5.43 (dd, *J* = 3.7 Hz, *J* = 1.2 Hz, 1H), 4.77 (dd, *J* = 13.7 Hz, *J* = 4.9 Hz, 1H), 4.53 (d, *J* = 10.3 Hz, 1H), 4.06 (dd, *J* = 13.7 Hz, *J* = 9.9 Hz, 1H), 3.70 (s, 3H), 2.94 – 2.90 (m, 1H), 2.89 – 2.86 (m, 1H), 2.85 (s, 6H), 2.53 (dd, *J* = 16.4 Hz, *J* = 5.7 Hz, 1H), 2.47 (dd, *J* = 16.4 Hz, *J* = 6.5 Hz, 1H). ^{13}C NMR (176 MHz, $CDCl_3$) δ 201.9, 171.7, 148.7, 147.5, 131.7, 129.9 (2C), 129.3, 128.6, 124.1 (2C), 111.4, 107.5, 57.0, 52.1, 48.9, 43.3 (2C), 42.8, 36.2, 32.9. The er was determined by UPC^2 using a chiral Chiralpack IB column gradient from 100% CO_2 up to 40%; *i*-PrOH, flow rate = 2.2 mL/min τ_{major} = 4.12 min, τ_{minor} = 4.44 min, (98:2

er). $[\alpha]_D^{21}$ = - 48,6 (c 1.0, CH_2Cl_2). IR: $\tilde{\nu}$ = 2921, 2851, 1725, 1516, 1438, 1343, 1166, 1015, 859, 750, 698 cm^{-1}. HRMS (ESI) m/z $[M+H]^+$ Calcd. for $C_{21}H_{25}N_4O_5^+$: 413.1819; found: 413.1822.

Methyl 2-((6*S*,7*S*,8*S*)-3-((*E*)-(2,2-dimethylhydrazono)methyl)-7-formyl-8-(*p*-tolyl)-5,6,7,8-tetrahydroindolizin-6-yl)acetate (**4h**)

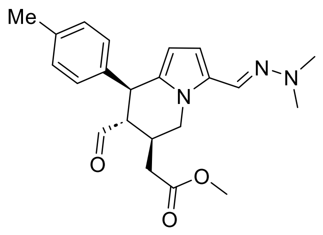

Following the general procedure, compound **4h** was isolated in 83% yield (31.6 mg) as a light-yellow oil. ^{1}H NMR (700 MHz, $CDCl_3$) δ 9.59 (d, J = 3.4 Hz, 1H), 7.29 (s, 1H), 7.12 (d, J = 1.1 Hz, 4H), 6.16 (d, J = 3.7 Hz, 1H), 5.49 (dd, J = 3.7 Hz, J = 1.3 Hz, 1H), 4.77 (dd, J = 13.6 Hz, J = 5.1 Hz, 1H), 4.31 (d, J = 10.9 Hz, 1H), 3.99 (dd, J = 13.6 Hz, J = 10.3 Hz, 1H), 3.69 (s, 3H), 2.92 (tdt, J = 10.4 Hz, J = 7.1 Hz, J = 5.5 Hz, 1H), 2.84 (s, 6H), 2.83 – 2.78 (m, 1H), 2.52 (dd, J = 16.3 Hz, J = 5.7 Hz, 1H), 2.41 (dd, J = 16.3 Hz, J = 7.1 Hz, 1H), 2.33 (s, 3H). ^{13}C NMR (176 MHz, $CDCl_3$) δ 203.2, 172.0, 137.5, 137.2, 134.0, 129.6 (2C), 129.4, 128.7 (2C), 128.7, 111.4, 107.1, 57.1, 52.0, 48.9, 43.4 (2C), 43.1, 36.4, 32.6, 21.2. The er was determined by UPC^2 using a chiral Chiralpack IB column gradient from 100% CO_2 up to 40%; *i*-PrOH, flow rate = 2.2 mL/min τ_{major} = 3.31 min, τ_{minor} = 3.48 min, (98:2 er). $[\alpha]_D^{21}$ = - 43,1 (c 1.0, CH_2Cl_2). IR: $\tilde{\nu}$ = 2952, 2817, 1721, 1512, 1479, 1439, 1195, 1157, 1014, 768 cm^{-1}. HRMS (ESI) m/z $[M+H]^+$ Calcd. for $C_{22}H_{28}N_3O_3^+$: 382.2125; found: 382.2128.

Methyl 2-((6*S*,7*S*,8*S*)-8-(4-chlorophenyl)-3-((*E*)-(2,2-dimethylhydrazono)methyl)-7-formyl-5,6,7,8-tetrahydroindolizin-6-yl)acetate (**4i**)

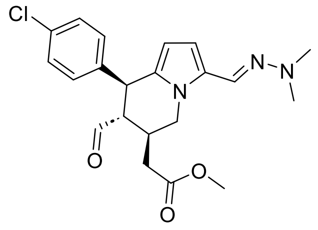

Following the general procedure, compound **4i** was isolated in 87% yield (34.9 mg) as a light-yellow oil. ^{1}H NMR (700 MHz, $CDCl_3$) δ 9.59 (d, J = 3.4 Hz, 1H), 7.28 (d, J = 8.4 Hz, 3H), 7.20 – 7.17 (m, 2H), 6.16 (d, J = 3.7 Hz, 1H), 5.46 (dd, J = 3.7 Hz, J = 1.2 Hz, 1H), 4.76 (dd, J = 13.6 Hz, J = 5.1 Hz, 1H), 4.34 (d, J = 10.9 Hz, 1H), 4.00 (dd, J = 13.7 Hz, J = 10.3 Hz, 1H), 3.69 (s, 3H), 2.95 – 2.86 (m, 1H), 2.84 (s, 6H), 2.82 – 2.77 (m, 1H), 2.51 (dd, J = 16.3 Hz, J = 5.8 Hz, 1H), 2.43 (dd, J = 16.3 Hz, J = 6.9 Hz, 1H). ^{13}C NMR (176 MHz, $CDCl_3$) δ 202.7, 171.8, 139.3, 133.4, 133.0, 130.2 (2C), 129.0 (2C), 129.0, 128.9, 111.3, 107.2, 57.1, 52.0, 48.8, 43.3 (2C), 42.7, 36.3, 32.7. The er was determined by UPC^2 using a chiral Chiralpack IB column gradient from 100% CO_2 up to 40%; *i*-PrOH, flow rate = 2.2 mL/min τ_{major} = 3.50 min, τ_{minor} = 3.88 min, (98:2 er). $[\alpha]_D^{21}$ = - 187,8 (c 1.0, CH_2Cl_2). IR: $\tilde{\nu}$ = 2849, 2820, 1731, 1715, 1476, 1437, 1237, 1198, 1164, 1019, 989, 754 cm^{-1}. HRMS (ESI) m/z $[M+H]^+$ Calcd. for $C_{21}H_{25}ClN_3O_3^+$: 402.1579; found: 402.1579.

Methyl 2-((6*S*,7*S*,8*S*)-8-(2,4-dichlorophenyl)-3-((*E*)-(2,2-dimethylhydrazono)methyl)-7-formyl-5,6,7,8-tetrahydroindolizin-6-yl)acetate (**4j**)

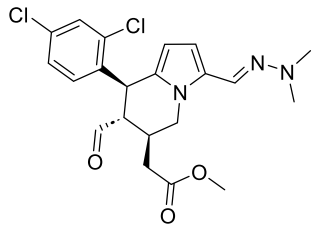

Following the general procedure, compound **4j** was isolated in 92% yield (40.0 mg) as a light-yellow oil. ^{1}H NMR (700 MHz, $CDCl_3$) δ 9.63 (d, J = 3.6 Hz, 1H), 7.40 (d, J = 2.1 Hz, 1H), 7.28 (s, 1H), 7.20 (dd, J = 8.4 Hz, J = 2.1 Hz, 1H), 7.17 (d, J = 8.3 Hz, 1H), 6.17 (d, J = 3.7 Hz, 1H), 5.48 (dd, J = 3.7 Hz, J = 1.3 Hz, 1H), 4.93 (d, J = 10.4 Hz, 1H), 4.82 (dd, J = 13.6 Hz, J = 5.0 Hz, 1H), 3.97 (dd, J = 13.6 Hz, J = 10.2 Hz, 1H), 3.69 (s, 3H), 2.95 (dtd, J = 12.2 Hz, J = 6.2 Hz, J = 5.5 Hz, J = 3.6 Hz,

1H), 2.88 – 2.85 (m, 1H), 2.84 (s, 6H), 2.52 (dd, *J* = 16.4 Hz, *J* = 5.8 Hz, 1H), 2.44 (dd, *J* = 16.4 Hz, *J* = 7.1 Hz, 1H). ^{13}C NMR (176 MHz, $CDCl_3$) *δ* 201.9, 171.8, 137.4, 134.6, 133.9, 131.6, 131.4, 129.8, 129.0, 128.9, 127.8, 111.5, 107.0, 56.7, 52.1, 48.7, 43.3 (2C), 38.8, 36.3, 32.4. The er was determined by UPC^2 using a chiral Chiralpack IA column gradient from 100% CO_2 up to 40%; *i*-PrOH, flow rate = 2.2 mL/min τ_{major} = 3.87 min, τ_{minor} = 3.62 min, (1:99 er). $[\alpha]_D^{21}$ = - 145,2 (*c* 1.0, CH_2Cl_2). IR: $\tilde{\nu}$ = 2952, 2848, 1724, 1471, 1438, 1196, 1166, 1017, 755 cm^{-1}. HRMS (ESI) m/z $[M+H]^+$ Calcd. for $C_{21}H_{24}Cl_2N_3O_3^+$: 436.1189; found: 436.1191.

Methyl 2-((6*S*,7*S*,8*S*)-3-((*E*)-(2,2-dimethylhydrazono)methyl)-7-formyl-8-(naphthalen-1-yl)-5,6,7,8-tetrahydroindolizin-6-yl)acetate (**4k**)

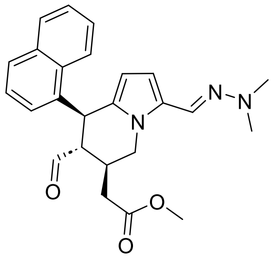

Following the general procedure, compound **4k** was isolated in 89% yield (37.1 mg) as a light-yellow oil. ^{1}H NMR (700 MHz, $CDCl_3$) *δ* 9.63 (d, *J* = 2.6 Hz, 1H), 7.83 – 7.78 (m, 3H), 7.72 (d, *J* = 1.7 Hz, 1H), 7.50 – 7.45 (m, 2H), 7.34 (dd, *J* = 8.4 Hz, *J* = 1.8 Hz, 1H), 7.31 (s, 1H), 6.17 (d, *J* = 3.7 Hz, 1H), 5.49 (dd, *J* = 3.8 Hz, *J* = 1.2 Hz, 1H), 4.83 (dd, *J* = 13.8 Hz, *J* = 4.1 Hz, 1H), 4.53 (d, *J* = 9.7 Hz, 1H), 4.04 (dd, *J* = 13.7 Hz, *J* = 9.6 Hz, 1H), 3.70 (s, 3H), 3.01 – 2.94 (m, 2H), 2.86 (s, 6H), 2.56 (dd, *J* = 16.2 Hz, *J* = 4.7 Hz, 1H), 2.46 (dd, *J* = 16.3 Hz, *J* = 6.2 Hz, 1H). ^{13}C NMR (176 MHz, $CDCl_3$) *δ* 203.1, 171.9, 138.0, 133.5 (2C), 133.0, 129.3, 128.9, 128.8, 127.9, 127.9, 127.8, 126.5, 126.4, 126.1, 111.4, 107.5, 56.8, 52.0, 49.0, 43.7, 43.4 (2C), 36.4, 32.7. The er was determined by UPC^2 using a chiral Chiralpack IC column gradient from 100% CO_2 up to 40%; *i*-PrOH, flow rate = 2.2 mL/min τ_{major} = 4.68 min, τ_{minor} = 4.43 min, (3:97 er). $[\alpha]_D^{21}$ = + 77,9 (*c* 1.0, CH_2Cl_2). IR: $\tilde{\nu}$ = 2920, 2850, 1719, 1159, 1012, 752, 734, 477 cm^{-1}. HRMS (ESI) m/z $[M+H]^+$ Calcd. for $C_{25}H_{28}N_3O_3^+$: 418.2125; found: 418.2123.

Methyl 2-((6*S*,7*S*,8*R*)-3-((*E*)-(2,2-dimethylhydrazono)methyl)-7-formyl-8-methyl-5,6,7,8-tetrahydroindolizin-6-yl)acetate (**4l**)

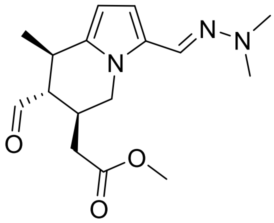

Following the modified general procedure (reaction performed with 5 equiv. of aldehyde at 0 °C for 48 h, then stirred overnight at room temperature), compound **4l** was isolated in 76% yield (23.3 mg) as a light-yellow oil. ^{1}H NMR (700 MHz, $CDCl_3$) *δ* 9.71 (d, *J* = 4.1 Hz, 1H), 7.28 (s, 1H), 6.22 (d, *J* = 3.7 Hz, 1H), 5.98 (dd, *J* = 3.7 Hz, *J* = 1.2 Hz, 1H), 4.65 (dd, *J* = 13.5 Hz, *J* = 5.0 Hz, 1H), 3.90 (dd, *J* = 13.5 Hz, *J* = 9.9 Hz, 1H), 3.68 (s, 3H), 3.25 – 3.20 (m, 1H), 2.81 (s, 6H), 2.79 (m, 1H), 2.40 (dd, *J* = 16.3 Hz, *J* = 6.1 Hz, 1H), 2.33 (dd, *J* = 16.3 Hz, *J* = 7.2 Hz, 1H), 2.16 (ddd, *J* = 10.6 Hz, *J* = 9.8 Hz, *J* = 4.1 Hz, 1H), 1.31 (d, *J* = 6.8 Hz, 3H). ^{13}C NMR (176 MHz, $CDCl_3$) *δ* 203.4, 172.0, 134.7, 129.5, 128.6, 111.4, 104.6, 57.8, 52.0, 48.4, 43.4 (2C), 36.7, 32.2, 29.7, 18.6. The er was determined by UPC^2 using a chiral Chiralpack IB column gradient from 100% CO_2 up to 40%; *i*-PrOH, flow rate = 2.2 mL/min τ_{major} = 2.82 min, τ_{minor} = 3.03 min, (99:1 er). $[\alpha]_D^{21}$ = - 6,0 (*c* 1.0, CH_2Cl_2). IR: $\tilde{\nu}$ = 2955, 2925, 1722, 1440, 1167, 1018, 986, 765 cm^{-1}. HRMS (ESI) m/z $[M+H]^+$ Calcd. for $C_{16}H_{24}N_3O_3^+$: 306.1812; found: 306.1810.

Methyl 2-((6*S*,7*S*,8*R*)-3-((*E*)-(2,2-dimethylhydrazono)methyl)-7-formyl-8-ethyl-5,6,7,8-tetrahydroindolizin-6-yl)acetate (**4m**)

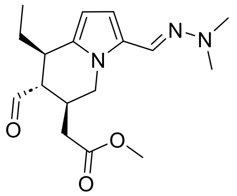

Following the modified general procedure (reaction performed at 0 °C overnight, then second equiv. of aldehyde was added, stirred at 0 °C overnight, then stirred overnight at room temperature), compound **4m** was isolated in 72% yield (22.9 mg) as a light-yellow oil. ^{1}H NMR (700 MHz, $CDCl_3$) δ 9.69 (d, J = 3.9 Hz, 1H), 7.29 (s, 1H), 6.23 (d, J = 3.7 Hz, 1H), 5.97 (dd, J = 3.7 Hz, J = 1.1 Hz, 1H), 4.65 (dd, J = 13.5 Hz, J = 4.8 Hz, 1H), 3.88 (dd, J = 13.5 Hz, J = 9.7 Hz, 1H), 3.68 (s, 3H), 3.30 – 3.25 (m, 1H), 2.83 – 2.79 (m, 6H), 2.79 – 2.74 (m, 1H), 2.43 – 2.31 (m, 3H), 1.83 (ddd, J = 14.4 Hz, J = 7.4 Hz, J = 5.1 Hz, 1H), 1.72 (ddd, J = 14.3 Hz, J = 7.3 Hz, J = 5.4 Hz, 1H), 0.91 (t, J = 7.4 Hz, 3H). ^{13}C NMR (176 MHz, $CDCl_3$) δ 203.1, 172.0, 132.9, 129.7, 128.5, 111.4, 105.2, 54.5, 52.0, 48.1, 43.4 (2C), 36.8, 35.3, 32.2, 25.9, 10.2. The er was determined by UPC^2 using a chiral Chiralpack IB column gradient from 100% CO_2 up to 40%; *i*-PrOH, flow rate = 2.2 mL/min τ_{major} = 2.78 min, τ_{minor} =2.94 min, (>99 er). $[\alpha]_D^{21}$ = - 6,0 (*c* 1.0, CH_2Cl_2). IR: $\tilde{\nu}$ = 2952, 2923, 1716, 1439, 1333, 1198, 1161, 1137, 1013, 768 cm^{-1}. HRMS (ESI) m/z $[M+H]^+$ Calcd. for $C_{17}H_{26}N_3O_3^+$: 320.1968; found: 320.1965.

Methyl 2-((6*S*,7*S*,8*R*)-3-((*E*)-(2,2-dimethylhydrazono)methyl)-7-formyl-8-((*Z*)-hex-3-en-1-yl)-5,6,7,8-tetrahydroindolizin-6-yl)acetate (**4n**)

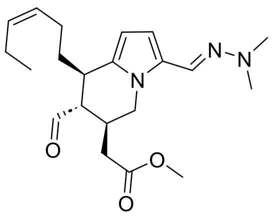

Following the modified general procedure (reaction performed at 0 °C overnight, then second equiv. of aldehyde was added, stirred at 0 °C overnight, then stirred overnight at room temperature), compound **4n** was isolated in 56% yield (20.9 mg) as a light-yellow oil. ^{1}H NMR (700 MHz, $CDCl_3$) δ 9.70 (d, J = 3.8 Hz, 1H), 7.29 (s, 1H), 6.23 (d, J = 3.7 Hz, 1H), 5.98 (dd, J = 3.7 Hz, J = 1.1 Hz, 1H), 5.41 – 5.35 (m, 1H), 5.29 (dtt, J = 10.3, J = 7.0 Hz, J = 1.5 Hz, 1H), 4.63 (dd, J = 13.5 Hz, J = 4.7 Hz, 1H), 3.93 (dd, J = 13.5 Hz, J = 9.5 Hz, 1H), 3.68 (s, 3H), 3.31 (dt, J = 10.1 Hz, J = 5.2 Hz, 1H), 2.82 (s, 6H), 2.80 – 2.74 (m, 1H), 2.41 – 2.31 (m, 3H), 2.11 – 2.06 (m, 2H), 1.99 (m, 2H), 1.76 (dtd, J = 8.6 Hz, J = 6.7 Hz, J = 5.4 Hz, 2H), 0.94 (t, J = 7.5 Hz, 3H). ^{13}C NMR (176 MHz, $CDCl_3$) δ 202.9, 172.0, 133.1 132.8, 129.7, 128.6, 128.1, 111.5, 105.2, 55.2, 52.0, 47.9, 43.4 (2C), 36.8, 33.9, 33.5, 32.3, 23.8, 20.7, 14.4. The er was determined by UPC^2 using a chiral Chiralpack IB column gradient from 100% CO_2 up to 40%; *i*-PrOH, flow rate = 2.2 mL/min τ_{major} = 2.82 min, τ_{minor} = 2.95 min, (>99 er). $[\alpha]_D^{21}$ = - 6,0 (*c* 1.0, CH_2Cl_2). IR: $\tilde{\nu}$ = 2956, 2867, 1724, 1438, 1166, 1018, 704 cm^{-1}. HRMS (ESI) m/z $[M+H]^+$ Calcd. for $C_{21}H_{32}N_3O_3^+$: 374.2438; found: 374.2434.

Methyl 2-((6*S*,7*S*,8*R*)-3-((*E*)-(2,2-dimethylhydrazono)methyl)-7-formyl-8-(phenethoxy-methyl)-5,6,7,8-tetrahydroindolizin-6-yl)acetate (**4o**)

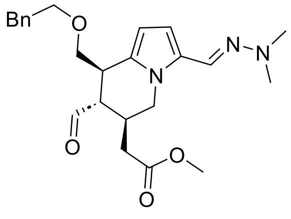

Following the modified general procedure (reaction performed at 0 °C overnight, then second equiv. of aldehyde was added, stirred at 0 °C overnight, then stirred overnight at room temperature), compound **4o** was isolated in 43% yield (17.7 mg) as a light-yellow oil. ^{1}H NMR (700 MHz, $CDCl_3$) δ 9.70 (d, J = 3.9 Hz, 1H), 7.36 – 7.33 (m, 2H), 7.28 (ddd, J = 5.8 Hz, J = 3.7 Hz, J = 2.2 Hz, 3H), 7.26 (s, 1H), 6.21 (d, J = 3.7 Hz, 1H), 5.95 (dd, J = 3.7 Hz, J = 1.2 Hz, 1H), 4.71 (dd, J = 13.4 Hz, J = 4.8 Hz, 1H), 4.53 – 4.48 (m, 2H), 3.90 (dd, J = 9.3 Hz, J = 4.5 Hz, 1H), 3.78 (dd, J = 13.4 Hz, J = 10.4 Hz, 1H), 3.68 (s, 3H), 3.66 – 3.61 (m, 1H), 3.45 (t, J = 9.2 Hz, 1H), 2.82 (s, 6H), 2.78 – 2.71 (m, 1H),

2.50 (dd, J = 16.3 Hz, J = 5.7 Hz, 1H), 2.43 (td, J = 10.0 Hz, J = 3.9 Hz, 1H), 2.33 (dd, J = 16.3 Hz, J = 7.3 Hz, 1H). ^{13}C NMR (176 MHz, $CDCl_3$) δ 202.6, 172.0, 137.8, 129.4, 129.1, 128.8, 128.6 (2C), 127.9 (3C), 111.3, 105.2, 73.5, 72.9, 54.6, 52.0, 48.8, 43.3 (2C), 36.5, 36.2, 31.9. The er was determined by UPC^2 using a chiral Chiralpack IA column gradient from 100% CO_2 up to 40%; *i*-PrOH, flow rate = 2.2 mL/min τ_{major} = 3.94 min, τ_{minor} = 3.84 min, (98:2 er). $[\alpha]_D^{21}$ = + 40,3 (*c* 1.0, CH_2Cl_2). IR: $\tilde{\nu}$ = 2950, 2855, 1720, 1687, 1657, 1094, 1072, 1021, 771, 736, 698 cm^{-1}. HRMS (ESI) m/z $[M+H]^+$ Calcd. for $C_{23}H_{30}N_3O_4^+$: 412.2231; found: 412.2222.

Methyl 2-((6*S*,7*S*,8*S*)-7-formyl-3-((*E*)-(morpholinoimino)methyl)-8-phenyl-5,6,7,8-tetrahydroindolizin-6-yl)acetate (**4p**)

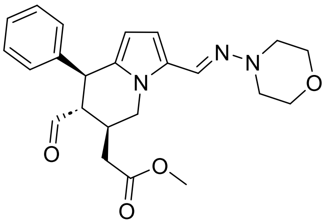

Following the general procedure, compound **4p** was isolated in 86% yield (35.2 mg) as a yellow solid. Mp = 123-124 °C. ^{1}H NMR (700 MHz, $CDCl_3$) δ 9.61 (d, J = 3.2 Hz, 1H), 7.61 (s, 1H), 7.32 (t, J = 7.5 Hz, 2H), 7.30 – 7.26 (m, 1H), 7.27 – 7.22 (m, 2H), 6.23 (d, J = 3.7 Hz, 1H), 5.51 (dd, J = 3.7 Hz, J = 1.1 Hz, 1H), 4.80 (dd, J = 13.6 Hz, J = 5.1 Hz, 1H), 4.36 (d, J = 10.8 Hz, 1H), 4.04 (dd, J = 13.6 Hz, J = 10.1 Hz, 1H), 3.90 – 3.86 (m, 4H), 3.69 (s, 3H), 3.08 – 3.03 (m, 4H), 2.94 (dtd, J = 12.0 Hz, J = 10.3 Hz, J = 5.7 Hz, 1H), 2.87 (td, J = 10.4 Hz, J = 3.2 Hz, 1H), 2.53 (dd, J = 16.4 Hz, J = 5.7 Hz, 1H), 2.43 (dd, J = 16.3 Hz, J = 6.9 Hz, 1H). ^{13}C NMR (176 MHz, $CDCl_3$) δ 202.8, 171.7, 140.3, 134.7, 132.5, 128.8 (2C), 128.7 (2C), 127.7, 127.6, 113.1, 107.4, 66.5 (2C), 56.8, 52.5 (2C), 51.9, 48.9, 43.3, 36.2, 32.4. The er was determined by HPLC using a chiral Chiralpack IB column [hexane:*i*-PrOH, 80:20]; flow rate 1.0 mL/min; τ_{major}=13.27 min; τ_{minor}=19.46 min, (95:5 er). $[\alpha]_D^{19}$ = +10.7 (*c* 1.0, CH_2Cl_2). IR: $\tilde{\nu}$ = 2820, 2726, 1724, 1114, 1090, 996, 906, 757, 729, 701 cm^{-1}. HRMS (ESI) m/z $[M+H]^+$ Calcd. for $C_{23}H_{28}N_3O_4^+$: 410.2074; found: 410.2075.

4. Synthesis of methyl 2-((6*S*,7*S*,8*S*)-3-((*E*)-(2,2-dimethylhydrazono)methyl)-7-formyl-8-phenyl-5,6,7,8-tetrahydroindolizin-6-yl)acetate (4a) – 1 mmol scale experiment

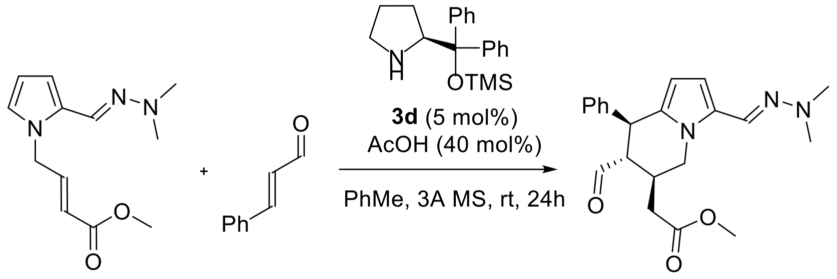

Scheme S4.

To a stirred solution of the corresponding (*E*)-methyl 4-(2-((*E*)-(2,2-dimethylhydrazono)methyl)-1*H*-pyrrol-1-yl)but-2-enoate **1a** (1.2 equiv. 1.2 mmol, 141 mg) cinnamaldehyde **2a** (1.0 equiv., 1.0 mmol, 132 mg) in toluene (4 mL), (*S*)-(–)-*α,α*-diphenyl-2-pyrrolidinemethanol trimethylsilyl ether (**3d**) (5 mol%, 0.05 mmol, 16.3 mg) and acetic acid (40 mol%, 0.2 mmol, 24 μL) were added and resulting mixture was stirred overnight. After evaporation of solvents the reaction mixture was purified by column chromatography (petroleum ether/ethyl acetate 8:1) to give product **4a** in 77% yield. The er was determined by UPC^2 using a chiral Chiralpack IA column gradient from 100% CO_2 up to 40%; *i*-PrOH, flow rate = 2.2 mL/min τ_{major} = 3.53 min, τ_{minor} = 3.59 min, (95:5 er). NMR data were in accordance with previously obtained results.

5. Synthesis of methyl 2-((6*S*,7*S*,8*S*)-3-cyano-7-formyl-8-phenyl-5,6,7,8-tetrahydroindo-lizin-6-yl)acetate (6a)

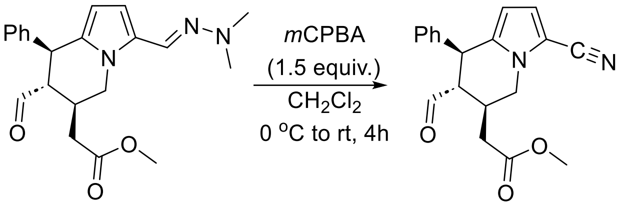

4a **6a**

Scheme S5.

Reaction was carried out following the modified literature procedure.[2] To a stirred solution of methyl 2-((6*S*,7*S*,8*S*)-3-((*E*)-(2,2-dimethylhydrazono)methyl)-7-formyl-8-phenyl-5,6,7,8-tetrahydroindolizin-6-yl)acetate (**4a**) (1.0 equiv., 0.5 mmol, 183.5 mg) in CH_2Cl_2 (5 mL), *m*CBPA (1.5 equiv., 0.75 mmol, 170 mg, 77% *m*CPBA was used) in CH_2Cl_2 (15 mL) was added dropwise at 0 °C. Then the reaction mixture was stirred at room temperature. After full consumption of starting material **4a** (as indicated by TLC analysis, about 4 h) K_2CO_3 (5.0 equiv., 0.25 mmol, 345 mg) was added in one portion and reaction mixture was stirred for a few minutes in an open flask. The reaction mixture was filtered and solid was washed with CH_2Cl_2 (25 mL). After evaporation of the solvent crude product was purified by flash column chromatography (petroleum ether/ethyl acetate 8:1) to give nitrile **6a** in 95% yield (153.0 mg) as a white solid. ^{1}H NMR (700 MHz, $CDCl_3$) δ 9.60 (d, J = 2.5 Hz, 1H), 7.35 – 7.32 (m, 2H), 7.31 – 7.28 (m, 1H), 7.21 – 7.19 (m, 2H), 6.73 (d, J = 4.0 Hz, 1H), 5.57 (dd, J = 4.0 Hz, J = 1.1 Hz, 1H), 4.41 – 4.37 (m, 1H), 4.31 (d, J = 9.9 Hz, 1H), 3.90 (dd, J = 13.1 Hz, J = 10.0 Hz, 1H), 3.71 (s, 3H), 3.00 – 2.96 (m, 2H), 2.57 (dd, J = 16.8 Hz, J = 4.4 Hz, 1H), 2.50 – 2.45 (m, 1H). ^{13}C NMR (176 MHz, $CDCl_3$) δ 202.0, 171.3, 139.3, 137.2, 129.2 (2C), 128.7 (2C), 128.2, 120.2, 113.8, 108.5, 102.4, 56.3, 52.3, 47.8, 43.4, 35.3, 32.1. $[\alpha]_D^{21}$ = - 18,3 (*c* 1.0, CH_2Cl_2). IR: $\tilde{\nu}$ = 2953, 2209, 1723, 1434, 1324, 1198, 1166, 764, 745, 700 cm^{-1}. HRMS (ESI) m/z $[M+H]^+$ Calcd. for $C_{19}H_{19}N_2O_3^+$: 323.1390; found: 323.1383.

---

[2] Młochowski, J.; Kloc K.; Kubicz, E. *J. Prakt. Chem.* **1994**, *336*, 467-469.

6. Synthesis of methyl 2-((6*S*,7*S*,8*S*)-3-cyano-7-((phenylamino)methyl)-8-phenyl-5,6,7,8-tetrahydroindolizin-6-yl)acetate (7a)

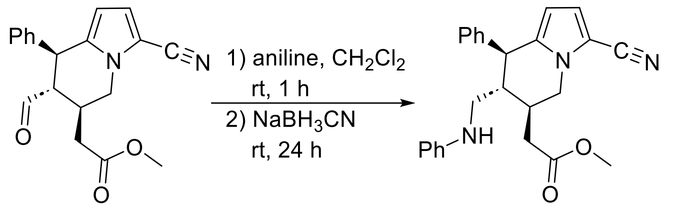

Scheme S6.

To a stirred solution of methyl 2-((6*S*,7*S*,8*S*)-3-cyano-7-formyl-8-phenyl-5,6,7,8-tetrahydroindolizin-6-yl)acetate (**6a**) (1.0 equiv., 0.1 mmol, 32.2 mg,) in CH_2Cl_2 (1 mL), aniline (1.1 equiv., 0.11 mmol, 10 μL) was added at room temperature. The resulting reaction mixture was stirred for 1 hour, then $NaBH_3CN$ was added (5.0 equiv., 0.5 mmol, 26 μL) and the mixture was stirred overnight. The reaction mixture was filtered and solid was washed with CH_2Cl_2 (25 mL). The reaction mixture was extracted with $NaHCO_3$ and washed three times with CH_2Cl_2. Combined organic layers were dried over anhydrous Na_2SO_4, filtered and concentrated in vacuo. Crude product was purified by flash column chromatography (petroleum ether/ethyl acetate 8:1) to give **7a** in 52% yield (20.7 mg) as a light-yellow oil. ^{1}H NMR (700 MHz, $CDCl_3$) δ 7.36 – 7.29 (m, 3H), 7.20 – 7.17 (m, 2H), 7.14 – 7.09 (m, 2H), 6.71 – 6.68 (m, 2H), 6.46 – 6.41 (m, 2H), 5.50 (dd, J = 4.0 Hz, J = 1.1 Hz, 1H), 4.32 (dd, J = 12.6 Hz, J = 4.8 Hz, 1H), 3.99 (d, J = 10.5 Hz, 1H), 3.95 (dd, J = 12.6 Hz, J = 10.0 Hz, 1H), 3.79 – 3.68 (m, 1H), 3.64 (s, 3H), 3.11 (d, J = 4.3 Hz, 2H), 2.78 – 2.72 (m, 1H), 2.61 (dd, J = 16.7 Hz, J = 6.0 Hz, 1H), 2.46 (dd, J = 16.7 Hz, J = 6.2 Hz, 1H), 2.16 (ddt, J = 10.5 Hz, J = 9.2 Hz, J = 4.5 Hz, 1H). ^{13}C NMR (176 MHz, $CDCl_3$) δ 172.3, 147.9, 141.3, 139.2, 129.3 (2C), 129.1 (2C), 129.0 (2C), 127.7, 120.0, 118.0, 114.1, 113.3 (2C), 108.5, 101.9, 52.2, 48.4, 45.1, 44.4, 43.6, 36.0, 34.2. $[\alpha]_D^{21}$ = + 45,6 (*c* 1.0, CH_2Cl_2). IR: $\tilde{\nu}$ = 3391, 2922, 2852, 2207, 1727, 1601, 1432, 1168, 747, 695 cm^{-1}. HRMS (ESI) m/z $[M+H]^+$ Calcd. for $C_{25}H_{26}N_3O_2^+$: 400.2019; found: 400.2021.

7. Synthesis of (4a*S*,10*S*,10a*S*)-3-oxo-2,10-diphenyl-1,2,3,4,4a,5,10,10a-octahydro-pyrrolo[1,2-*b*][2,6]naphthyridine-7-carbonitrile (8a)

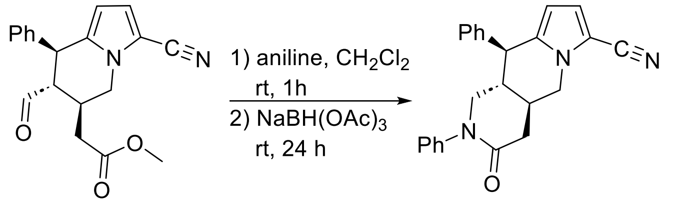

6a **8a**

Scheme S7.

To a stirred solution of methyl 2-((6*S*,7*S*,8*S*)-3-cyano-7-formyl-8-phenyl-5,6,7,8-tetrahydroindolizin-6-yl)acetate (**6a**) (1.0 equiv., 0.1 mmol, 32.2 mg,) in CH_2Cl_2 (1 mL), aniline (1.1 equiv., 0.11 mmol, 10 µL) was added at room temperature. The resulting reaction mixture was stirred for 1 hour, then $NaBH(OAc)_3$ was added (5.0 equiv., 0.5 mmol, 53 mg) and the mixture was stirred overnight. The reaction mixture was filtered and solid was washed with CH_2Cl_2 (25 mL). The reaction mixture was extracted with $NaHCO_3$ and washed three times with CH_2Cl_2. Combined organic layers were dried over anhydrous Na_2SO_4, filtered and concentrated in vacuo. Crude product was purified by flash column chromatography (petroleum ether/ethyl acetate 8:1) to give **8a** in 45% yield (16.5 mg) as a light-yellow oil. ^{1}H NMR (700 MHz, $CDCl_3$) δ 7.38 – 7.32 (m, 4H), 7.32 – 7.28 (m, 1H), 7.26 – 7.22 (m, 1H), 7.21 – 7.14 (m, 4H), 6.73 (d, *J* = 4.0 Hz, 1H), 5.50 (dd, *J* = 4.0 Hz, *J* = 1.1 Hz, 1H), 4.47 (dd, *J* = 12.7 Hz, *J* = 5.5 Hz, 1H), 3.79 – 3.71 (m, 2H), 3.58 (dd, *J* = 12.4 Hz, *J* = 10.8 Hz, 1H), 3.41 (dd, *J* = 12.4 Hz, *J* = 5.1 Hz, 1H), 2.93 (dd, *J* = 17.6 Hz, *J* = 5.8 Hz, 1H), 2.65 (qt, *J* = 11.4 Hz, *J* = 5.6 Hz, 1H), 2.46 (dd, *J* = 17.6 Hz, *J* = 11.7 Hz, 1H), 2.38 (dtd, *J* = 11.9 Hz, *J* = 10.8 Hz, *J* = 5.1 Hz, 1H). ^{13}C NMR (176 MHz, $CDCl_3$) δ 167.4, 142.5, 139.7, 138.0, 129.4 (2C), 129.2 (2C), 128.6 (2C), 128.1, 127.2, 126.1 (2C), 120.2, 113.9, 108.6, 102.3, 54.9, 49.0, 46.6, 40.1, 36.0, 33.9. $[\alpha]_D^{21}$ = + 106,7 (*c* 1.0, CH_2Cl_2). IR: $\tilde{\nu}$ = 3101, 2915, 2857, 2205, 1642, 1493, 1432, 1323, 1276, 729, 697, 596, 539 cm^{-1}. HRMS (ESI) m/z $[M+H]^+$ Calcd. for $C_{24}H_{22}N_3O^+$: 368.1757; found: 368.1754.

8. Synthesis of (4a*S*,10*S*,10a*S*)-3-oxo-10-phenyl-3,4,4a,5,10,10a-hexahydro-1*H*-pyrano-[4,3-*f*]indolizine-7-carbonitrile (9a)

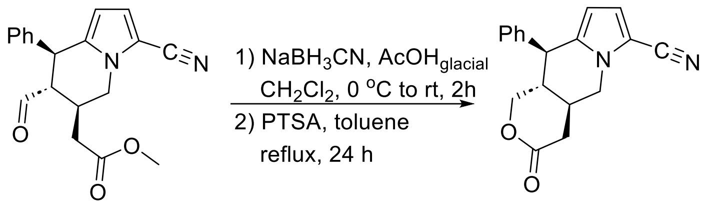

Scheme S8.

To a solution of methyl 2-((6*S*,7*S*,8*S*)-3-cyano-7-formyl-8-phenyl-5,6,7,8-tetrahydroindolizin-6-yl)acetate (**6a**) (1.0 equiv., 0.115 mmol, 37.3 mg) in CH_2Cl_2 (0.925 mL) $NaBH_3CN$ (2.0 equiv., 0.23 mmol, 46 µL) and glacial acetic acid (0.23 mL) were added at 0 °C. The reaction mixture was stirred at this temperature until full consumption of starting material **6a** (as indicated by TLC analysis, about 2 h). The reaction mixture was extracted with H_2O and washed three times with CH_2Cl_2. Combined organic layers were dried over anhydrous Na_2SO_4 and filtered. The concentrated residue was dissolved in toluene (1.15 mL), PTSA was added (0.2 equiv., 0.023 mmol, 3.96 mg) and the resulting reaction mixture was refluxed overnight. After cooling down to room temperature the reaction mixture was extracted with $NaHCO_3$ and washed three times with CH_2Cl_2. Combined organic layers were dried over anhydrous Na_2SO_4, filtered and concentrated in vacuo. Crude product was purified by flash column chromatography (petroleum ether/ethyl acetate 8:1) to give **9a** in 57% yield (19.2 mg) as a light-yellow oil. ^1H NMR (700 MHz, $CDCl_3$) δ 7.40 – 7.32 (m, 3H), 7.20 – 7.16 (m, 2H), 6.73 (d, J = 4.0 Hz, 1H), 5.54 (dd, J = 4.0 Hz, J = 1.2 Hz, 1H), 4.44 (dd, J = 12.5 Hz, J = 5.4 Hz, 1H), 4.22 (dd, J = 11.6 Hz, J = 4.7 Hz, 1H), 4.09 (t, J = 11.4 Hz, 1H), 3.73 (t, J = 11.9 Hz, 1H), 3.65 (d, J = 11.0 Hz, 1H), 3.00 (dd, J = 18.1 Hz, J = 6.3 Hz, 1H), 2.58 (tq, J = 11.5 Hz, J = 6.0 Hz, 1H), 2.40 (dd, J = 18.1 Hz, J = 11.4 Hz, 1H), 2.25 (qd, J = 11.3 Hz, J = 4.7 Hz, 1H). ^{13}C NMR (176 MHz, $CDCl_3$) δ 168.1, 139.1, 137.6, 129.3 (2C), 128.5 (2C), 128.4, 120.3, 113.7, 108.8, 102.5, 72.0, 49.0, 44.8, 39.5, 33.31, 33.28. $[\alpha]_D^{21}$ = + 44,9 (*c* , CH_2Cl_2). IR: $\tilde{\nu}$ = 2956, 2918, 2200, 1736, 1495, 1208, 1058, 772, 739, 701 cm^{-1}. HRMS (ESI) m/z $[M+H]^+$ Calcd. for $C_{18}H_{17}N_2O_2^+$: 293.1284; found: 293.1286.

9. Crystal and X-ray data for methyl 2-((6*S*,7*S*,8*S*)-3-((*E*)-(2,2-dimethylhydrazono)-methyl)-7-formyl-8-(4-nitrophenyl)-5,6,7,8-tetrahydroindolizin-6-yl)acetate (4g)

Suitable crystals of compound **4g** were obtained by slowly evaporating a mixture of cyclohexane and ethyl acetate solution at room temperature. The crystal structure of the compound **4g**, $C_{21}H_{24}N_4O_5$, was established by single-crystal X-ray diffraction at 100 K. The compound crystallizes in the non-centrosymmetric monoclinic space group $P2_1$ (Z = 4), with two crystallographically independent formula units per unit cell (Figure S1).

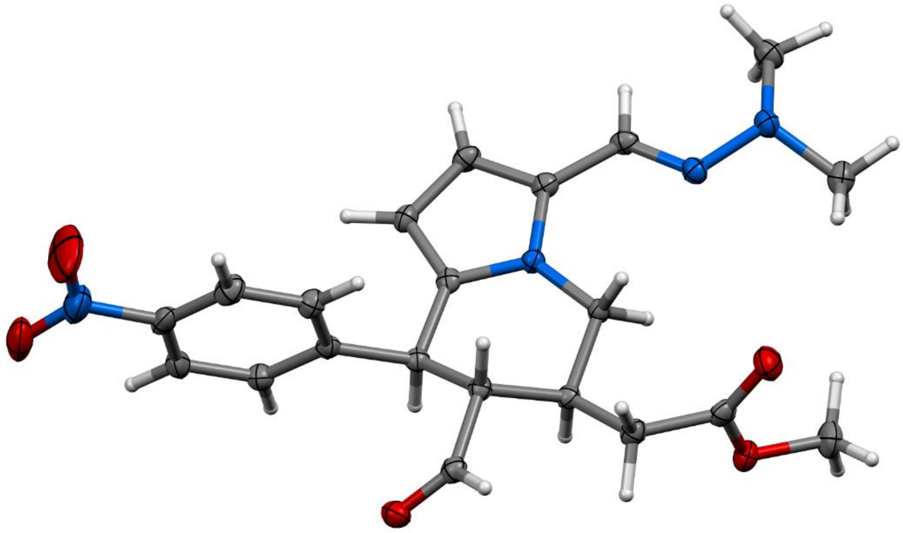

Figure S1. The molecular structure of the compound **49** at 100 K (one of two independent molecules), showing 50% probability displacement ellipsoids. Hydrogen atoms are drawn with an arbitrary radius.

Single crystal X-ray diffraction data were collected at 100 K by the ω-scan technique using a RIGAKU XtaLAB Synergy, Dualflex, Pilatus 300K diffractometer[3] with PhotonJet micro-focus X-ray Source Cu-Kα (λ = 1.54184 Å). Data collection, cell refinement, data reduction and absorption correction were performed using CrysAlis PRO software.[4] The crystal structure was solved using direct methods and the SHELXT 2018/2 program,[5] with atomic scattering factors taken from the International Tables for X-ray Crystallography. Positional parameters of non-H-atoms were refined by a full-matrix least-squares method on F^2 with anisotropic thermal parameters by using the SHELXL 2019/3 program.6 All hydrogen atoms were positioned geometrically in calculated positions (C–H = 0.95–1.00 Å) and constrained to ride on their parent atoms with isotropic displacement parameters set to 1.2 times the U_{eq} of the parent atom. In one of two molecules the ester group was refined as disordered over two sets of sites with an occupancy ratio of 0.829(5): 0.171(5).

[3] Rigaku, O.D. CrysAlis PRO. Rigaku Oxford Diffraction Ltd, Yarnton, Oxfordshire, England, **2019**

[4] Sheldrick, G.M. SHELXT - integrated space-group and crystal-structure determination, *Acta Cryst.* **2015**, *A71*, 3-8.

[5] Sheldrick, G.M. Crystal structure refinement with SHELXL, *Acta Cryst.* **2015**, *C71*, 3-8.

[6] Parsons, S.; Flack, H.D.; Wagner, T. Use of intensity quotients and differences in absolute structure refinement, *Acta Cryst.* **2013**, *B69*, 249-259.

4g: Formula $C_{21}H_{24}N_4O_5$, monoclinic, space group *P*2$_1$, Z = 4, unit cell constants *a* = 12.8583(1), *b* = 5.5296(1), *c* = 28.6721(2) Å, β = 96.469(1)°, *V* = 2025.64(4) Å^3. The integration of the data yielded a total of 55118 reflections with θ angles in the range of 3.46 to 67.72°, of which 7189 were unique (R_{int} = 2.6%). The final anisotropic full-matrix least-squares refinement on F^2 with 576 parameters. The final R_1 was 0.0263 (for I > 2σ(I)) and wR_2 was 0.0713 (all data). The largest peak in the final difference electron density synthesis was 0.420 eÅ^{-3} and the largest hole was -0.323 eÅ^{-3}. The goodness-of-fit was 1.033. The absolute configuration was unambiguously established from anomalous scattering, by calculating the x Flack parameter[6] of -0.07(3) using 2998 quotients.

CCDC 2284783 contains the supplementary crystallographic data for this paper. These data can be obtained free of charge from The Cambridge Crystallographic Data Centre via www.ccdc.cam.ac.uk/structures.

10. NMR data

(*E*)-Methyl 4-(2-((*E*)-(2,2-dimethylhydrazono)methyl)-1*H*-pyrrol-1-yl)but-2-enoate (**1a**)

^{1}H NMR (700 MHz, $CDCl_3$)

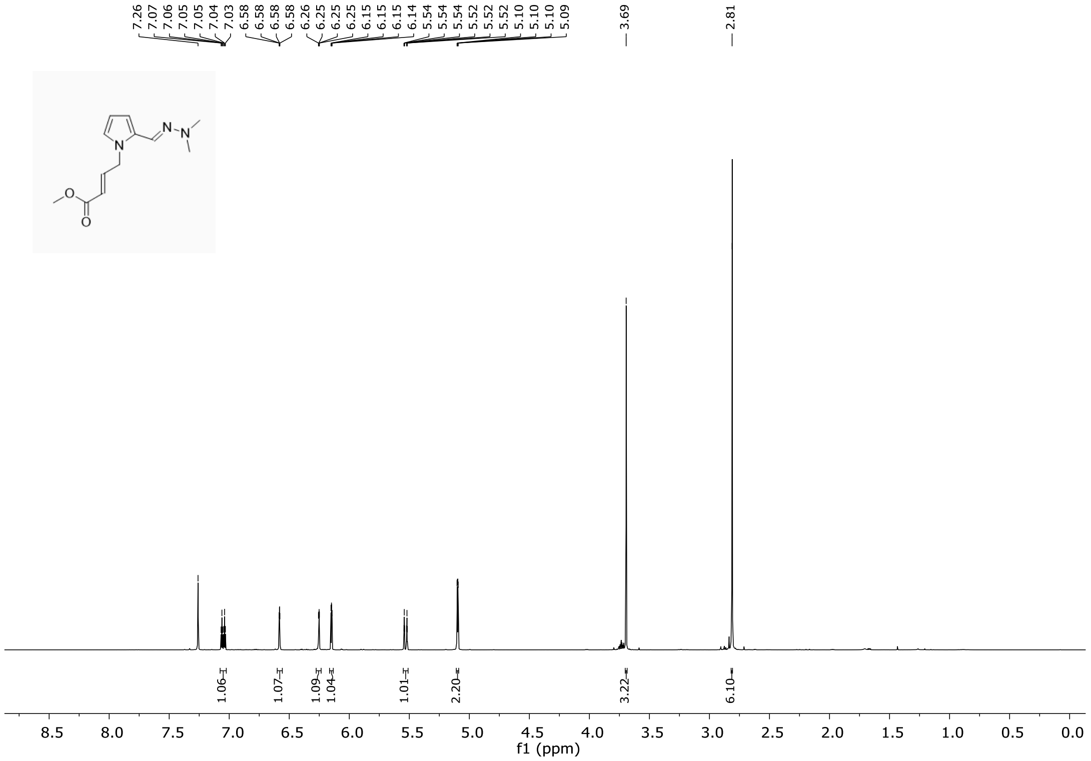

^{13}C NMR (176 MHz, $CDCl_3$)

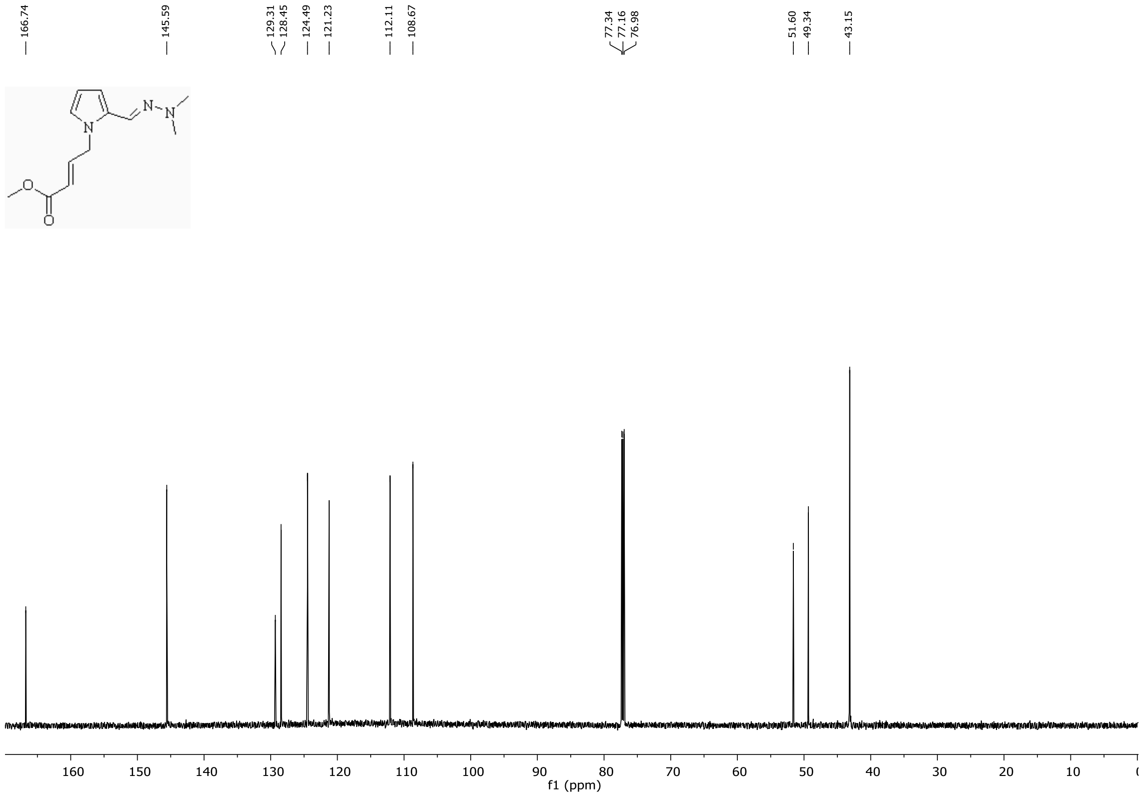

(*E*)-methyl 4-(2-((*E*)-(morpholinoimino)methyl)-1*H*-pyrrol-1-yl)but-2-enoate **1b**

^{1}H NMR (700 MHz, $CDCl_3$)

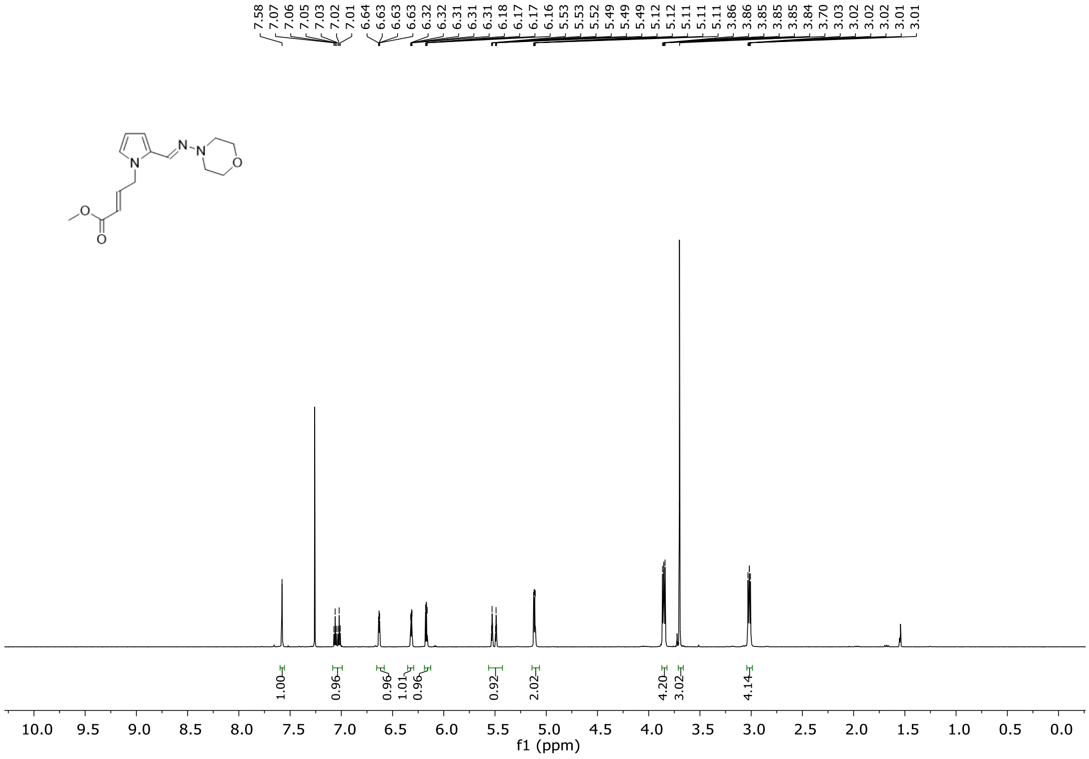

^{13}C NMR (176 MHz, $CDCl_3$)

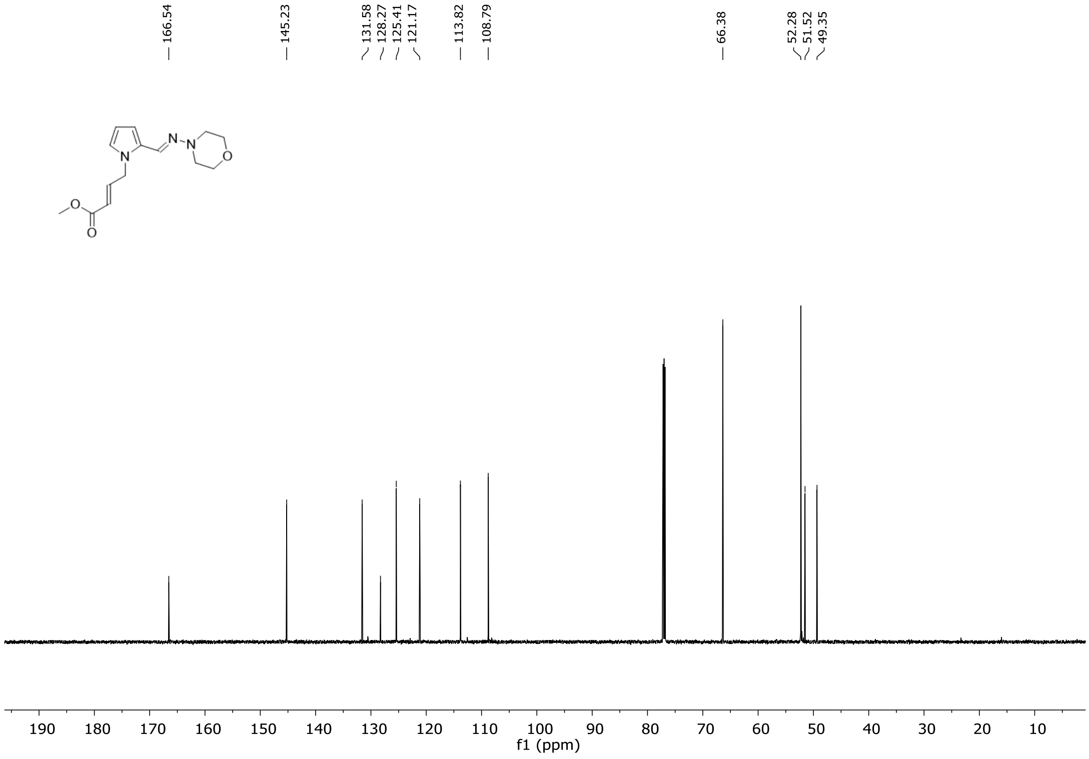

Methyl 2-((6*S*,7*S*,8*S*)-3-((*E*)-(2,2-dimethylhydrazono)methyl)-7-formyl-8-phenyl-5,6,7,8-tetrahydroindolizin-6-yl)acetate (**4a**)

^{1}H NMR (700 MHz, $CDCl_3$)

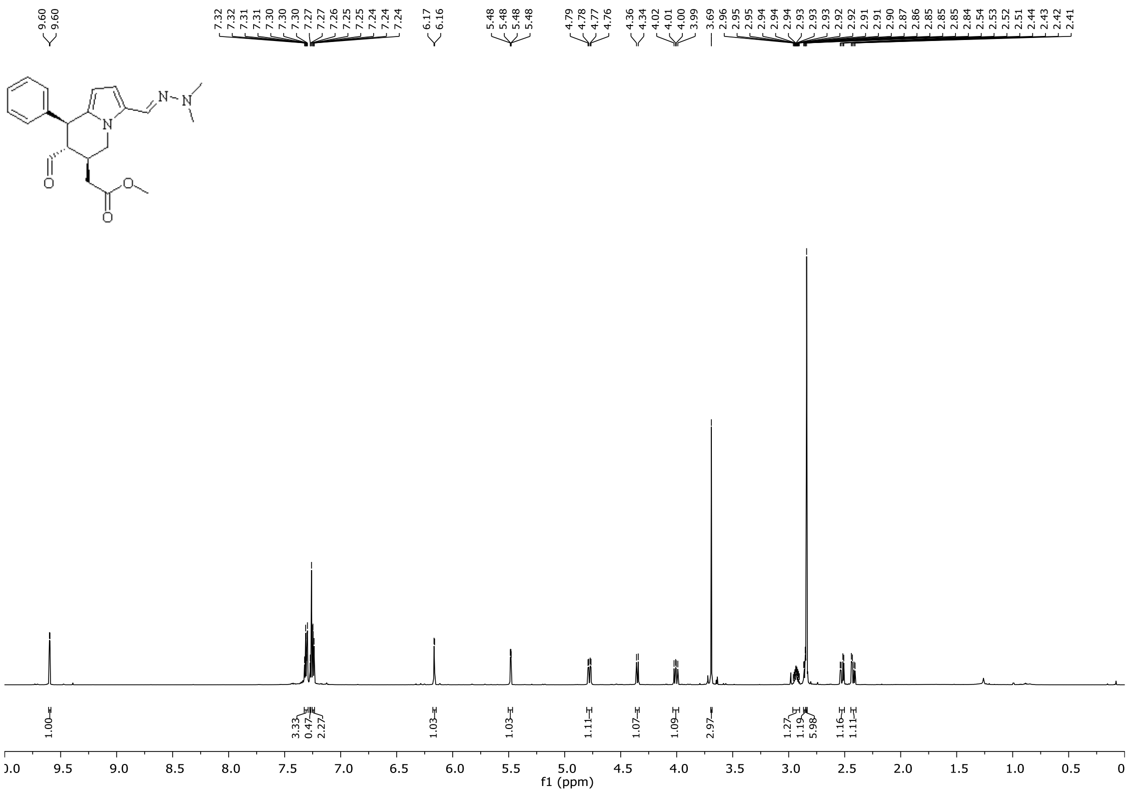

^{13}C NMR (176 MHz, $CDCl_3$)

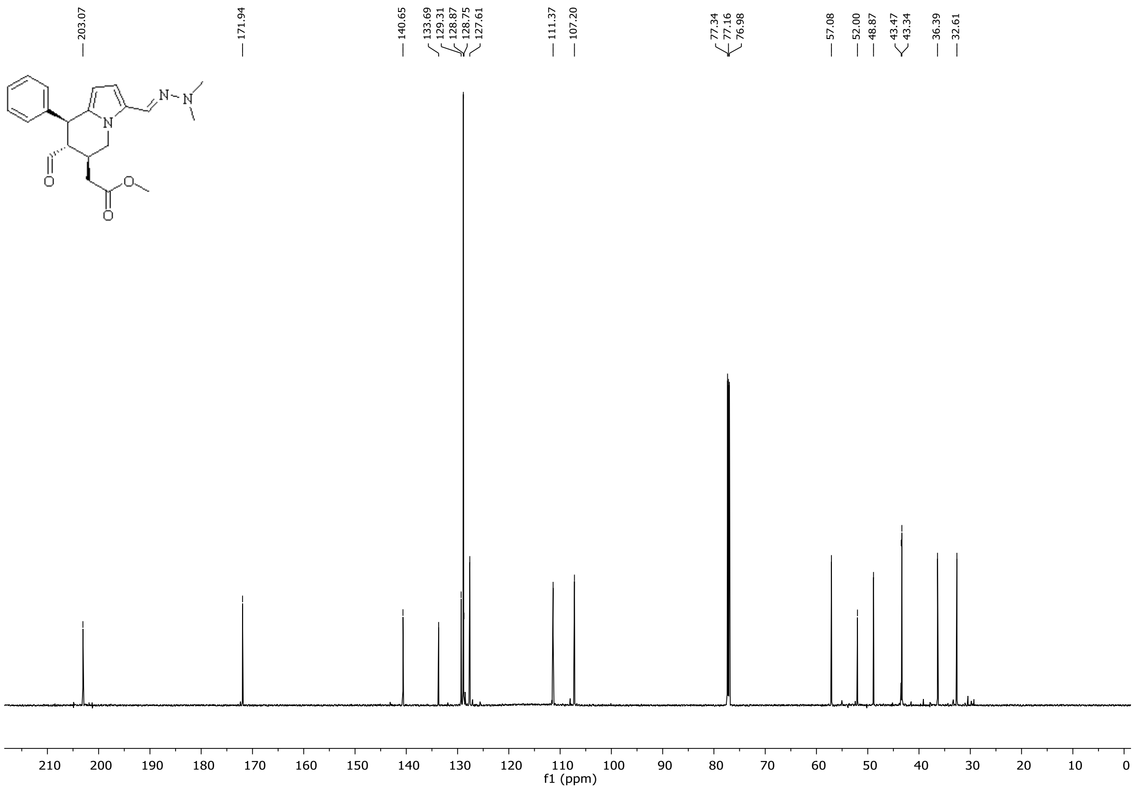

Methyl 2-((6*S*,7*S*,8*S*)-3-((*E*)-(2,2-dimethylhydrazono)methyl)-7-formyl-8-(4-methoxyphenyl)-5,6,7,8-tetrahydroindolizin-6-yl)acetate (**4b**)

^{1}H NMR (700 MHz, $CDCl_3$)

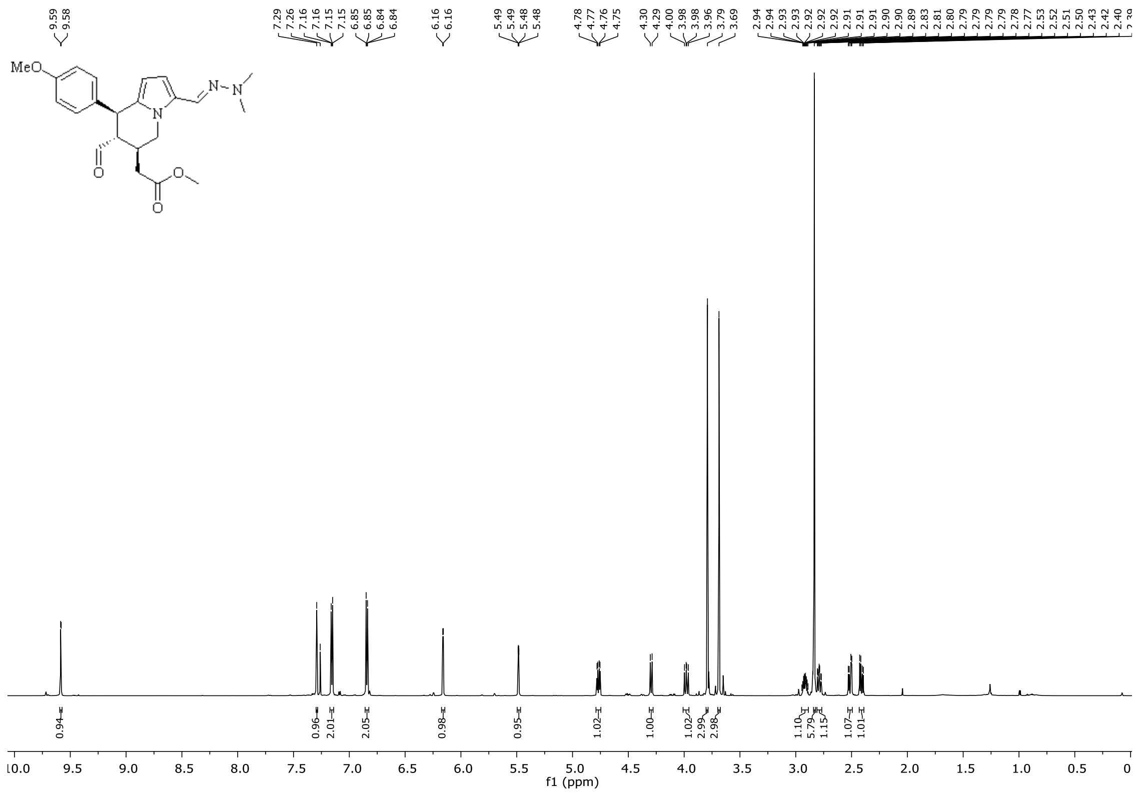

^{13}C NMR (176 MHz, $CDCl_3$)

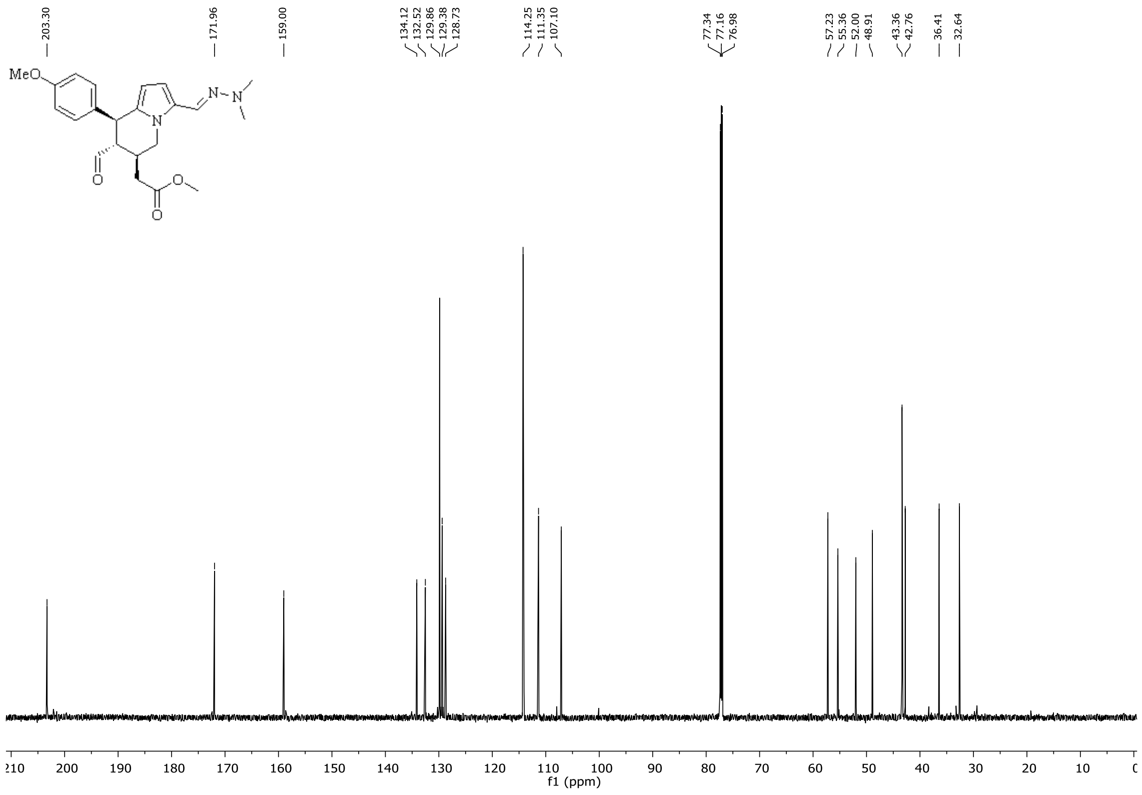

Methyl 2-((6*S*,7*S*,8*S*)-3-((*E*)-(2,2-dimethylhydrazono)methyl)-7-formyl-8-(3-methoxyphenyl)-5,6,7,8-tetrahydroindolizin-6-yl)acetate (**4c**)

^{1}H NMR (700 MHz, CDCl$_3$)

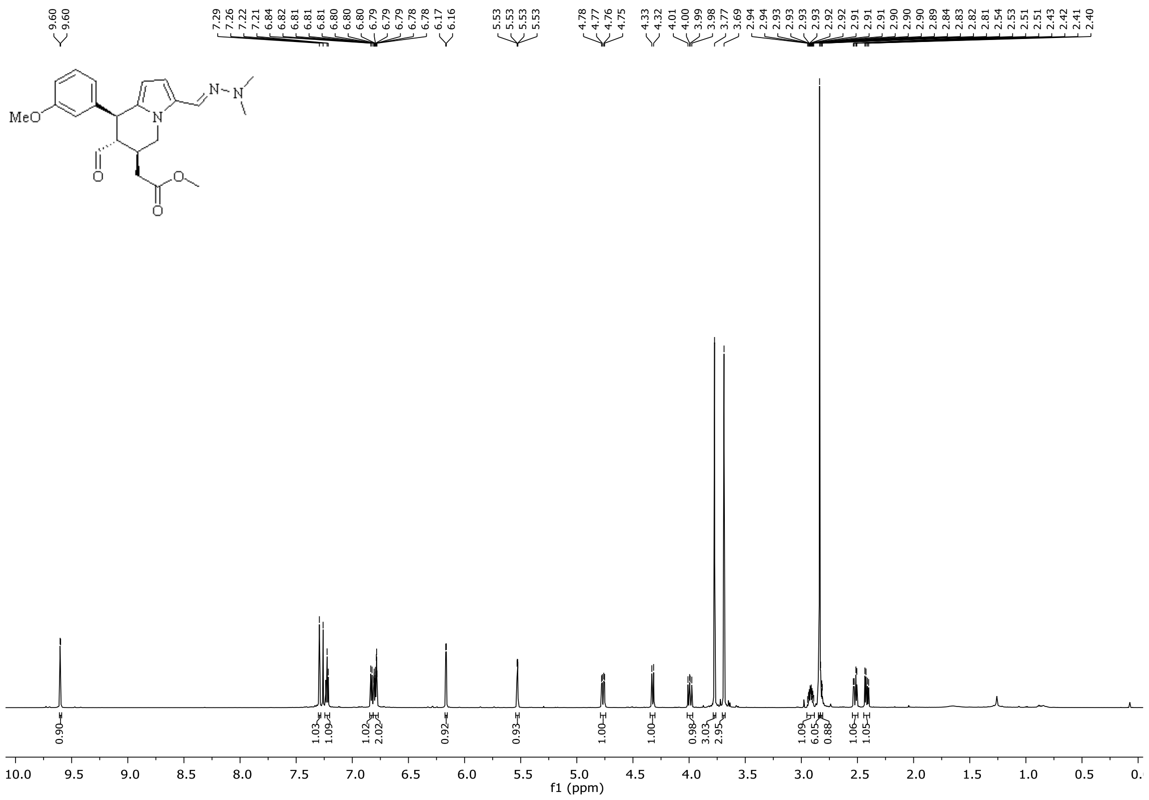

^{13}C NMR (176 MHz, CDCl$_3$)

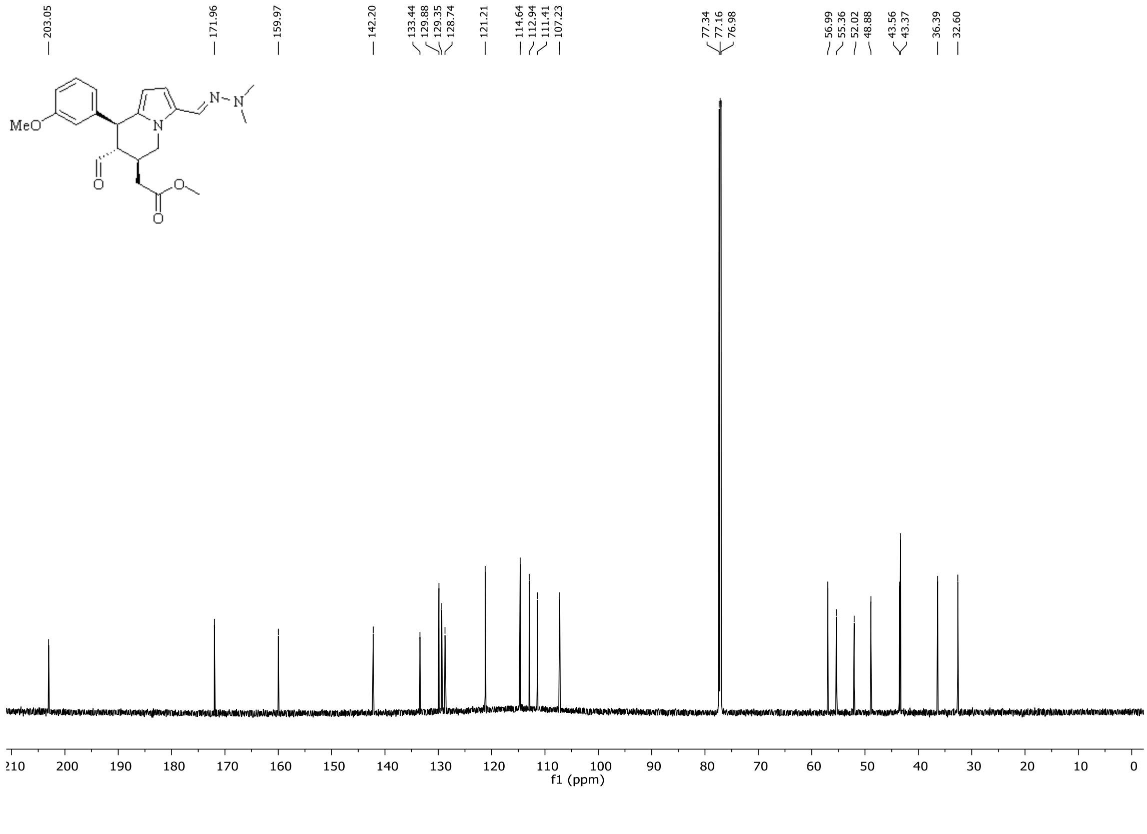

Methyl 2-((6*S*,7*S*,8*S*)-3-((*E*)-(2,2-dimethylhydrazono)methyl)-7-formyl-8-(2-methoxyphenyl)-5,6,7,8-tetrahydroindolizin-6-yl)acetate (**4d**)

^{1}H NMR (400 MHz, $CDCl_3$)

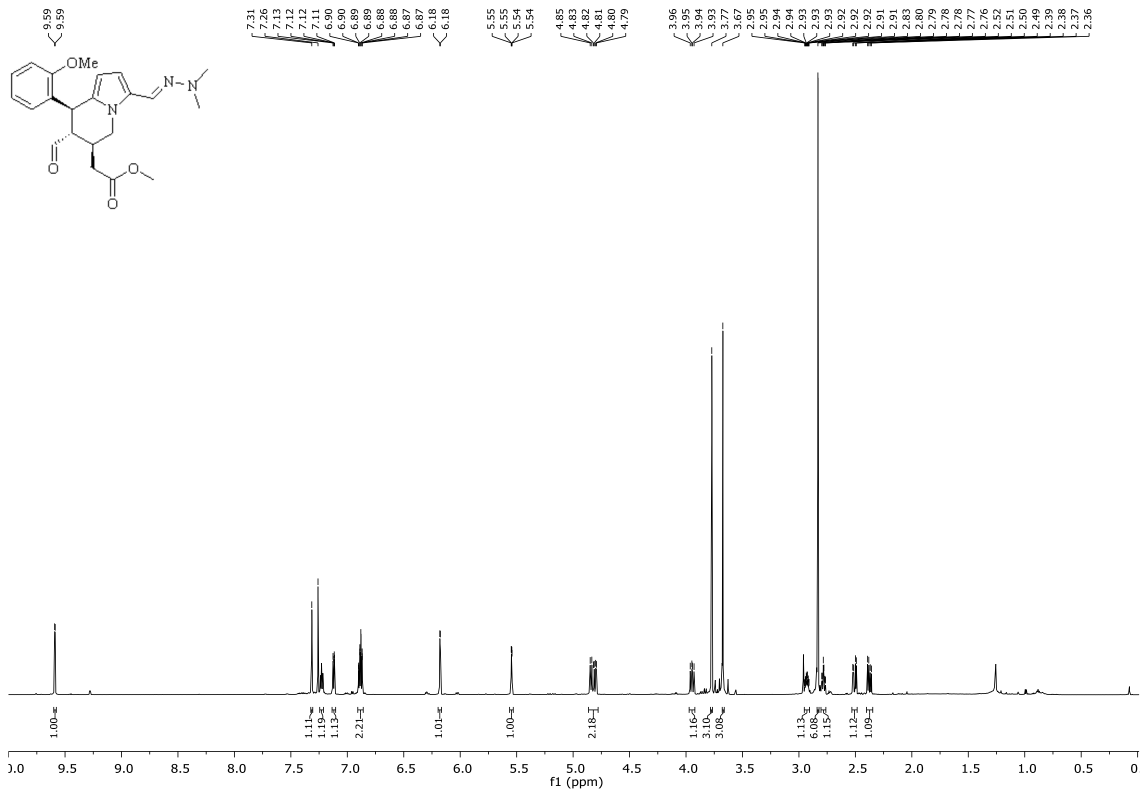

^{13}C NMR (101 MHz, $CDCl_3$)

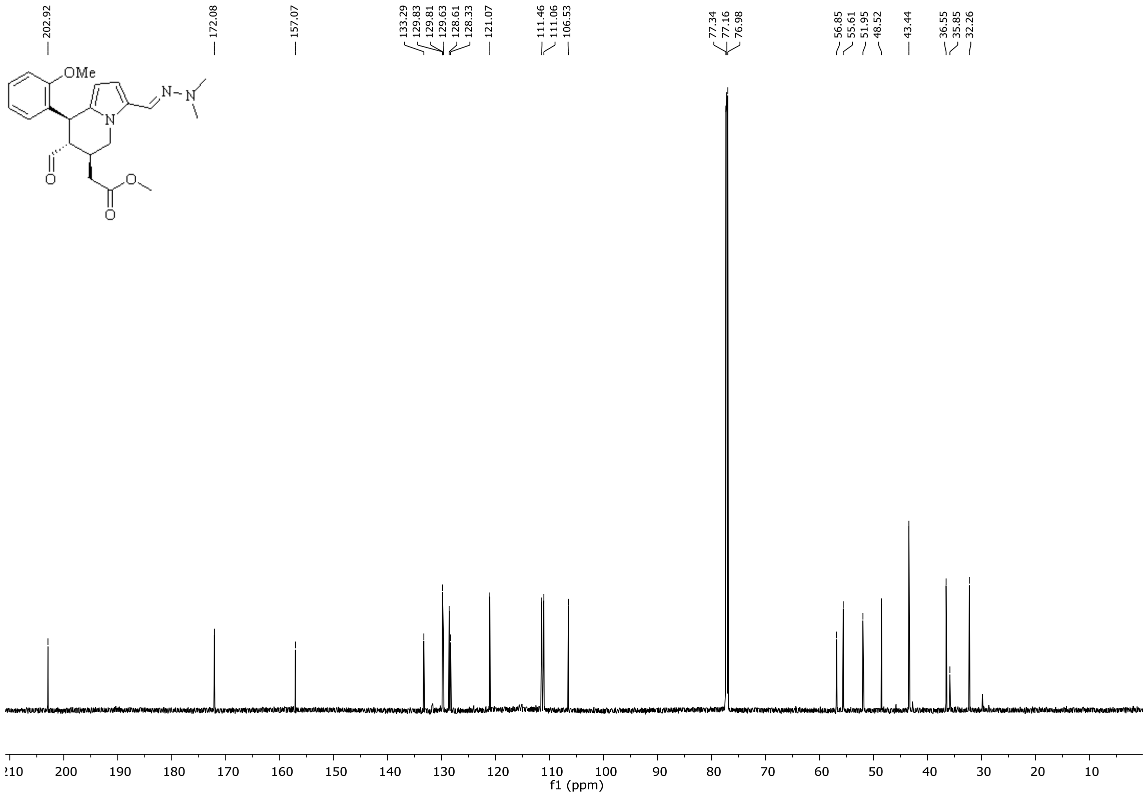

Methyl 2-((6*S*,7*S*,8*S*)-8-(4-cyanophenyl)-3-((*E*)-(2,2-dimethylhydrazono)methyl)-7-formyl-5,6,7,8-tetrahydroindolizin-6-yl)acetate (**4e**)

^{1}H NMR (700 MHz, $CDCl_3$)

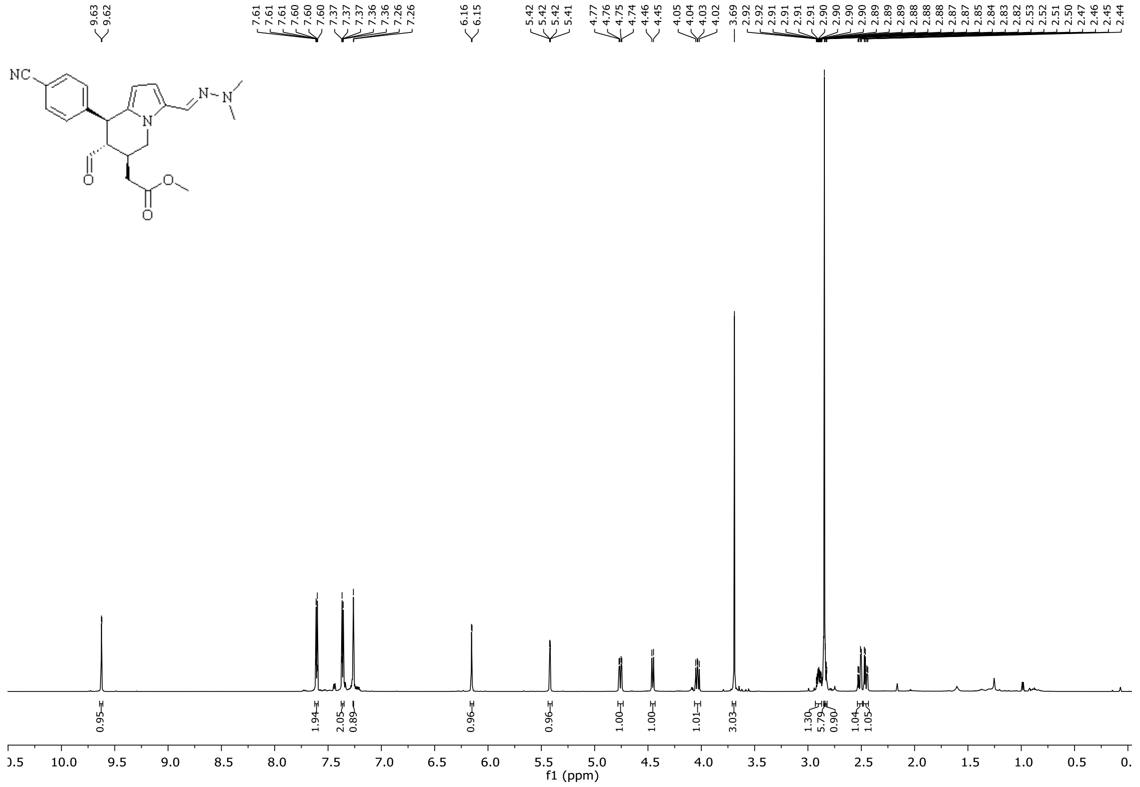

^{13}C NMR (176 MHz, $CDCl_3$)

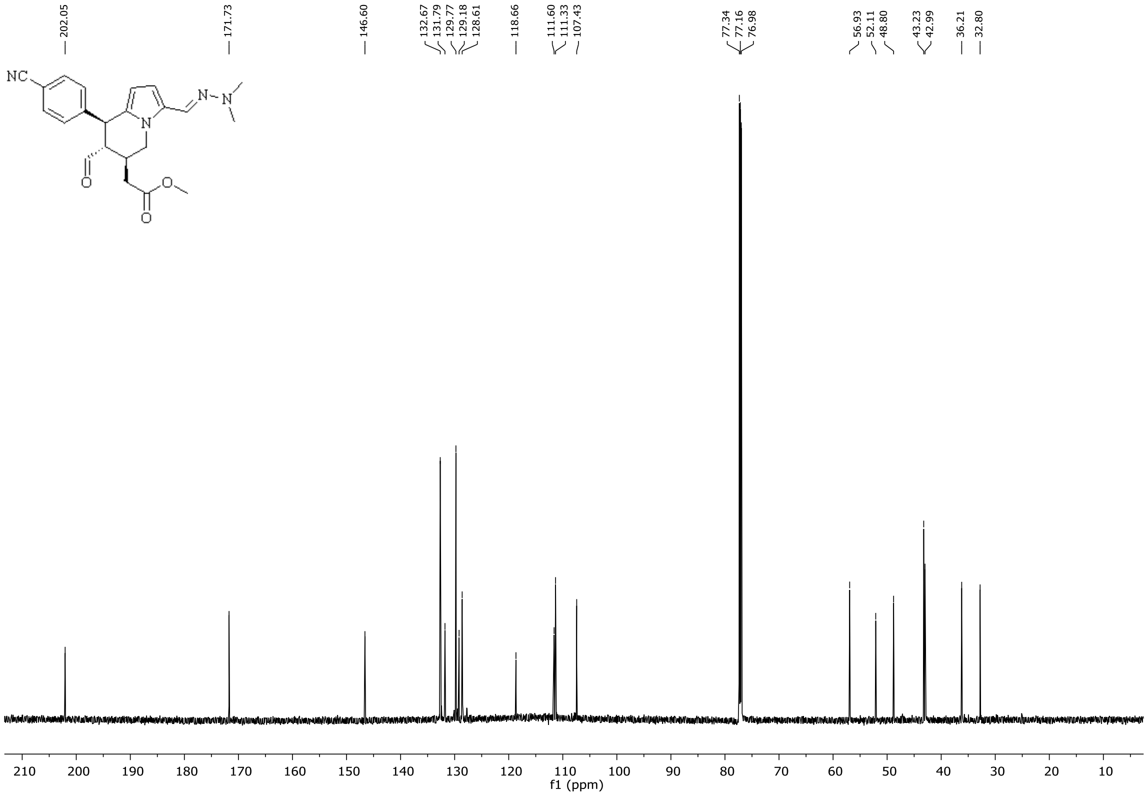

Methyl 2-((6*S*,7*S*,8*S*)-3-((*E*)-(2,2-dimethylhydrazono)methyl)-7-formyl-8-(4-(trifluoromethyl)phenyl)-5,6,7,8-tetrahydroindolizin-6-yl)acetate (**4f**)

^{1}H NMR (700 MHz, $CDCl_3$)

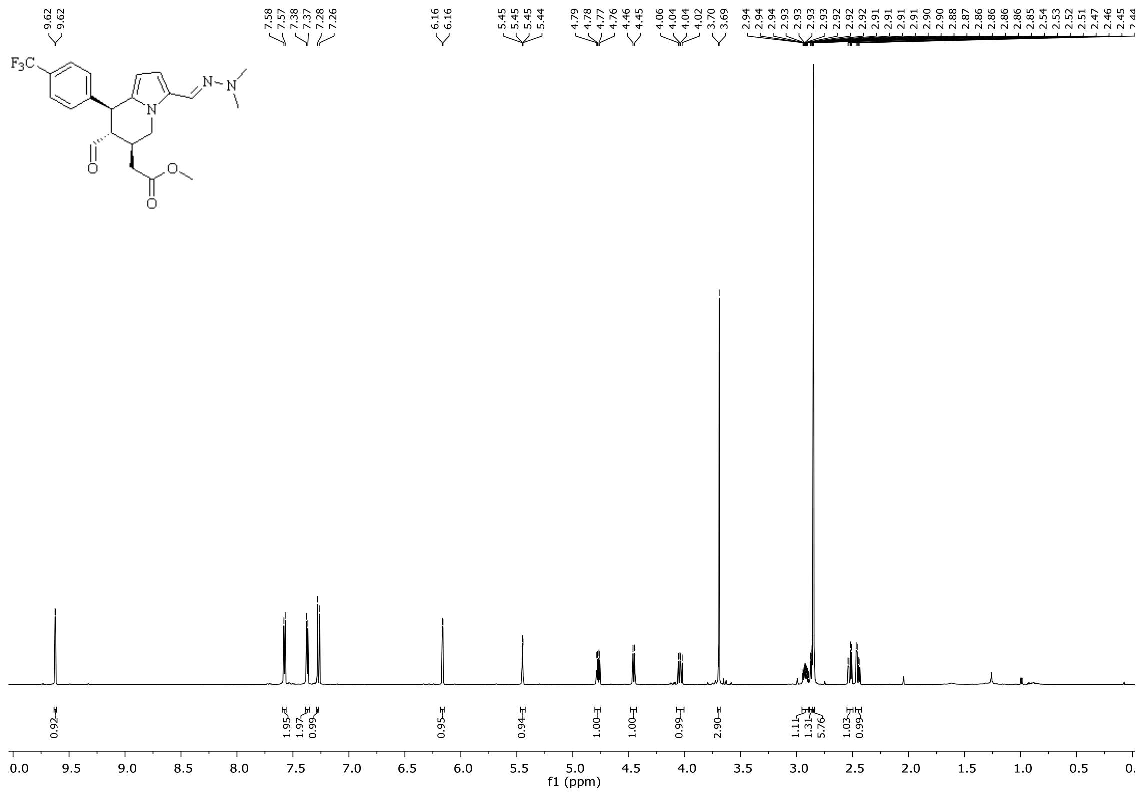

^{13}C NMR (176 MHz, $CDCl_3$)

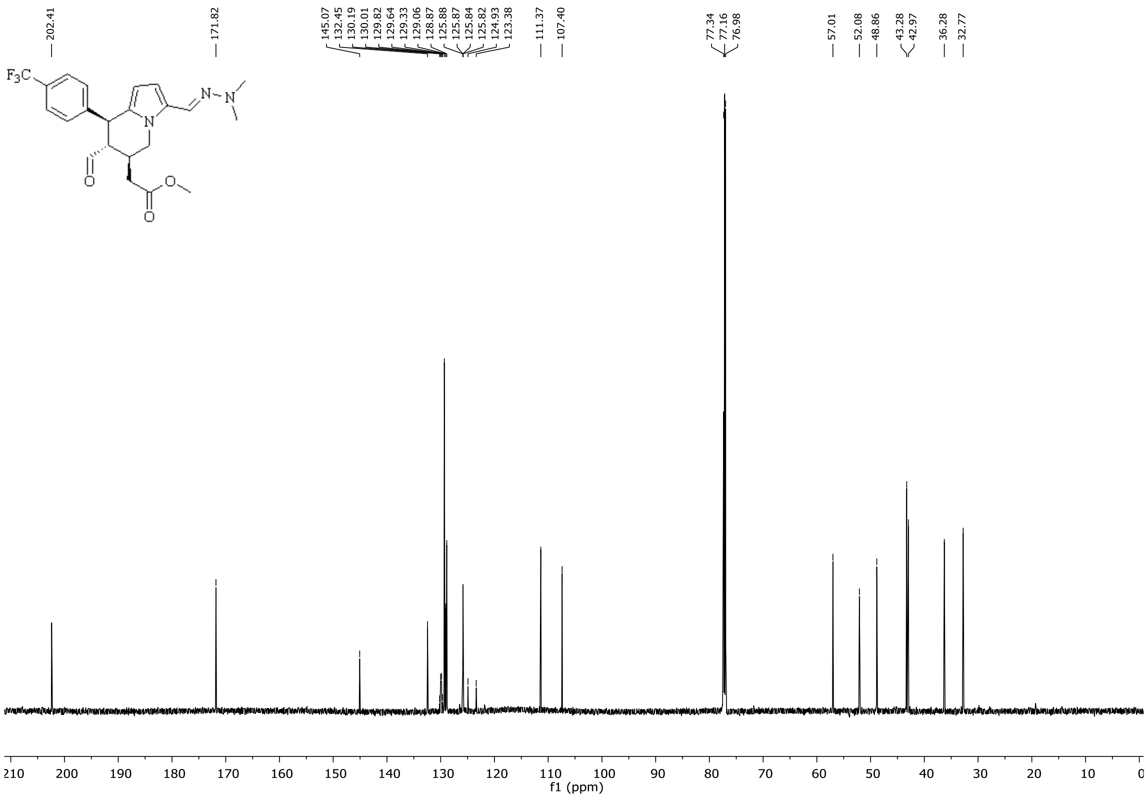

Methyl 2-((6*S*,7*S*,8*S*)-3-((*E*)-(2,2-dimethylhydrazono)methyl)-7-formyl-8-(4-nitrophenyl)-5,6,7,8-tetrahydroindolizin-6-yl)acetate (**4g**)

^{1}H NMR (700 MHz, $CDCl_3$)

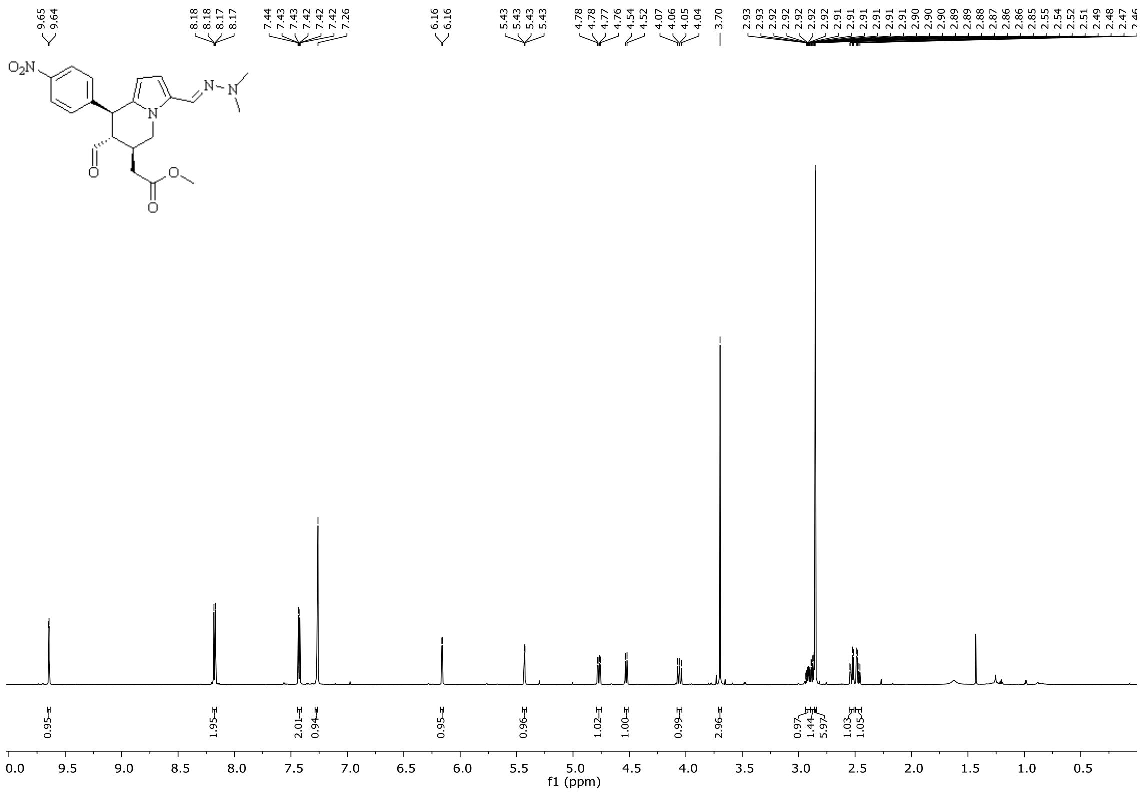

^{13}C NMR (176 MHz, $CDCl_3$)

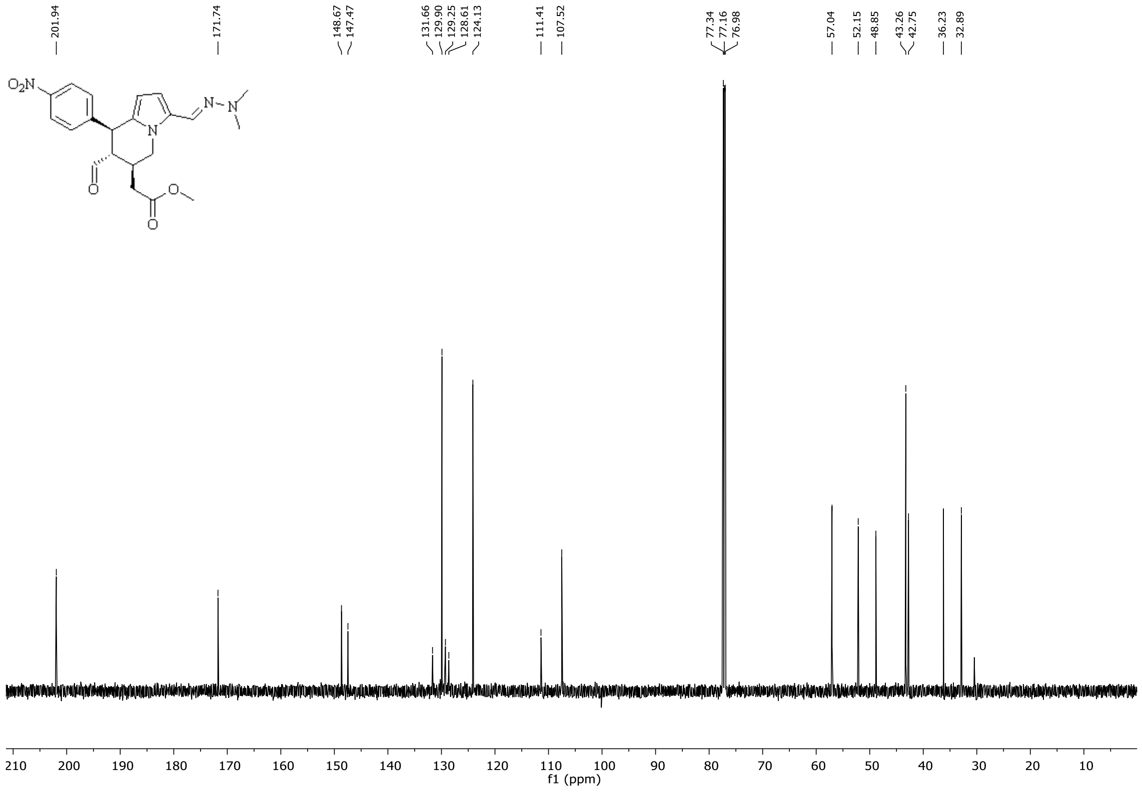

Methyl 2-((6S,7S,8S)-3-((E)-(2,2-dimethylhydrazono)methyl)-7-formyl-8-(p-tolyl)-5,6,7,8-tetrahydroindolizin-6-yl)acetate (**4h**)

^{1}H NMR (700 MHz, $CDCl_3$)

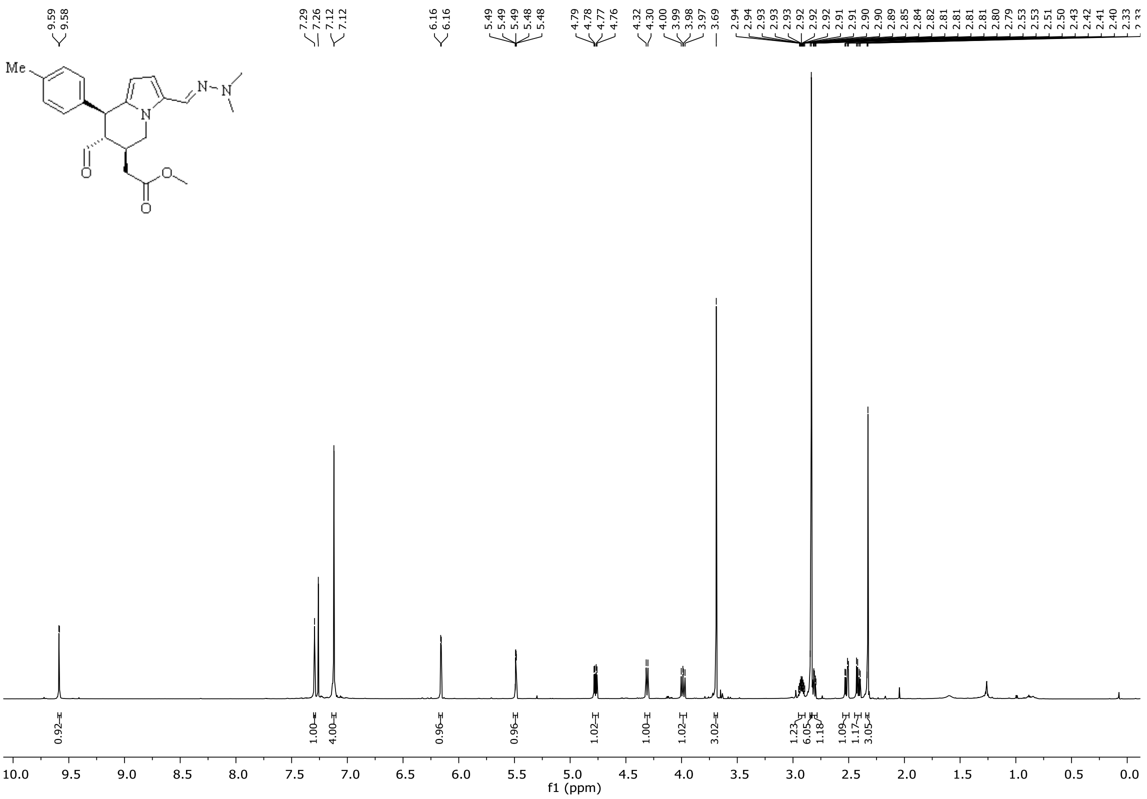

^{13}C NMR (176 MHz, $CDCl_3$)

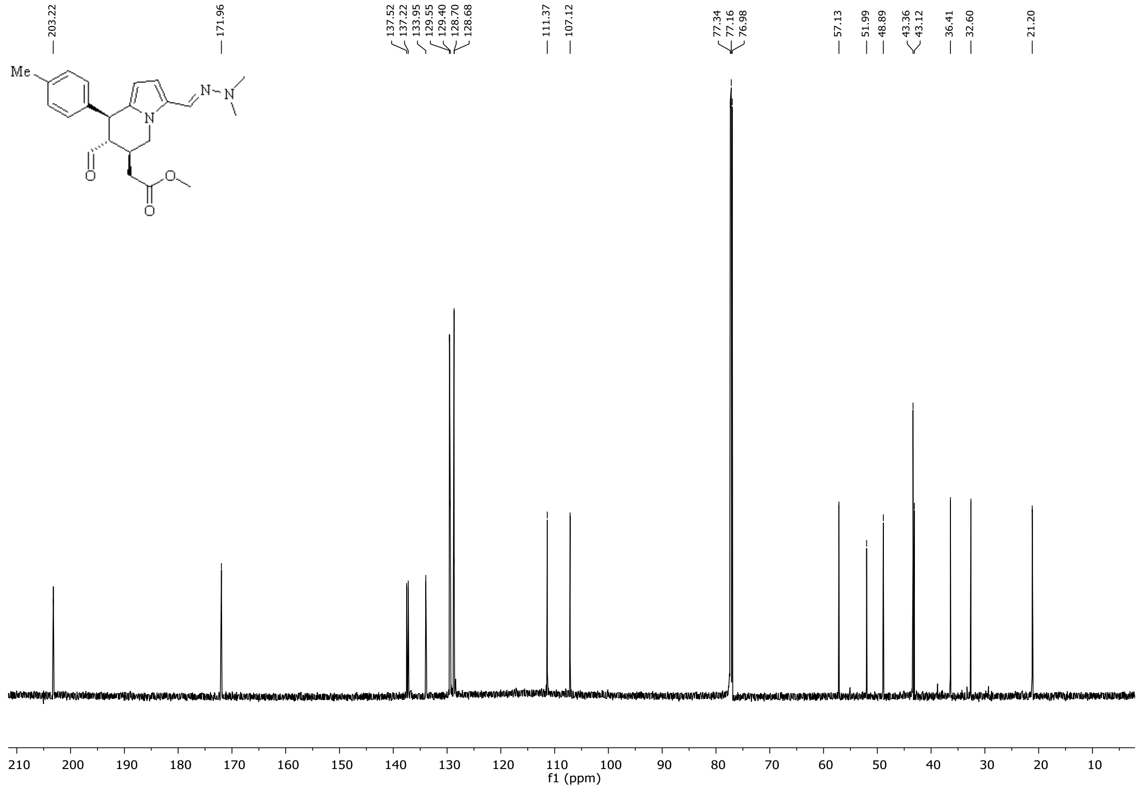

Methyl 2-((6*S*,7*S*,8*S*)-8-(4-chlorophenyl)-3-((*E*)-(2,2-dimethylhydrazono)methyl)-7-formyl-5,6,7,8-tetrahydroindolizin-6-yl)acetate (**4i**)

^{1}H NMR (700 MHz, $CDCl_3$)

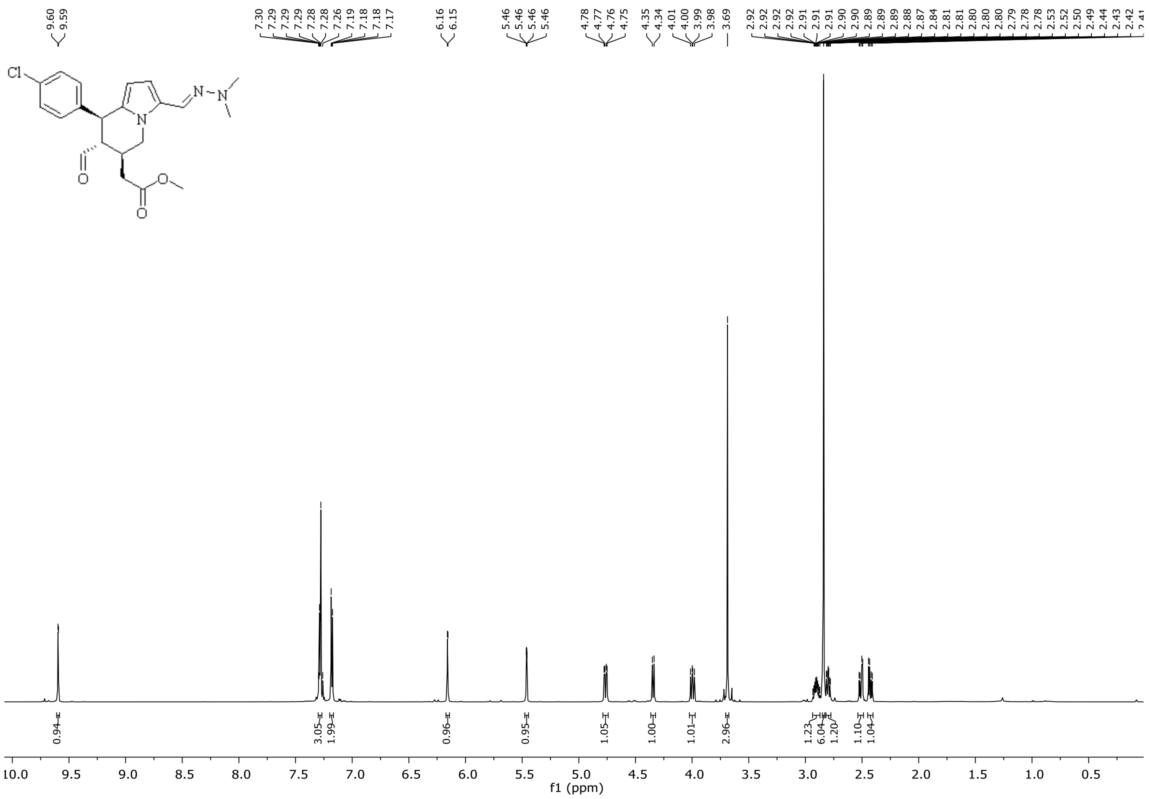

^{13}C NMR (176 MHz, $CDCl_3$)

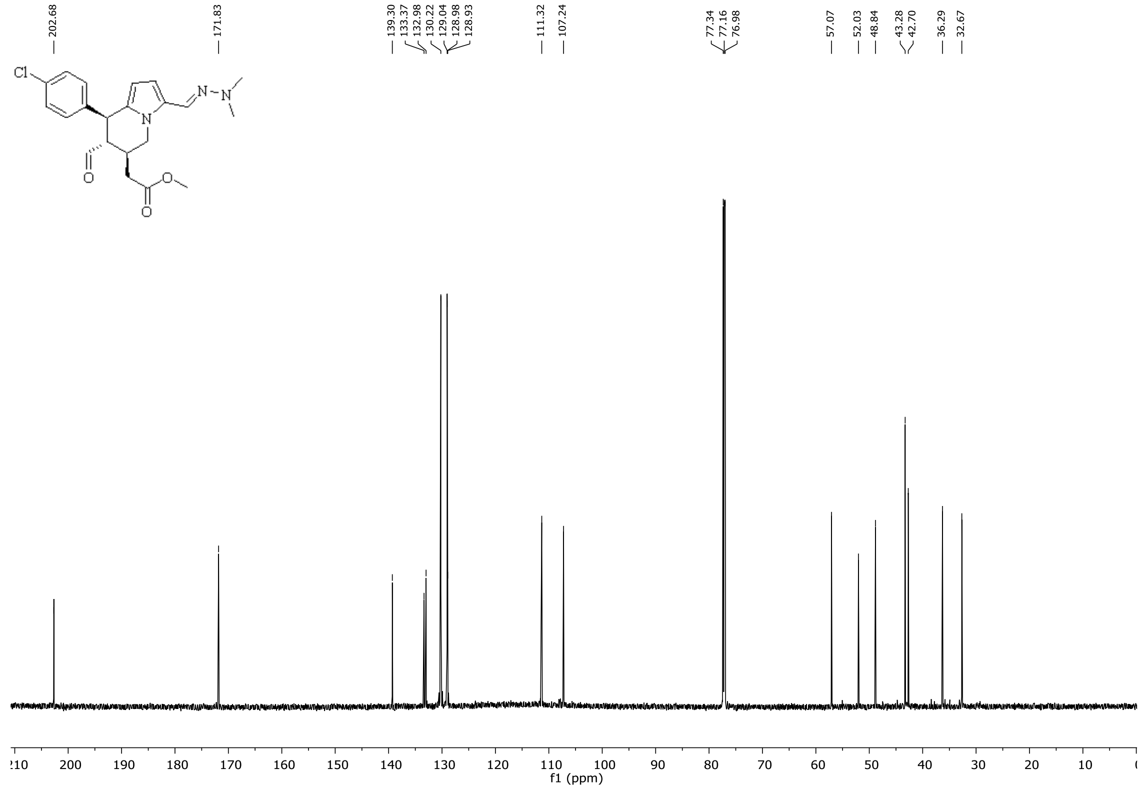

Methyl 2-((6*S*,7*S*,8*S*)-8-(2,4-dichlorophenyl)-3-((*E*)-(2,2-dimethylhydrazono)methyl)-7-formyl-5,6,7,8-tetrahydroindolizin-6-yl)acetate (**4j**)

^{1}H NMR (700 MHz, $CDCl_3$)

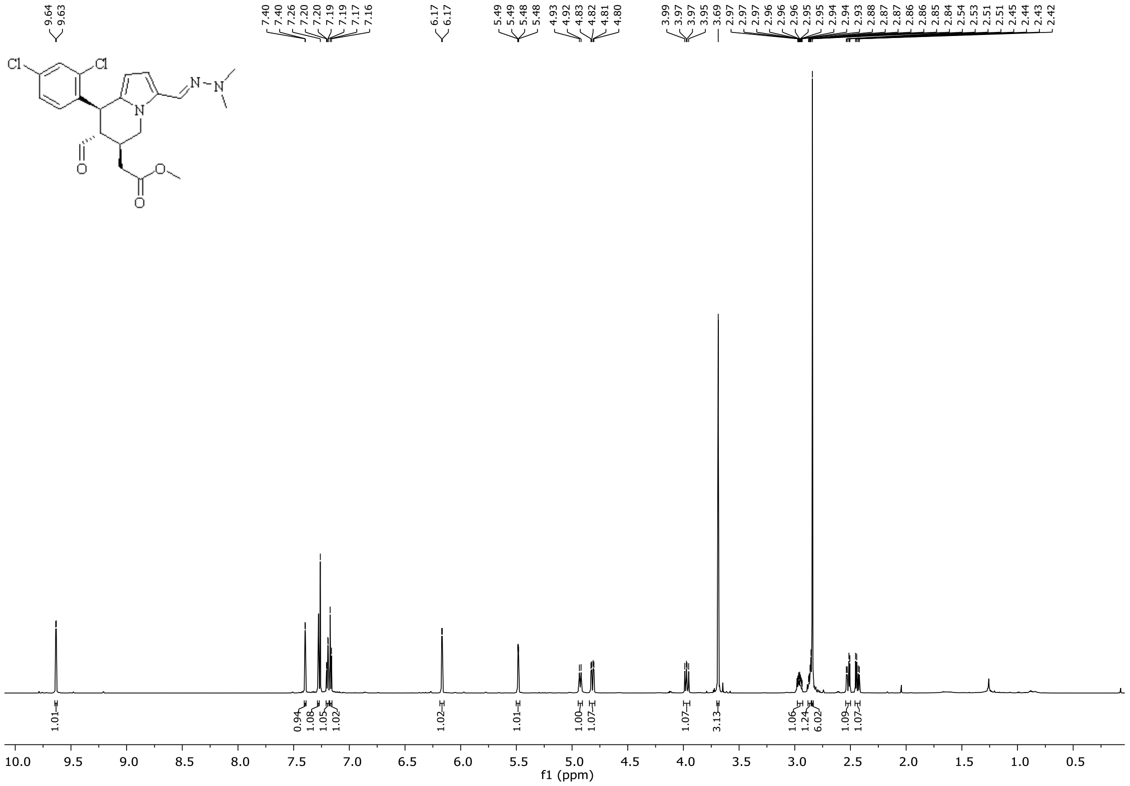

^{13}C NMR (176 MHz, $CDCl_3$)

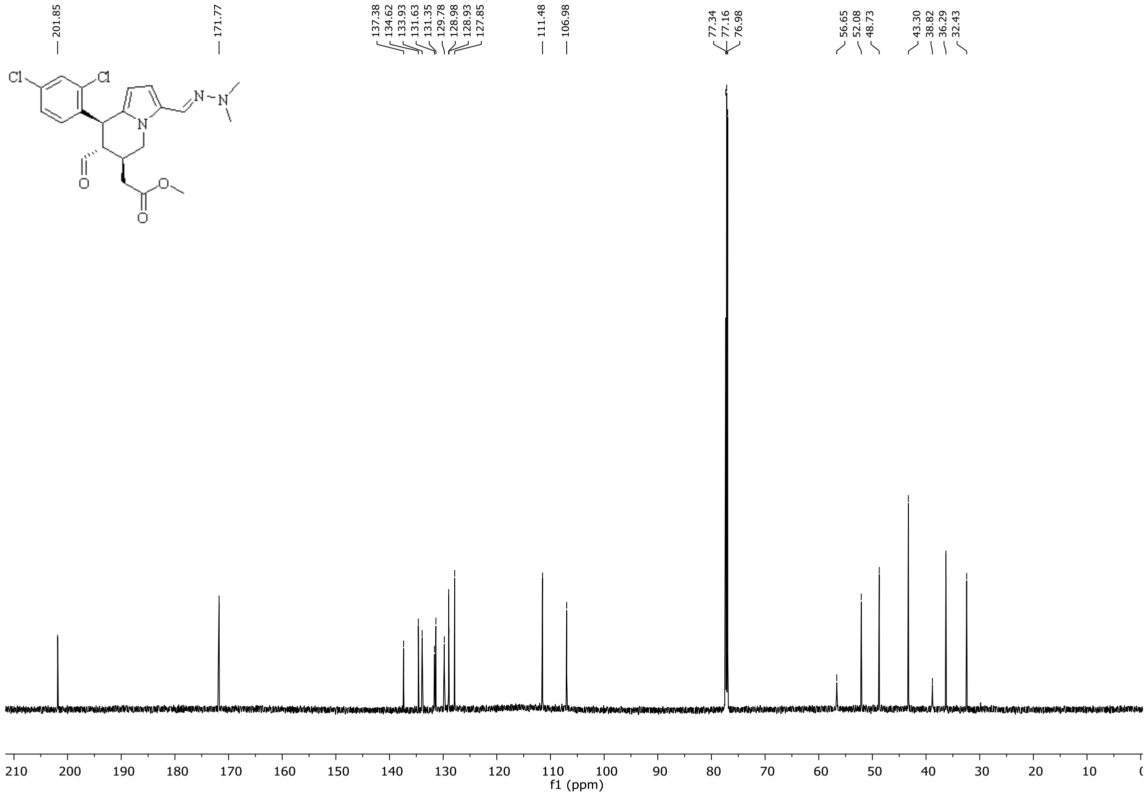

Methyl 2-((6*S*,7*S*,8*S*)-3-((*E*)-(2,2-dimethylhydrazono)methyl)-7-formyl-8-(naphthalen-1-yl)-5,6,7,8-tetrahydroindolizin-6-yl)acetate (**4k**)

^{1}H NMR (700 MHz, $CDCl_3$)

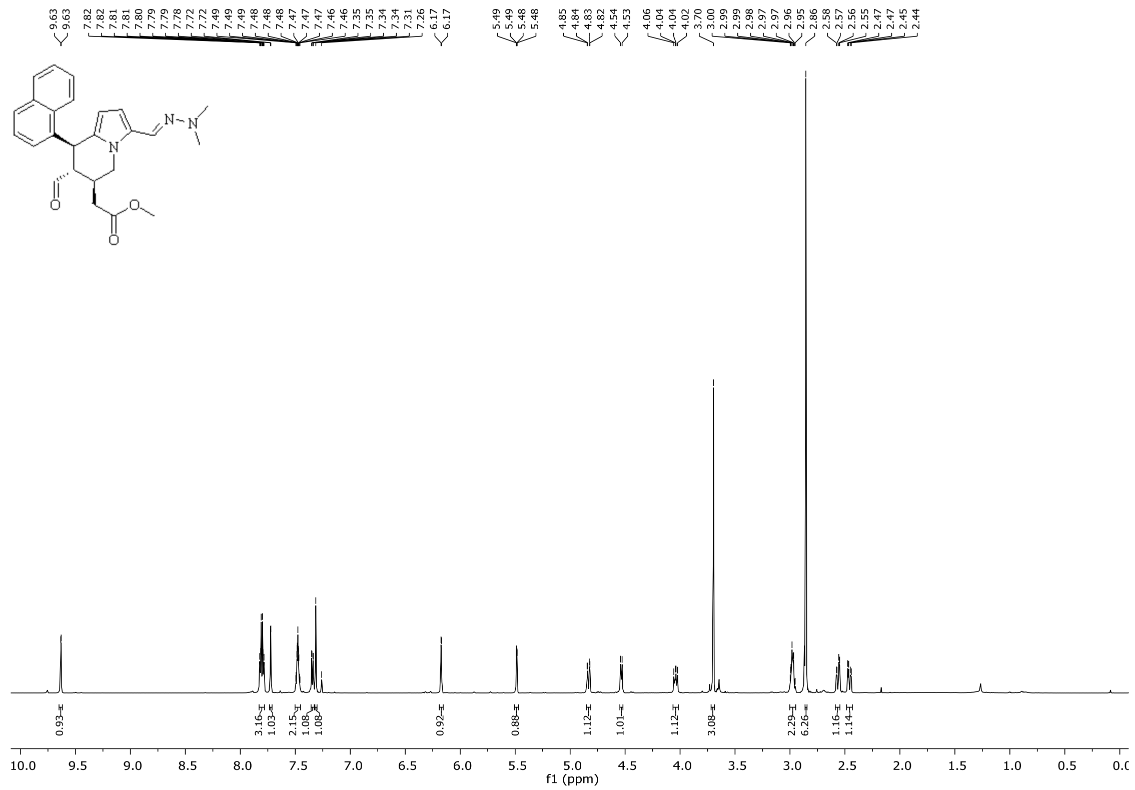

^{13}C NMR (176 MHz, $CDCl_3$)

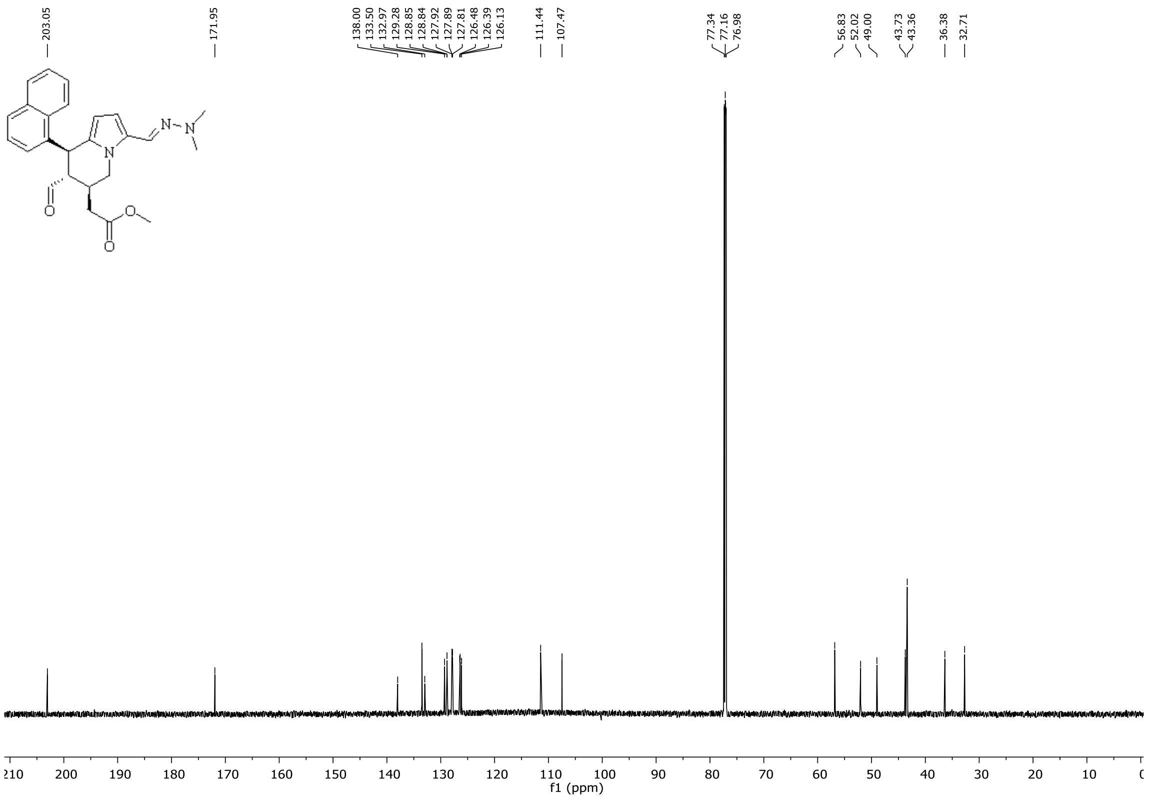

Methyl 2-((6*S*,7*S*,8*R*)-3-((*E*)-(2,2-dimethylhydrazono)methyl)-7-formyl-8-methyl-5,6,7,8-tetrahydroindolizin-6-yl)acetate (**4l**)

^{1}H NMR (700 MHz, CDCl$_3$)

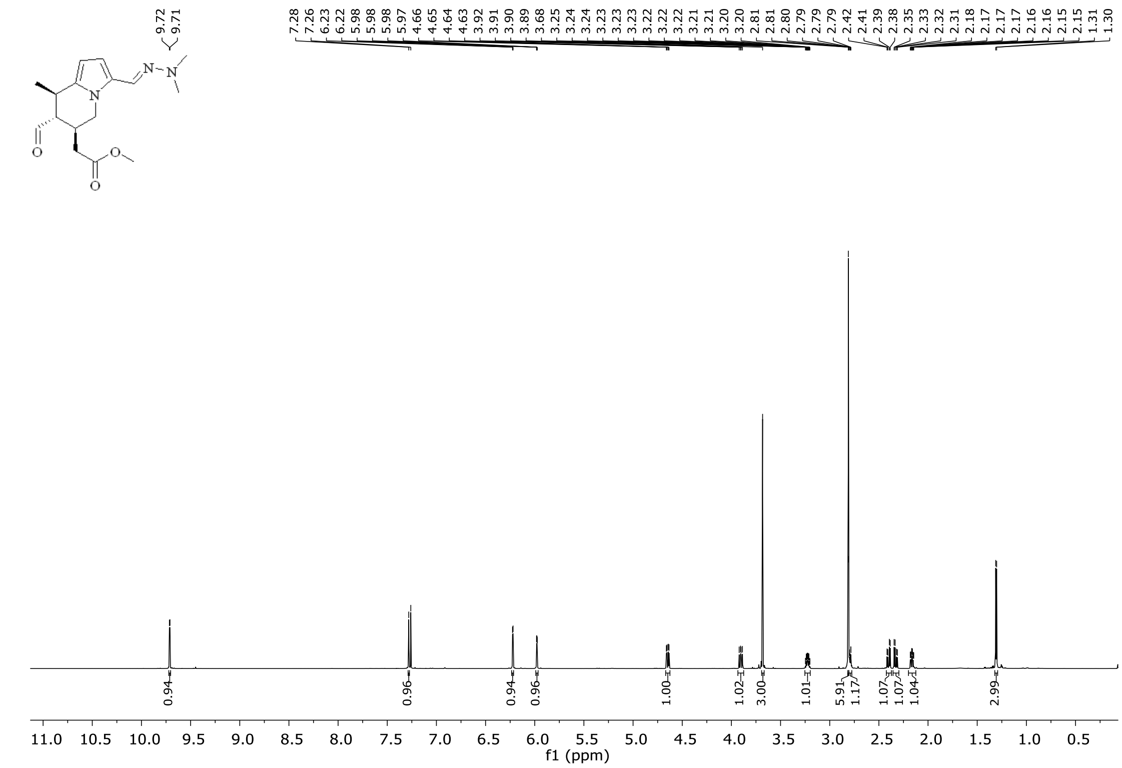

^{13}C NMR (176 MHz, CDCl$_3$)

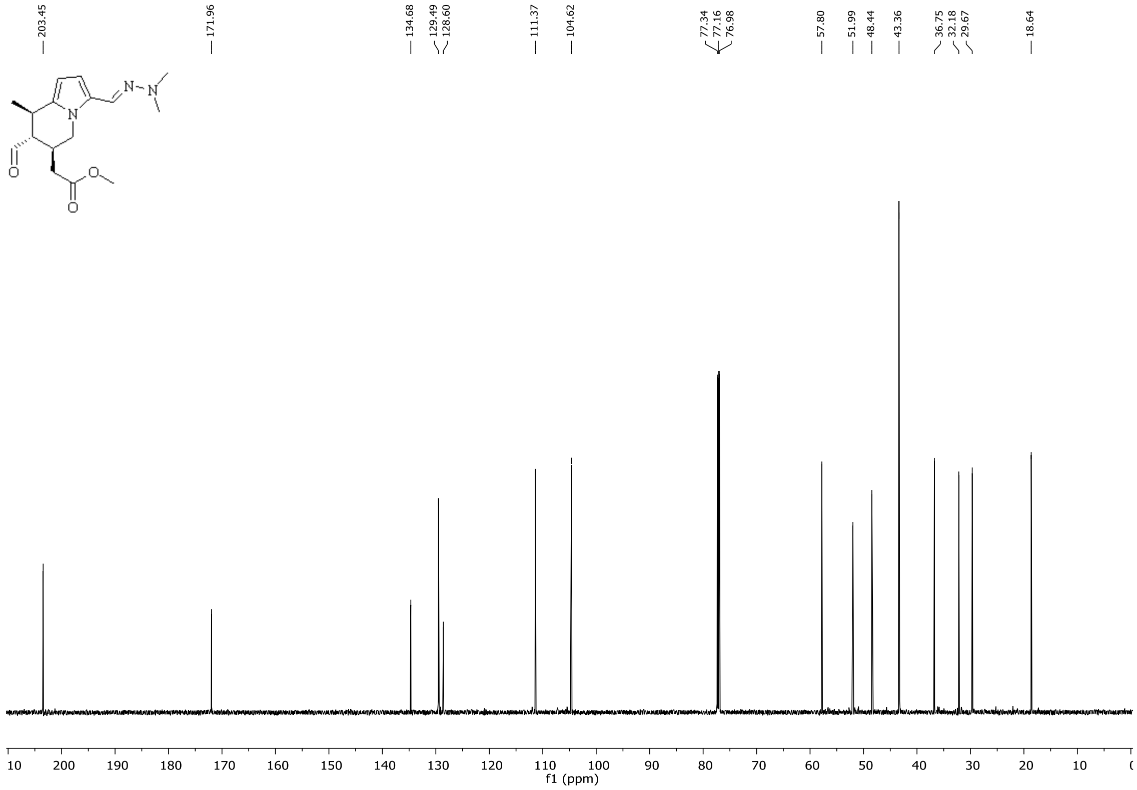

Methyl 2-((6*S*,7*S*,8*R*)-3-((*E*)-(2,2-dimethylhydrazono)methyl) -7-formyl-8-ethyl -5,6,7,8-tetrahydroindolizin-6-yl)acetate (**4m**)

^{1}H NMR (700 MHz, $CDCl_3$)

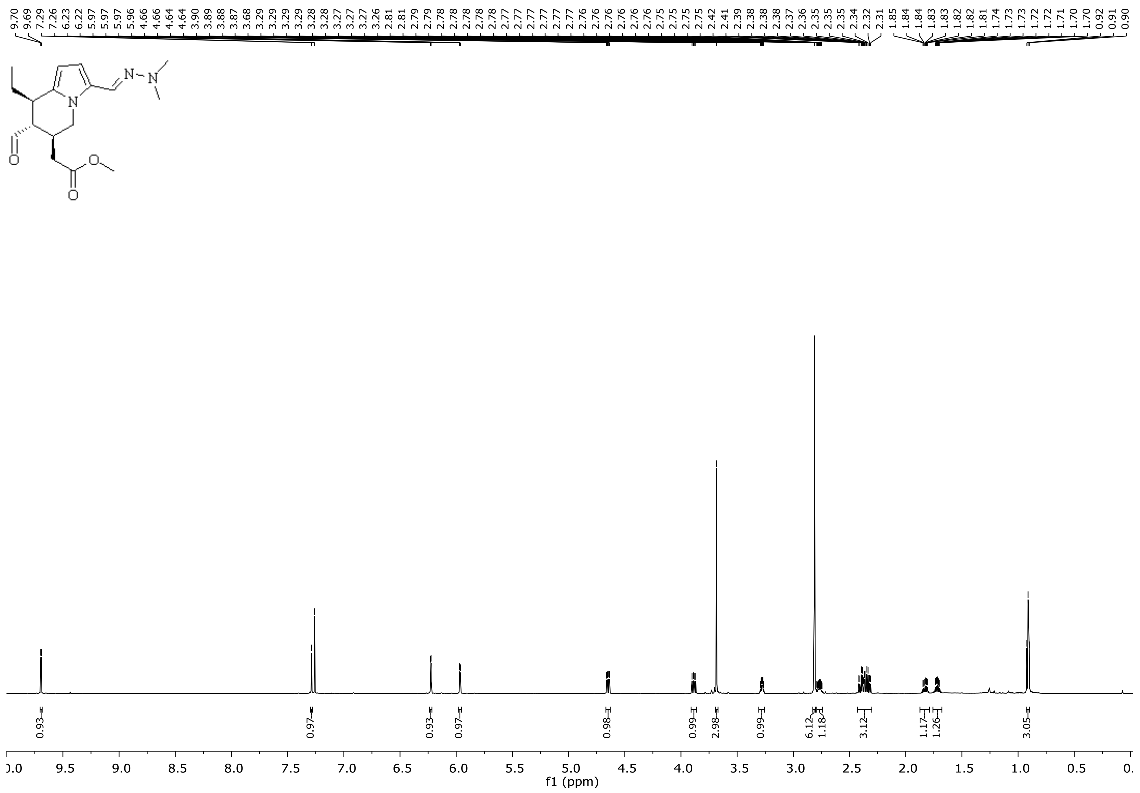

^{13}C NMR (176 MHz, $CDCl_3$)

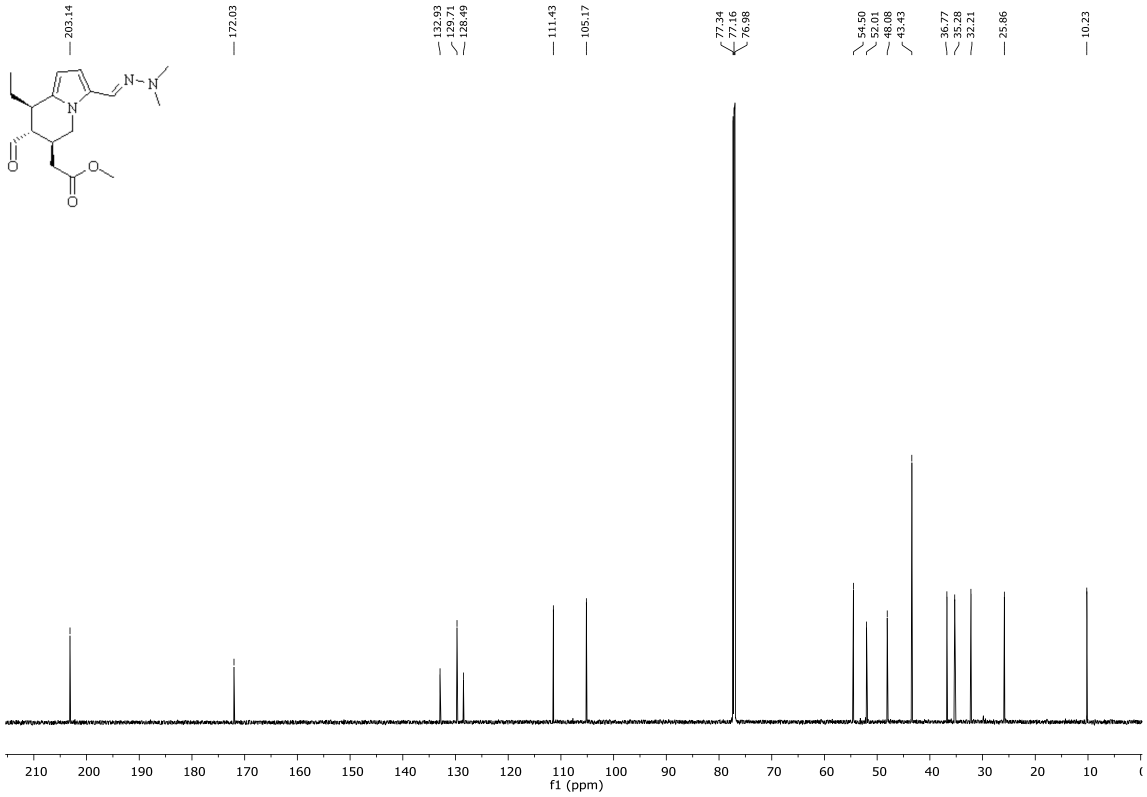

Methyl 2-((6*S*,7*S*,8*R*)-3-((*E*)-(2,2-dimethylhydrazono)methyl)-7-formyl-8-((*Z*)-hex-3-en-1-yl)-5,6,7,8-tetrahydroindolizin-6-yl)acetate (**4n**)

^{1}H NMR (700 MHz, $CDCl_3$)

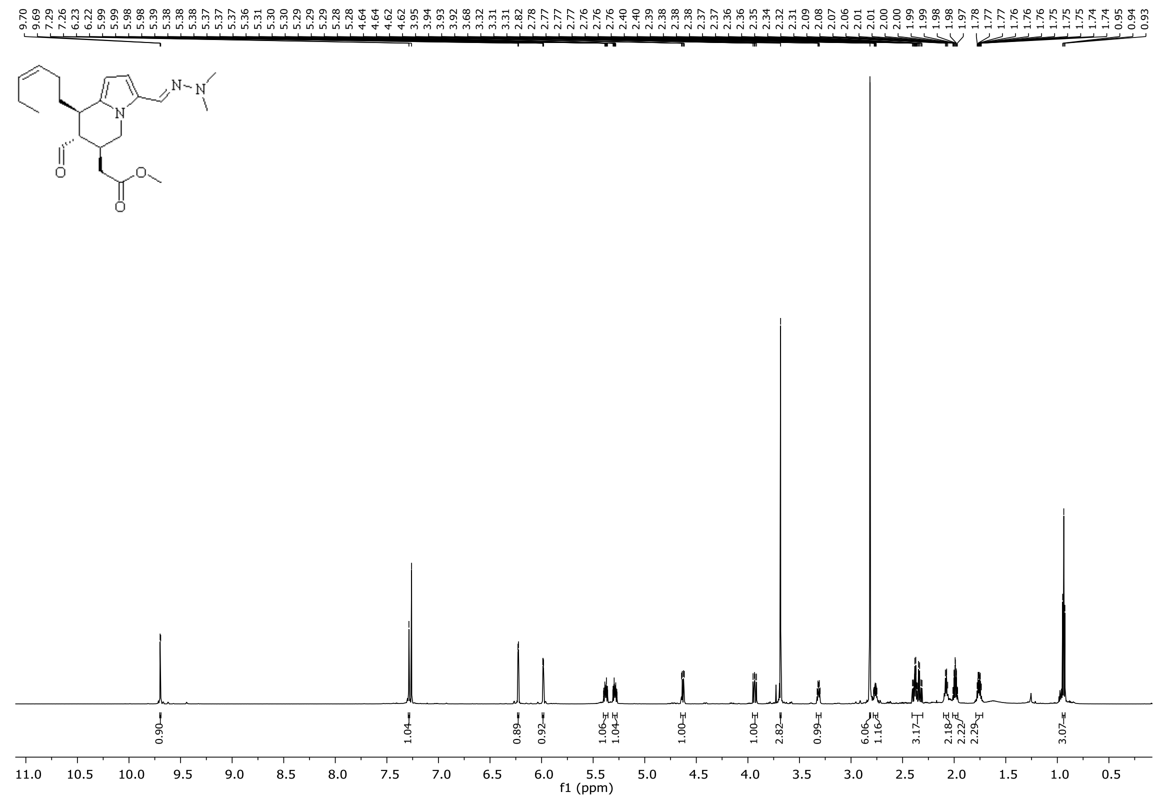

^{13}C NMR (176 MHz, $CDCl_3$)

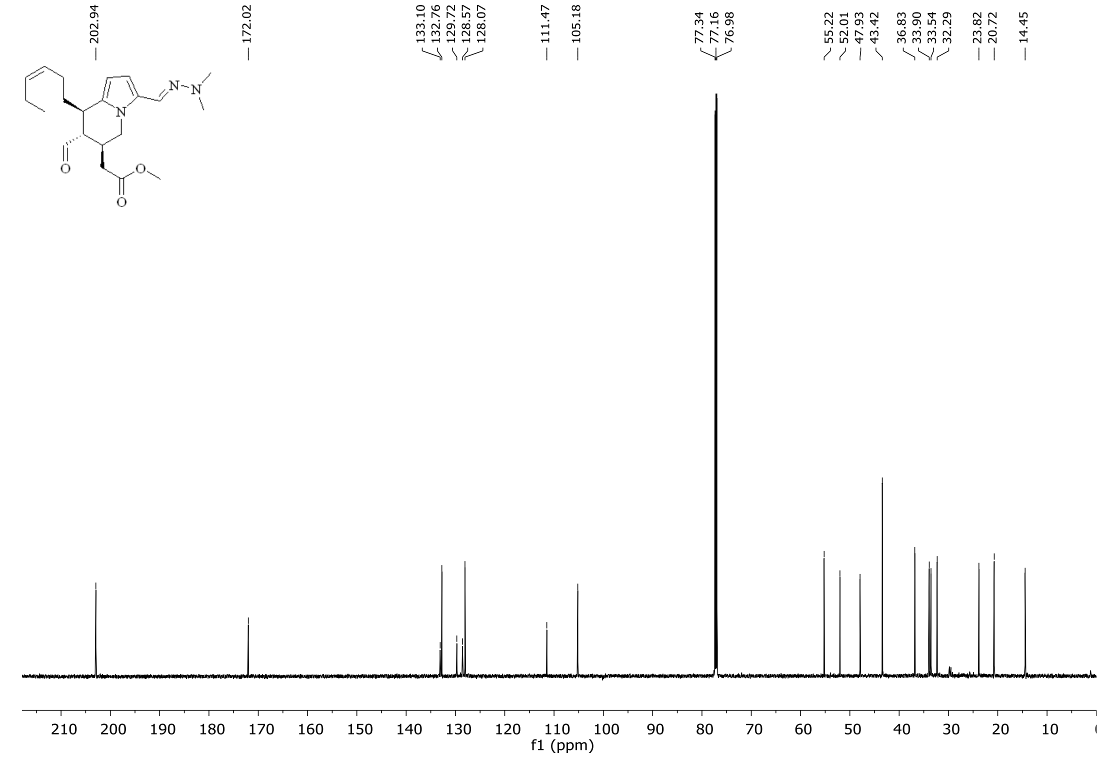

Methyl 2-((6*S*,7*S*,8*R*)-3-((*E*)-(2,2-dimethylhydrazono)methyl)-7-formyl-8-(phenethoxymethyl)-5,6,7,8-tetrahydroindolizin-6-yl)acetate (**4o**)

^{1}H NMR (400 MHz, $CDCl_3$)

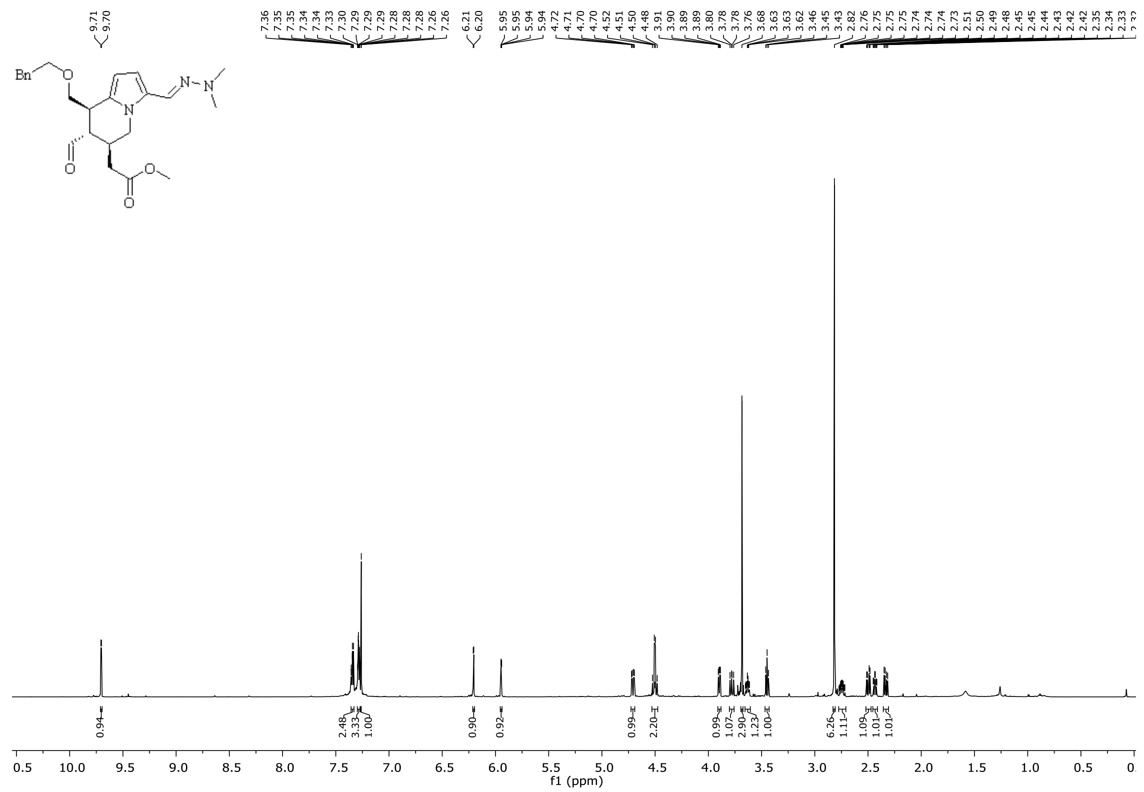

^{13}C NMR (101 MHz, $CDCl_3$)

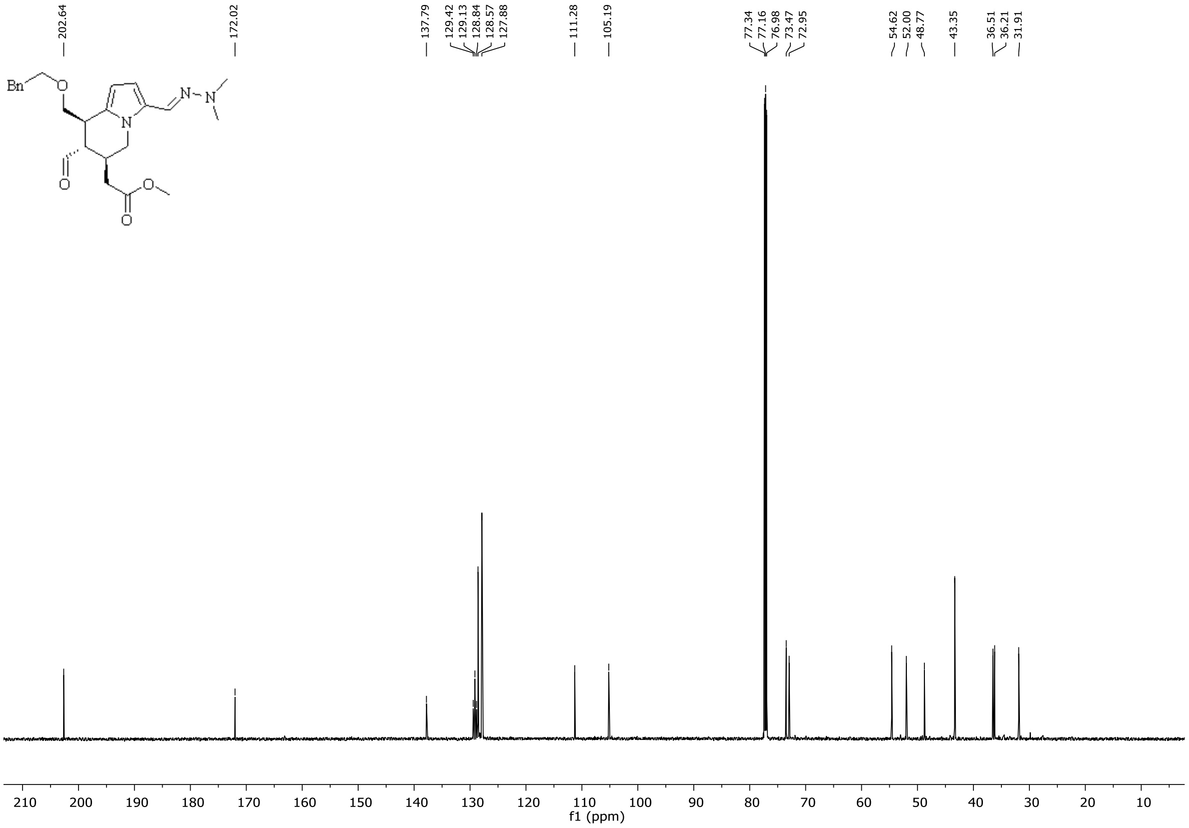

Methyl 2-((6S,7S,8S)-7-formyl-3-((E)-(morpholinoimino)methyl)-8-phenyl-5,6,7,8-tetrahydroindolizin-6-yl)acetate (**4p**)

^{1}H NMR (700 MHz, $CDCl_3$)

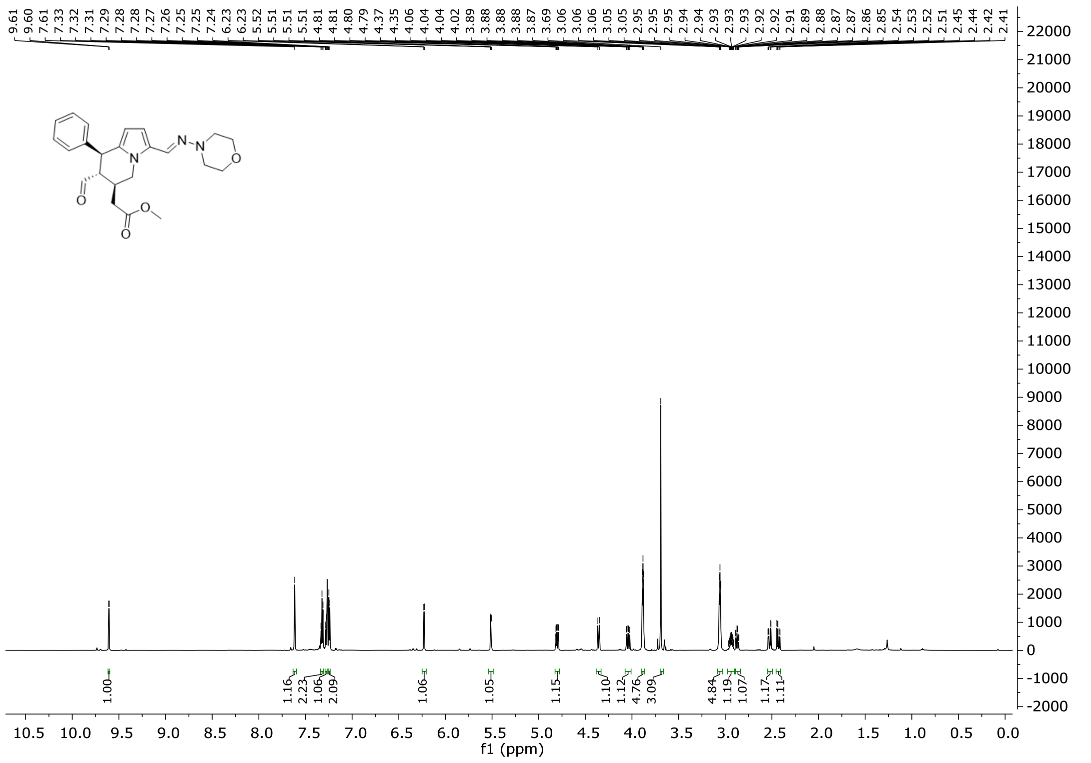

^{13}C NMR (176 MHz, $CDCl_3$)

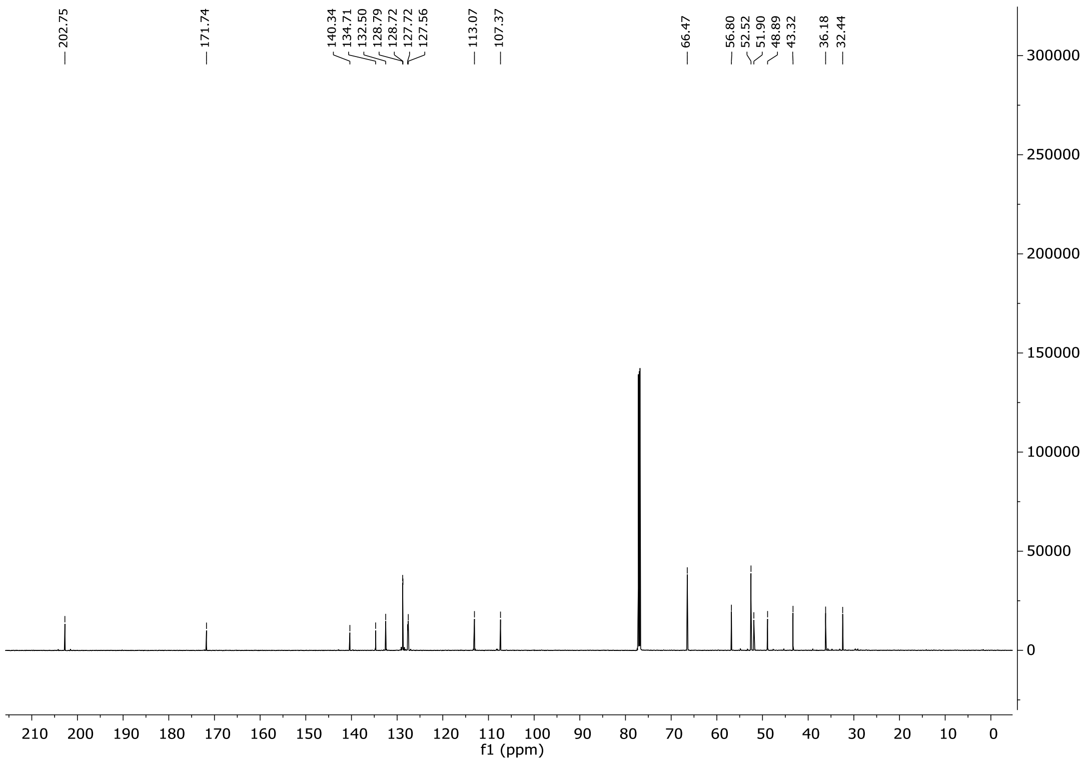

Methyl 2-((6*S*,7*S*,8*S*)-3-cyano-7-formyl-8-phenyl-5,6,7,8-tetrahydroindolizin-6-yl)acetate (**6a**)

^{1}H NMR (400 MHz, $CDCl_3$)

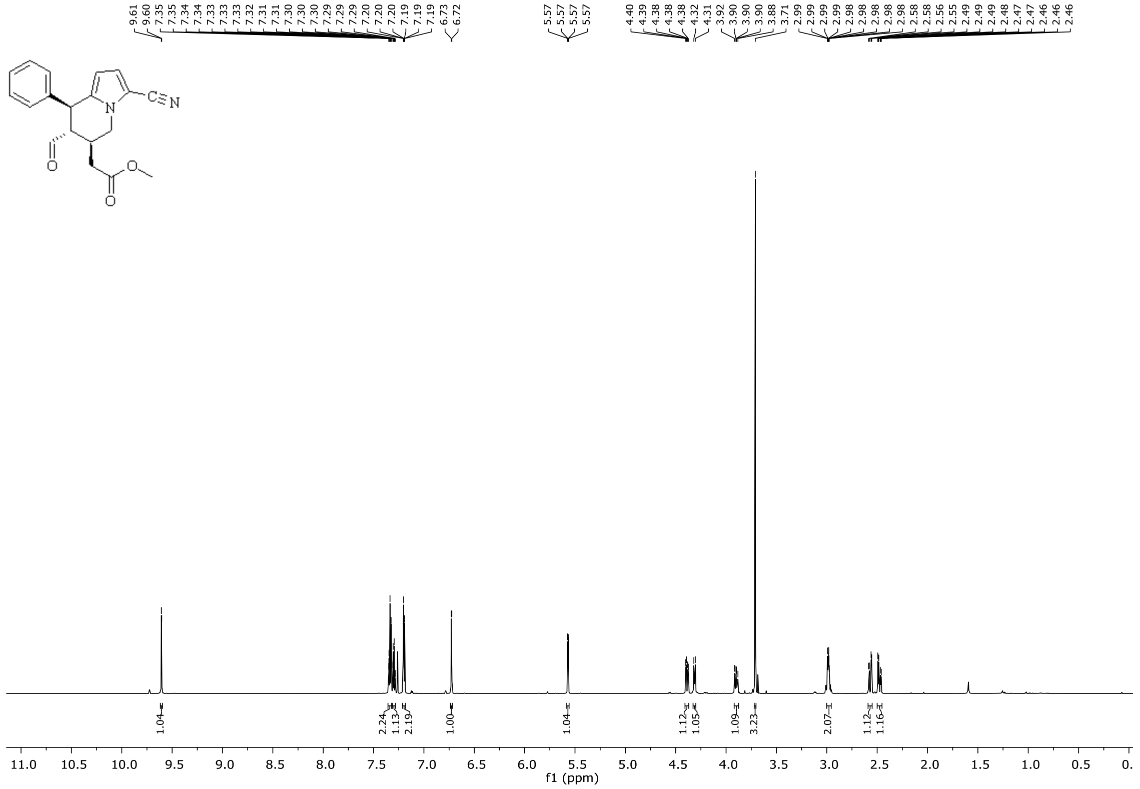

^{13}C NMR (101 MHz, $CDCl_3$)

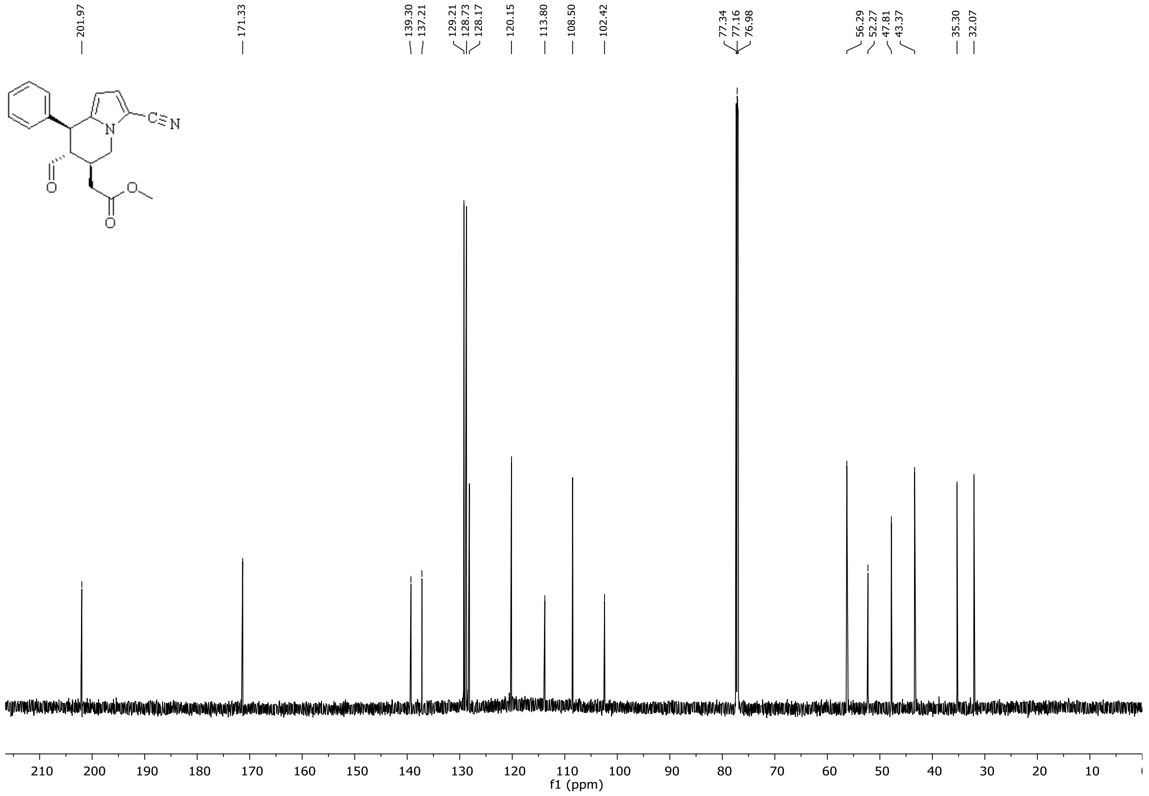

Methyl 2-((6*S*,7*S*,8*S*)-3-cyano-7-((phenylamino)methyl)-8-phenyl-5,6,7,8-tetrahydroindolizin-6-yl)acetate (**7a**)

^{1}H NMR (700 MHz, $CDCl_3$)

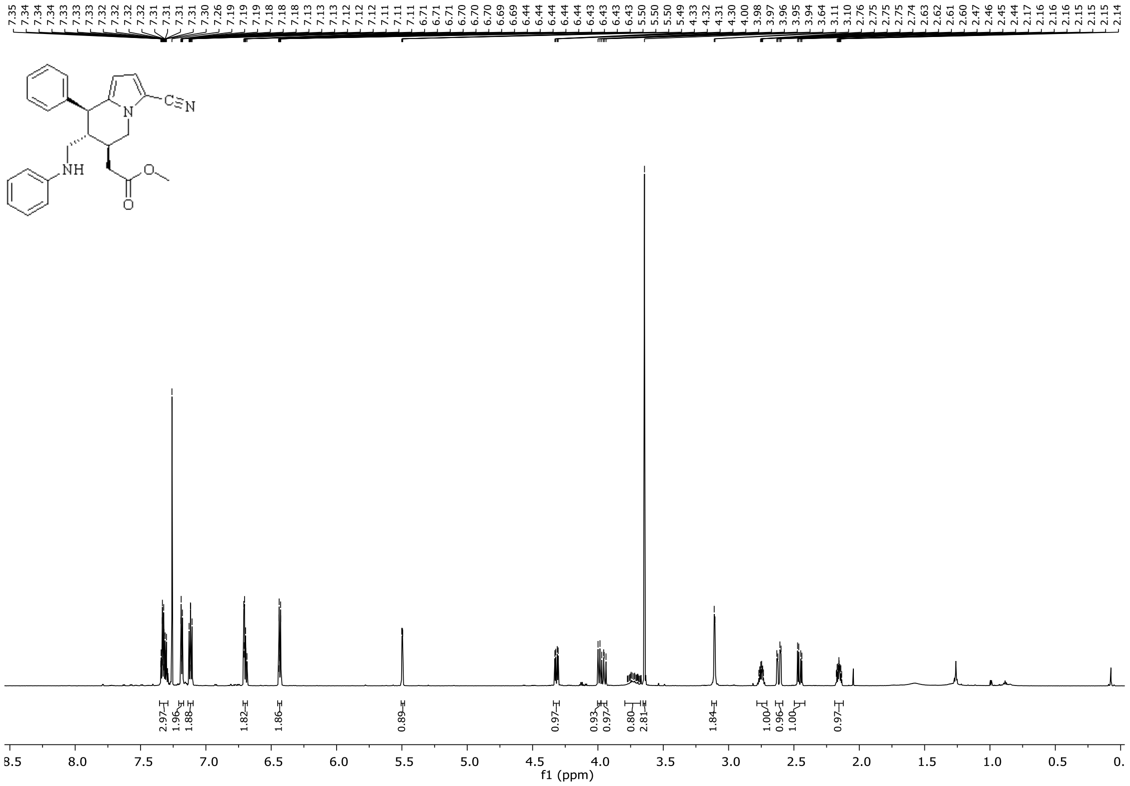

^{13}C NMR (176 MHz, $CDCl_3$)

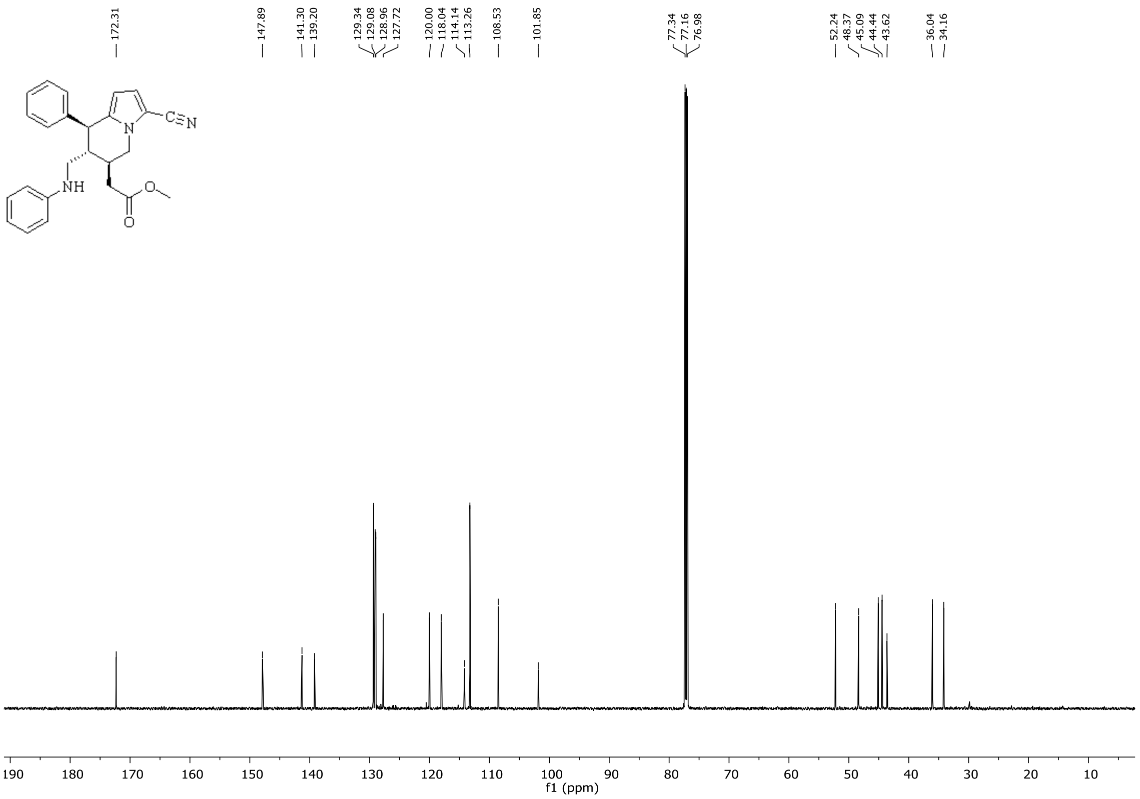

(4a*S*,10*S*,10a*S*)-3-Oxo-2,10-diphenyl-1,2,3,4,4a,5,10,10a-octahydropyrrolo[1,2-*b*][2,6]-naphthyridine-7-carbonitrile (**8a**)

^{1}H NMR (700 MHz, $CDCl_3$)

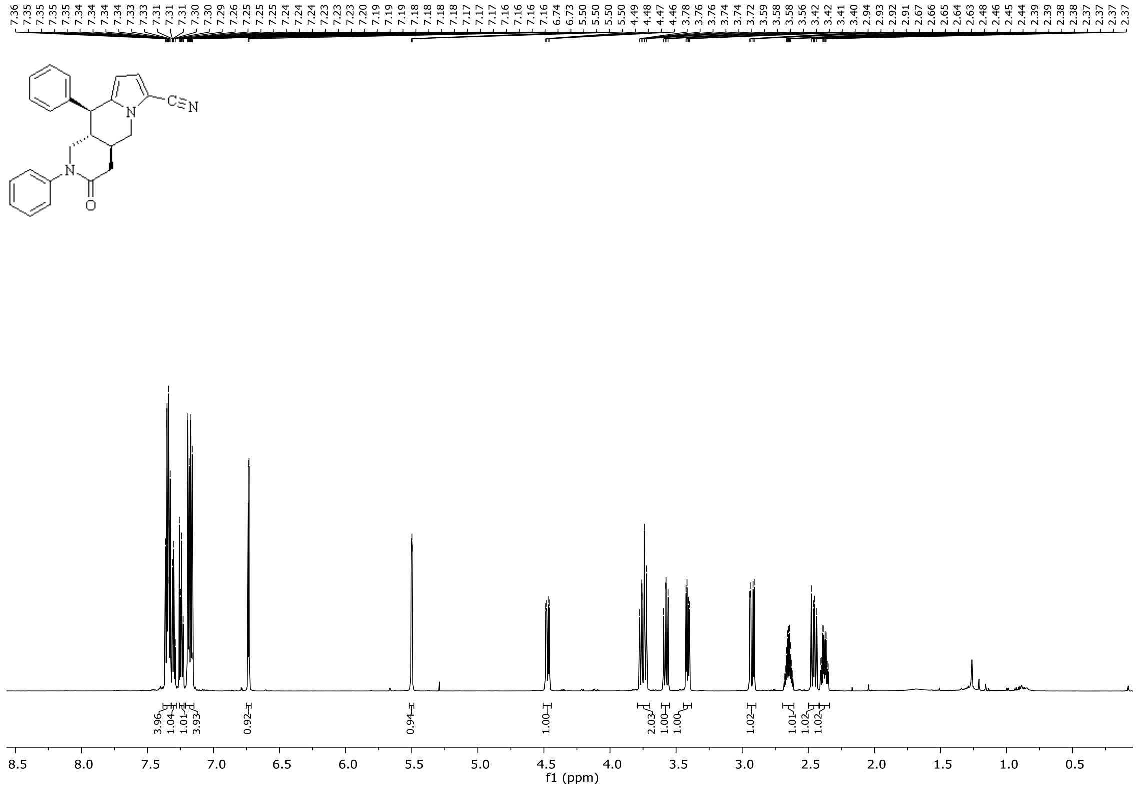

^{13}C NMR (176 MHz, $CDCl_3$)

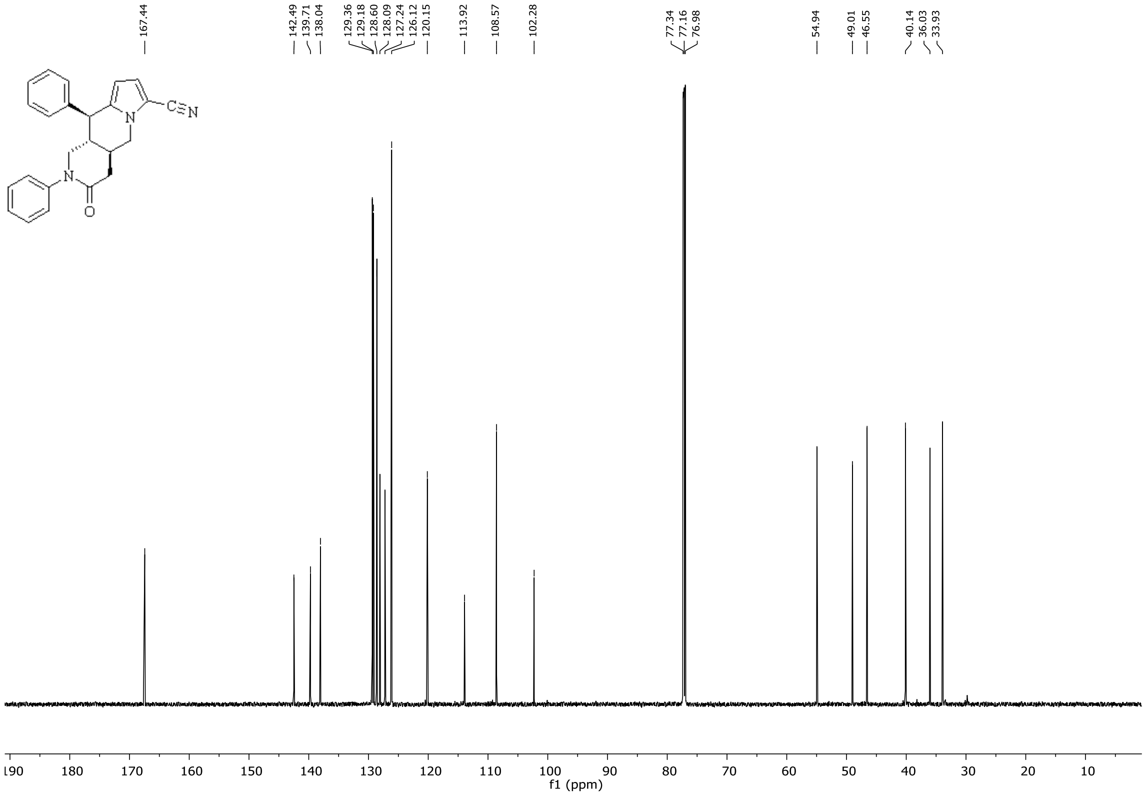

(4a*S*,10*S*,10a*S*)-3-Oxo-10-phenyl-3,4,4a,5,10,10a-hexahydro-1*H*-pyrano[4,3-*f*]indolizine-7-carbonitrile (**9a**)

^{1}H NMR (700 MHz, $CDCl_3$)

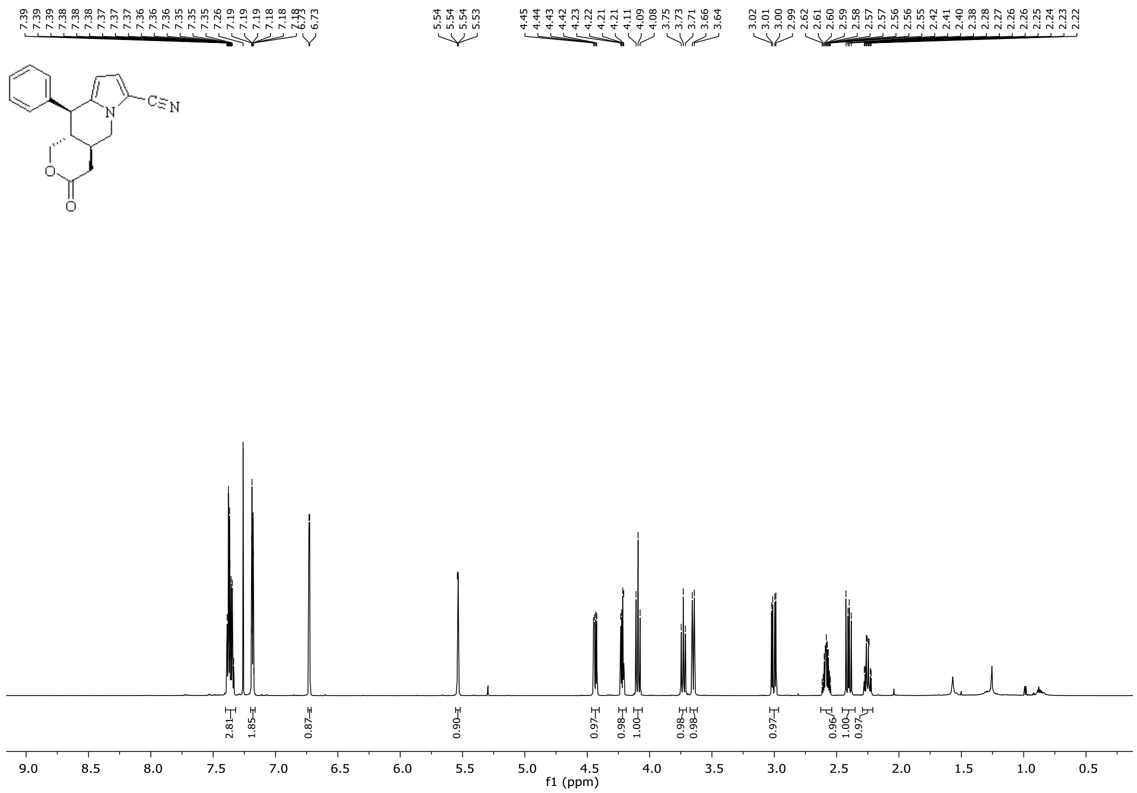

^{13}C NMR (176 MHz, $CDCl_3$)

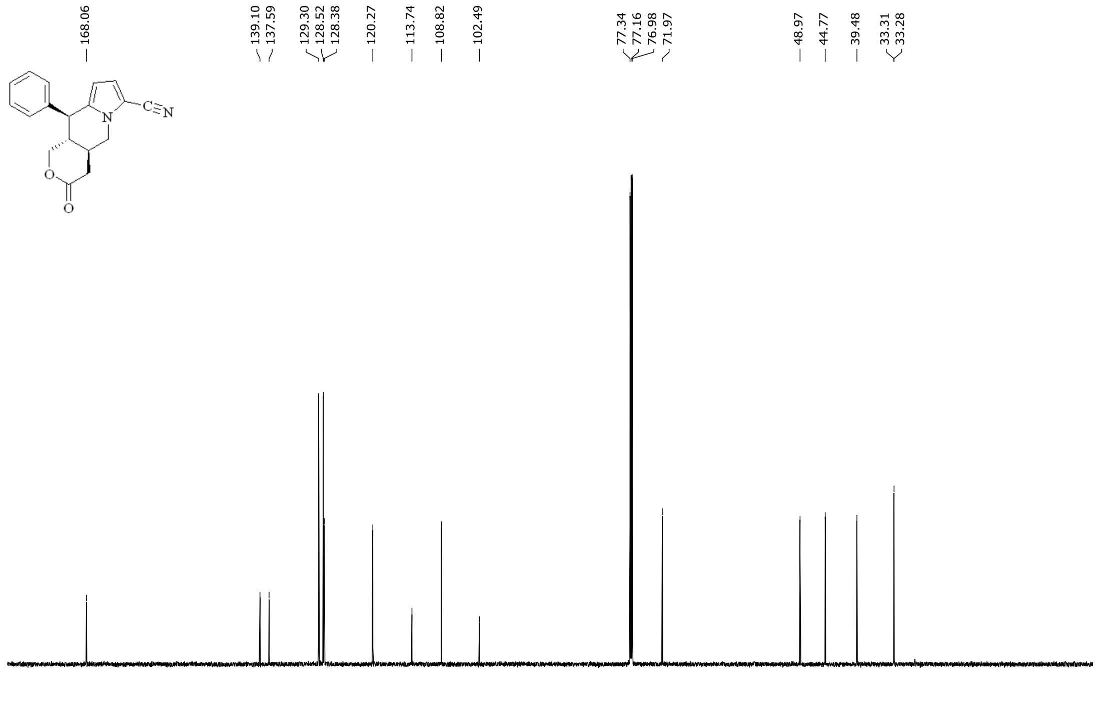

11. UPC^2 and HPLC traces

Methyl 2-((6*S*,7*S*,8*S*)-3-((*E*)-(2,2-dimethylhydrazono)methyl)-7-formyl-8-phenyl-5,6,7,8-tetrahydroindolizin-6-yl)acetate (**4a**)

Racemic sample

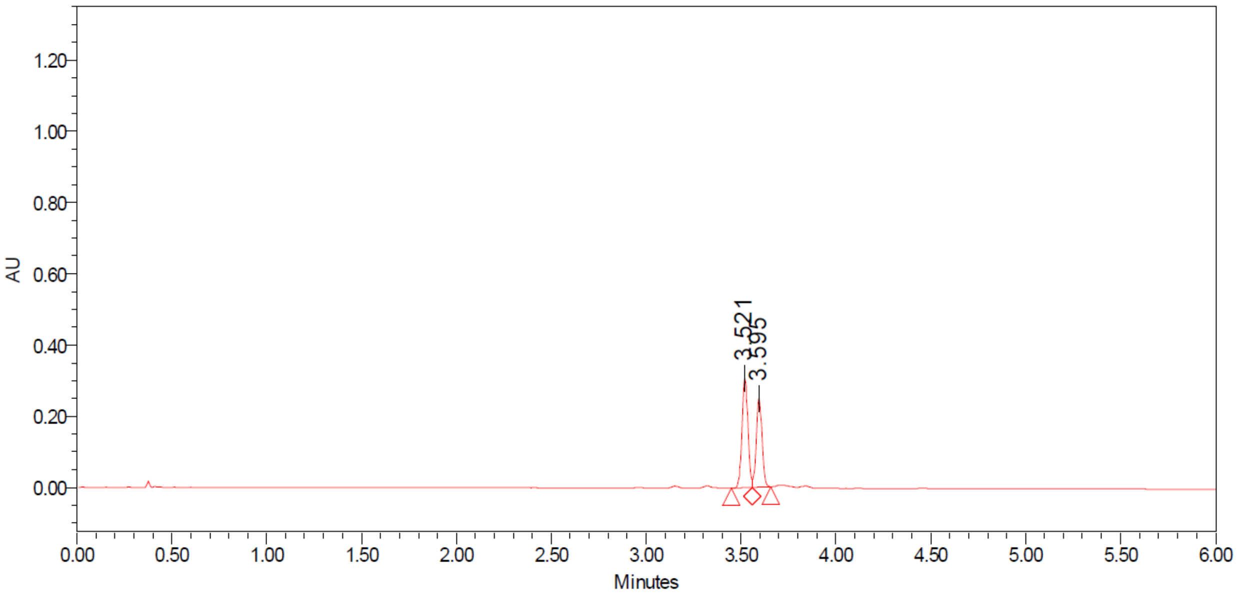

Peak Results

	RT	% Area
1	3.521	55.97
2	3.595	44.03

Enantiomerically enriched sample

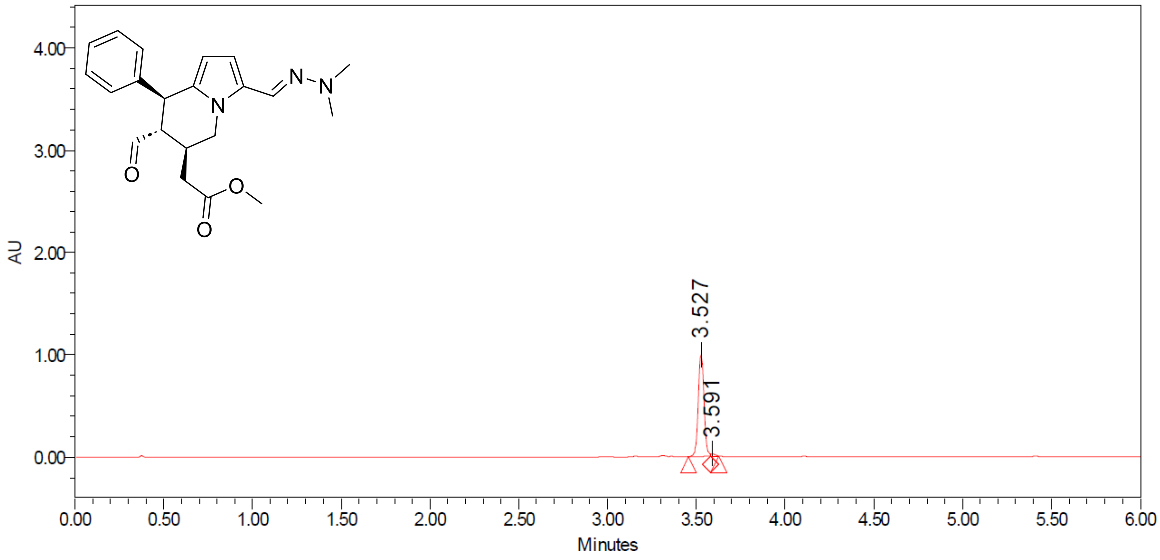

Peak Results

	RT	% Area
1	3.527	98.27
2	3.591	1.73

Methyl 2-((6*S*,7*S*,8*S*)-3-((*E*)-(2,2-dimethylhydrazono)methyl)-7-formyl-8-(4-methoxyphenyl)-5,6,7,8-tetrahydroindolizin-6-yl)acetate (**4b**)

Racemic sample

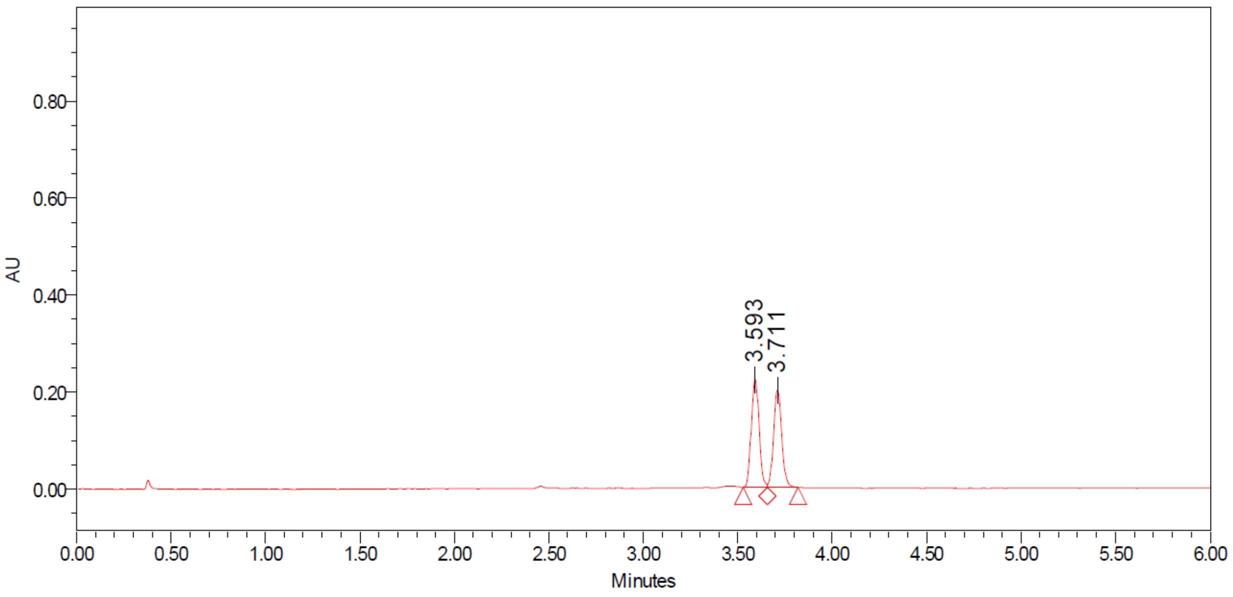

Peak Results

	RT	% Area
1	3.593	51.54
2	3.711	48.46

Enantiomerically enriched sample

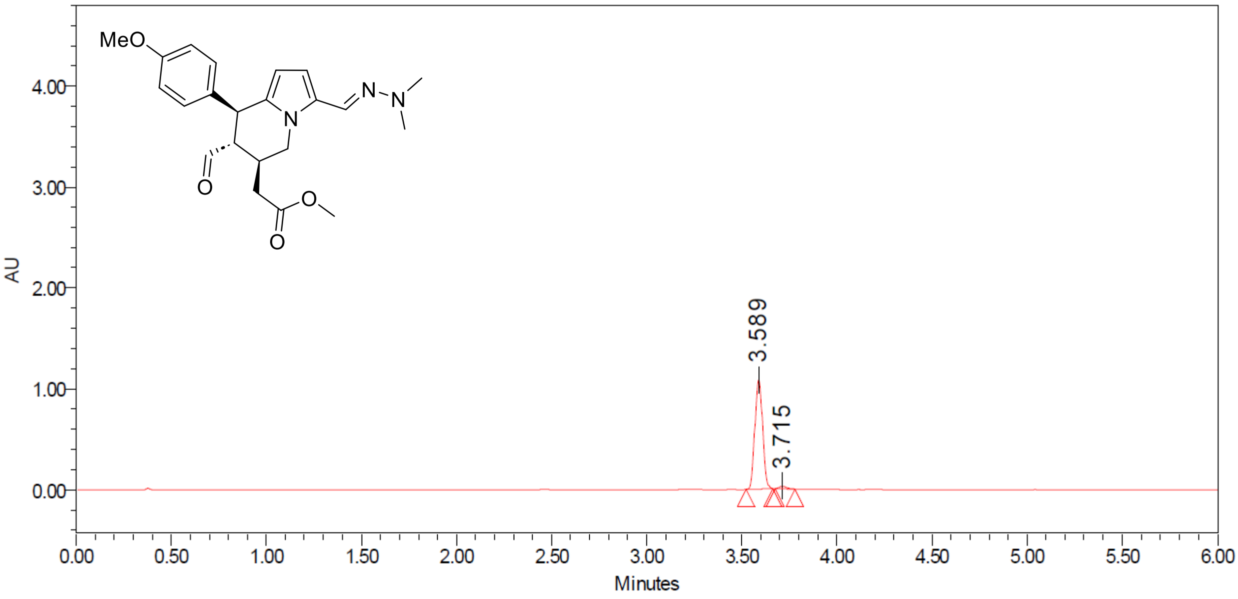

Peak Results

	RT	% Area
1	3.589	97.63
2	3.715	2.37

Methyl 2-((6*S*,7*S*,8*S*)-3-((*E*)-(2,2-dimethylhydrazono)methyl)-7-formyl-8-(3-methoxyphenyl)-5,6,7,8-tetrahydroindolizin-6-yl)acetate (**4c**)

Racemic sample

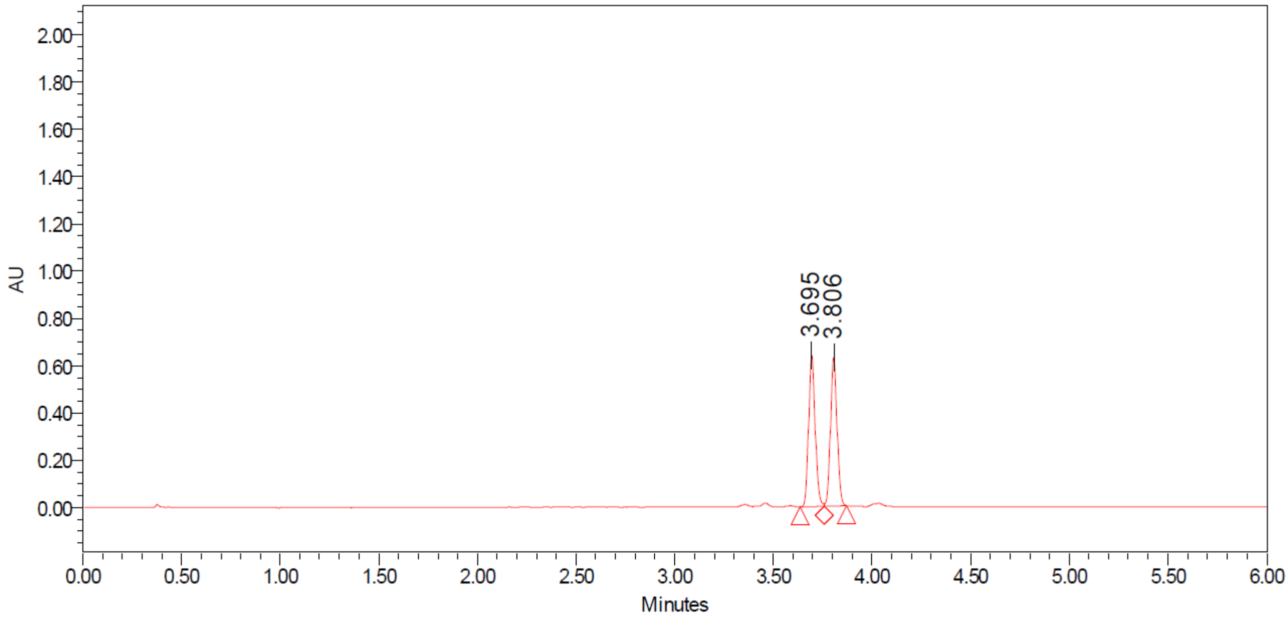

Peak Results

	RT	% Area
1	3.695	50.55
2	3.806	49.45

Enantiomerically enriched sample

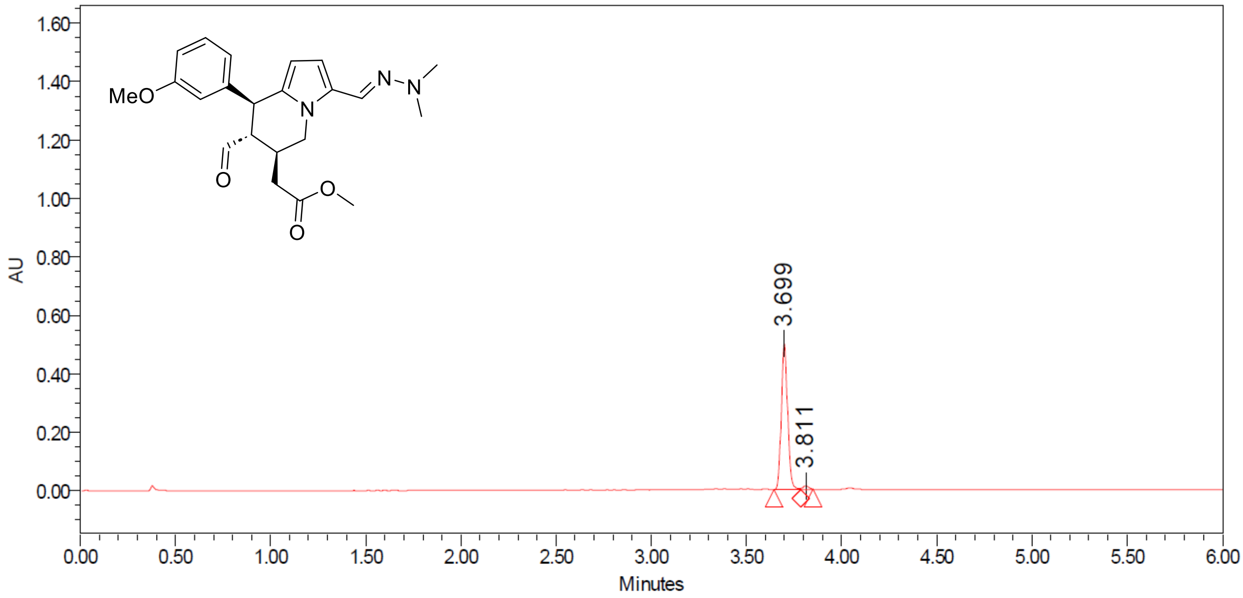

Peak Results

	RT	% Area
1	3.699	97.83
2	3.811	2.17

Methyl 2-((6*S*,7*S*,8*S*)-3-((*E*)-(2,2-dimethylhydrazono)methyl)-7-formyl-8-(2-methoxyphenyl)-5,6,7,8-tetrahydroindolizin-6-yl)acetate (**4d**)

Racemic sample

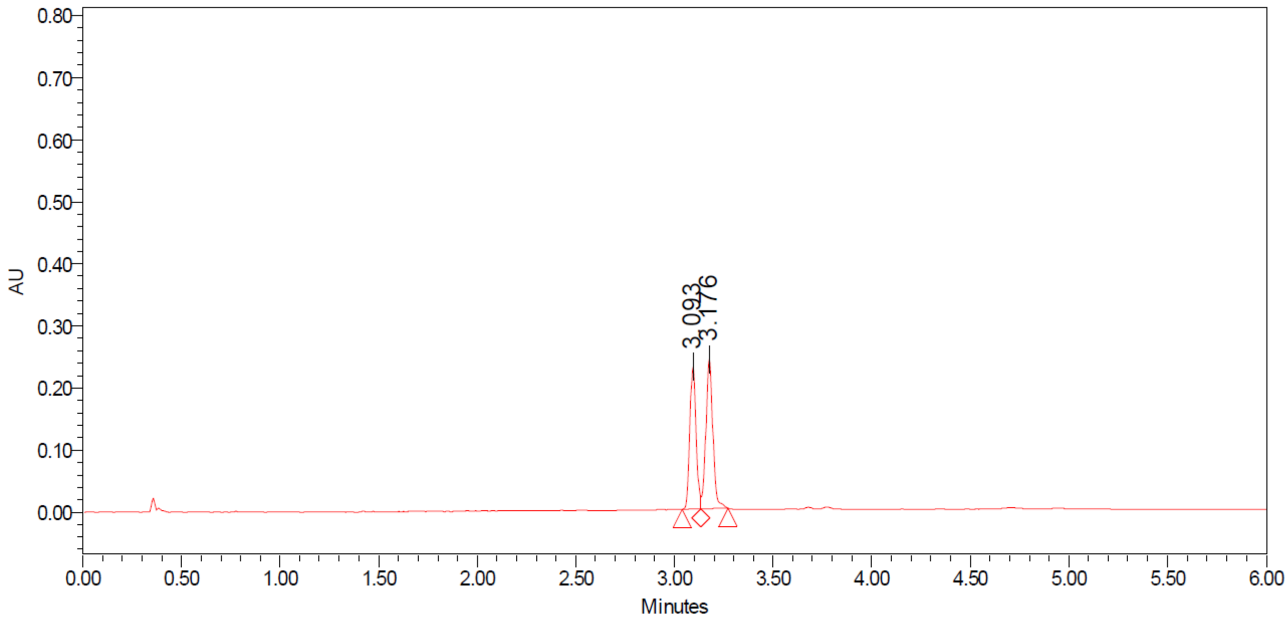

Peak Results

	RT	% Area
1	3.093	46.05
2	3.176	53.95

Enantiomerically enriched sample

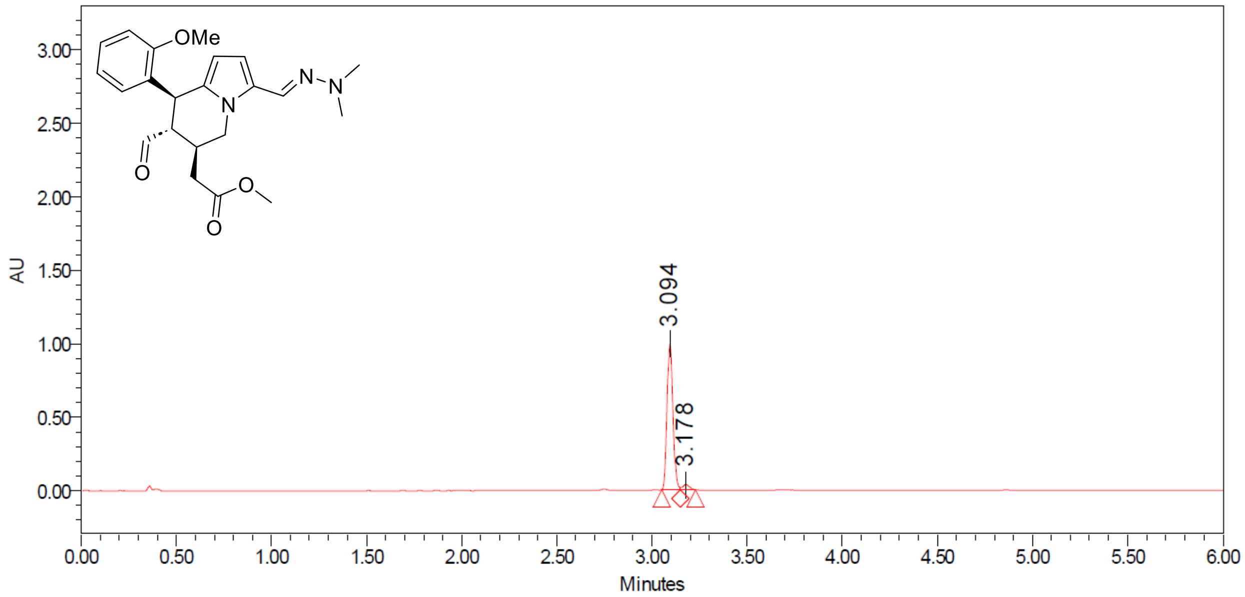

Peak Results

	RT	% Area
1	3.094	95.97
2	3.178	4.03

Methyl 2-((6*S*,7*S*,8*S*)-8-(4-cyanophenyl)-3-((*E*)-(2,2-dimethylhydrazono)methyl)-7-formyl-5,6,7,8-tetrahydroindolizin-6-yl)acetate (**4e**)

Racemic sample

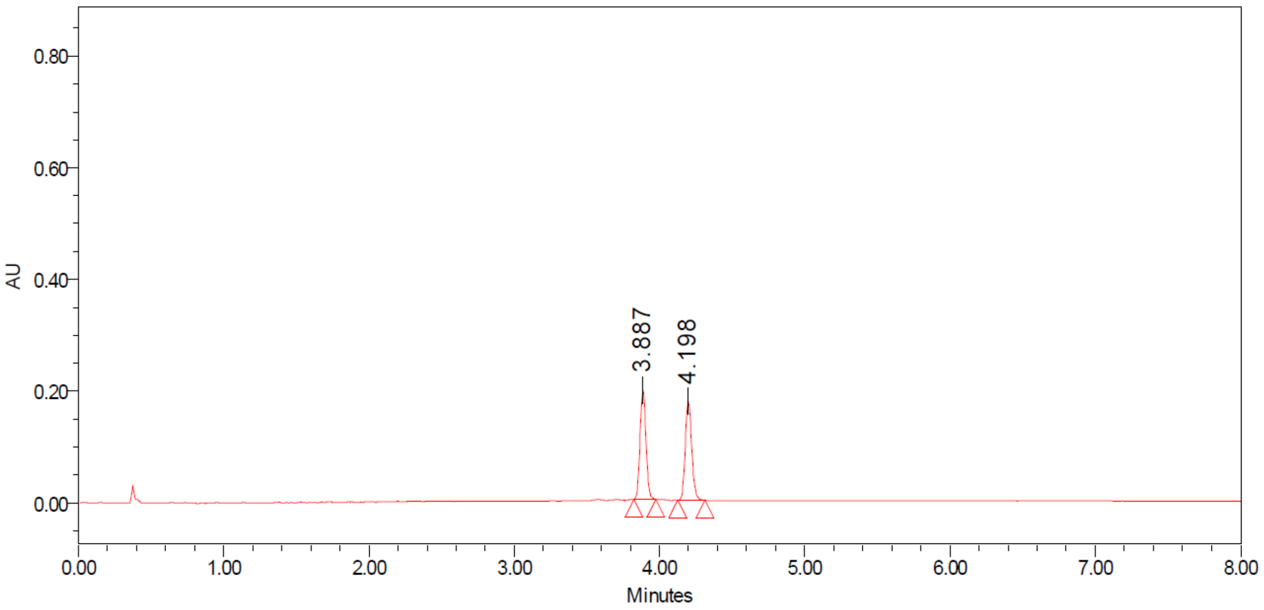

Peak Results

	RT	% Area
1	3.887	50.42
2	4.198	49.58

Enantiomerically enriched sample

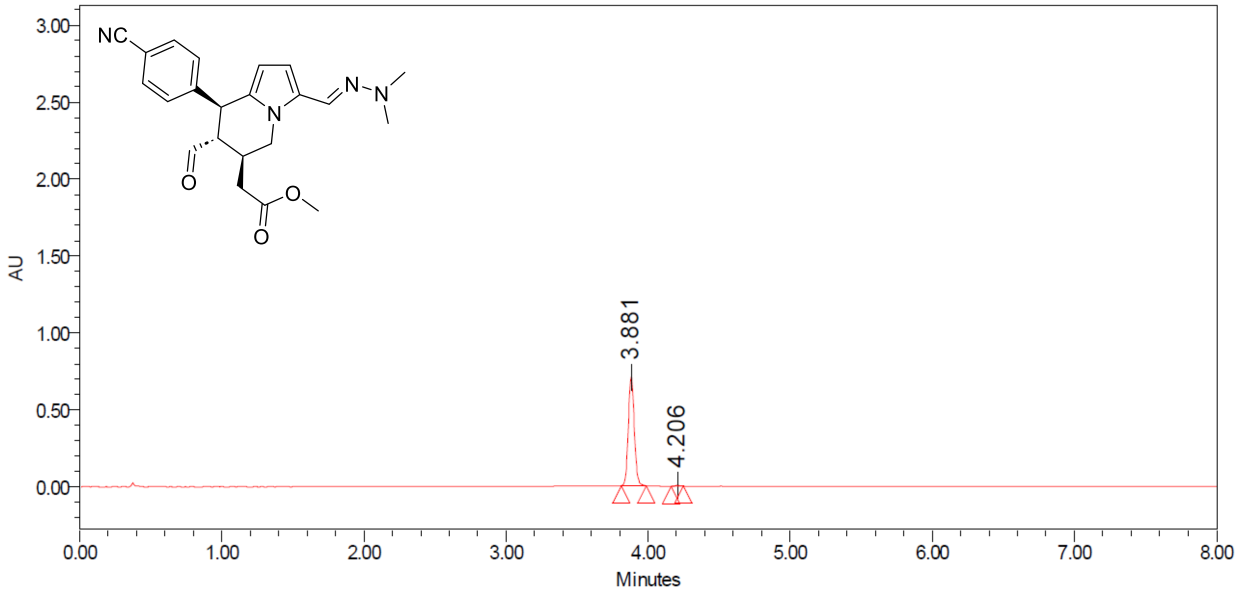

Peak Results

	RT	% Area
1	3.881	99.41
2	4.206	0.59

Methyl 2-((6*S*,7*S*,8*S*)-3-((*E*)-(2,2-dimethylhydrazono)methyl)-7-formyl-8-(4-(trifluoromethyl)phenyl)-5,6,7,8-tetrahydroindolizin-6-yl)acetate (**4f**)

Racemic sample

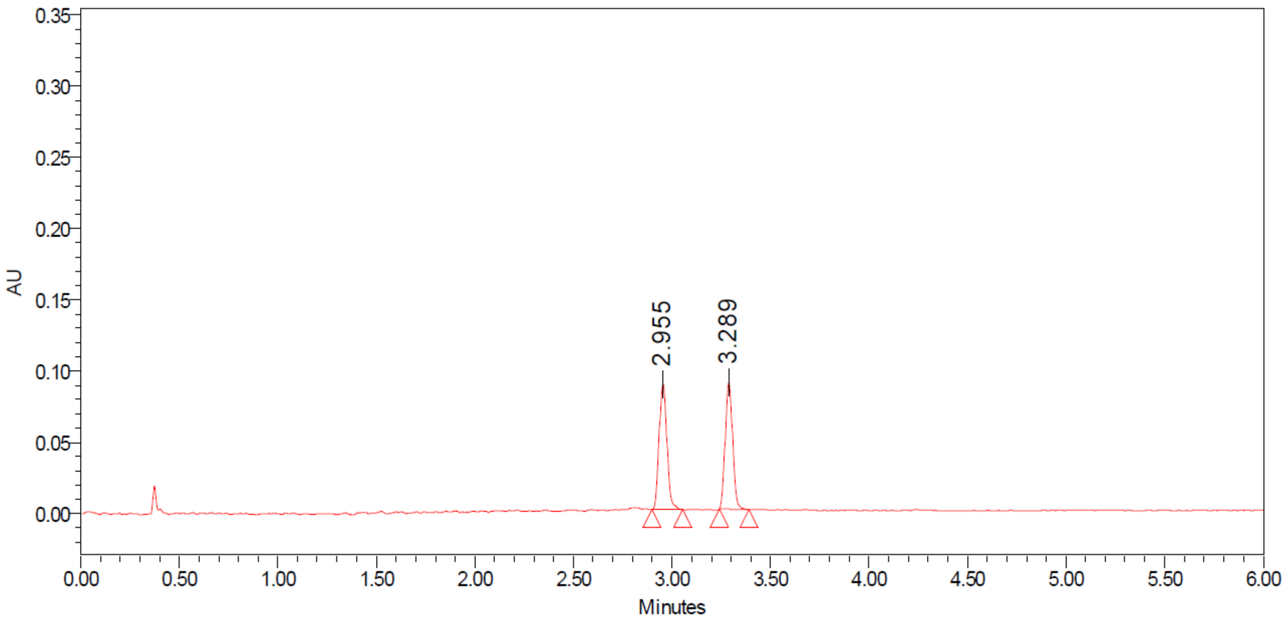

Peak Results

	RT	% Area
1	2.955	51.00
2	3.289	49.00

Enantiomerically enriched sample

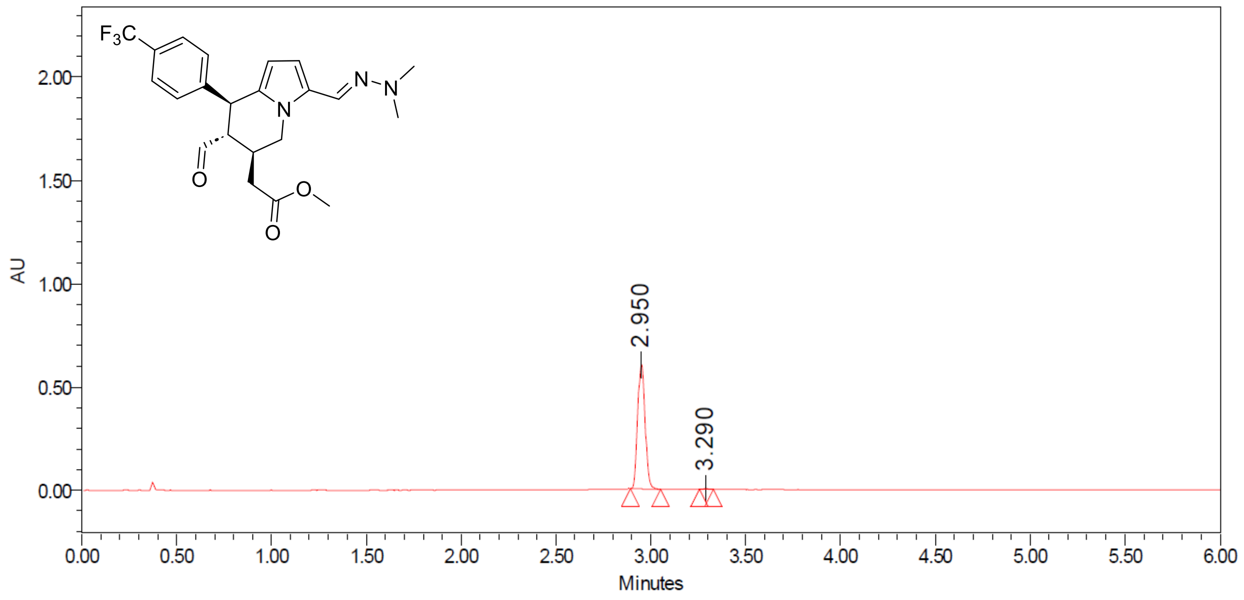

Peak Results

	RT	% Area
1	2.950	99.51
2	3.290	0.49

Methyl 2-((6*S*,7*S*,8*S*)-3-((*E*)-(2,2-dimethylhydrazono)methyl)-7-formyl-8-(4-nitrophenyl)-5,6,7,8-tetrahydroindolizin-6-yl)acetate (**4g**)

Racemic sample

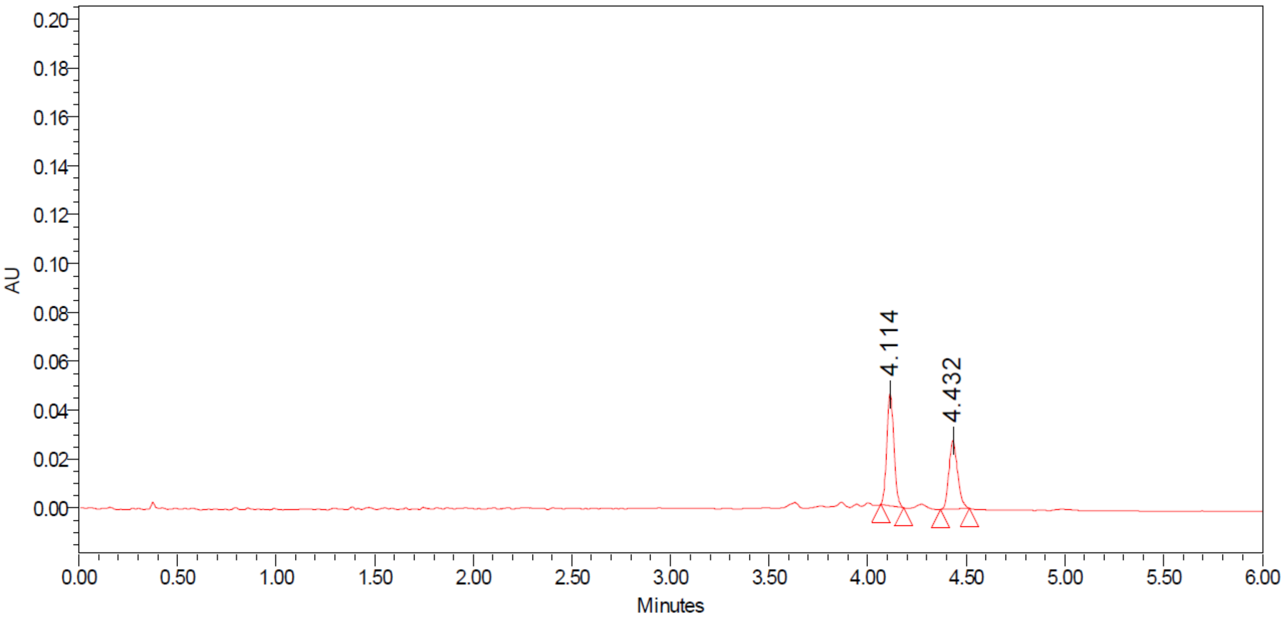

Peak Results

	RT	% Area
1	4.114	57.46
2	4.432	42.54

Enantiomerically enriched sample

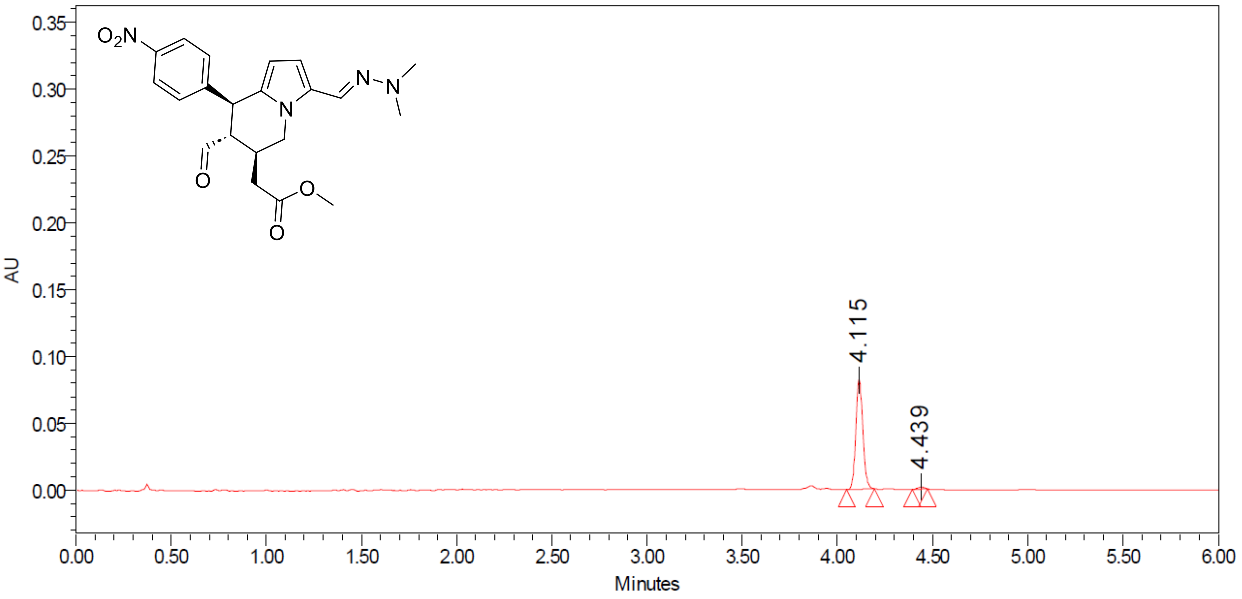

Peak Results

	RT	% Area
1	4.115	98.21
2	4.439	1.79

Methyl 2-((6*S*,7*S*,8*S*)-3-((*E*)-(2,2-dimethylhydrazono)methyl)-7-formyl-8-(*p*-tolyl)-5,6,7,8-tetrahydroindolizin-6-yl)acetate (**4h**)

Racemic sample

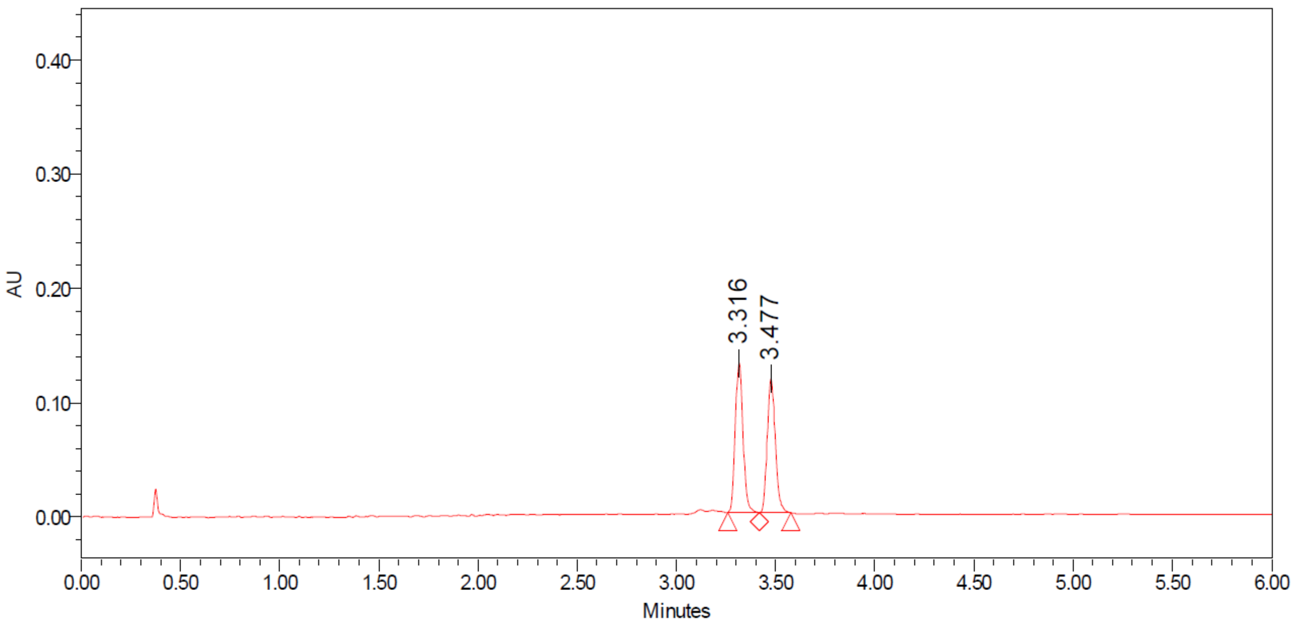

Peak Results

	RT	% Area
1	3.316	51.87
2	3.477	48.13

Enantiomerically enriched sample

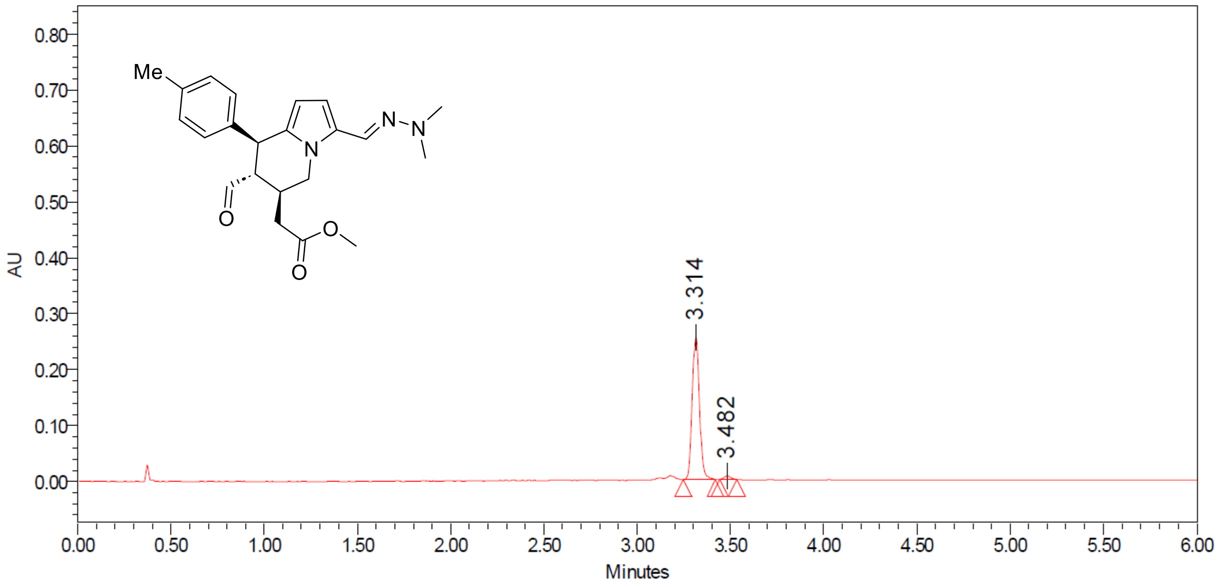

Peak Results

	RT	% Area
1	3.314	97.81
2	3.482	2.19

Methyl 2-((6*S*,7*S*,8*S*)-8-(4-chlorophenyl)-3-((*E*)-(2,2-dimethylhydrazono)methyl)-7-formyl-5,6,7,8-tetrahydroindolizin-6-yl)acetate (**4i**)

Racemic sample

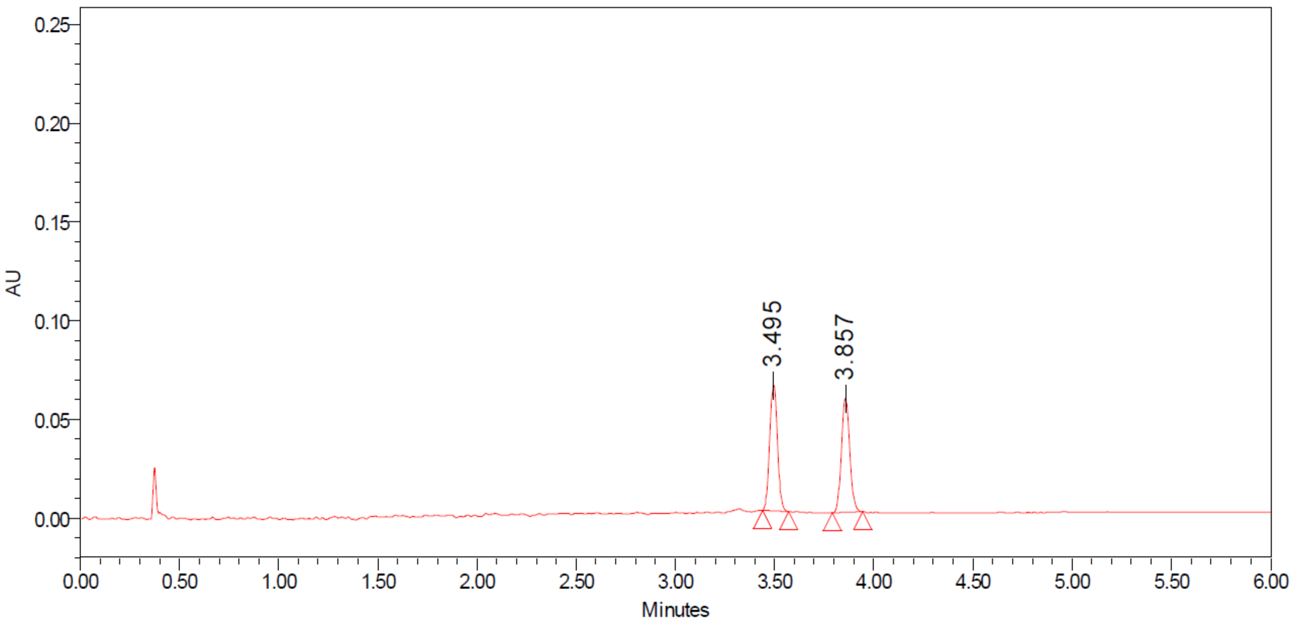

Peak Results

	RT	% Area
1	3.495	50.84
2	3.857	49.16

Enantiomerically enriched sample

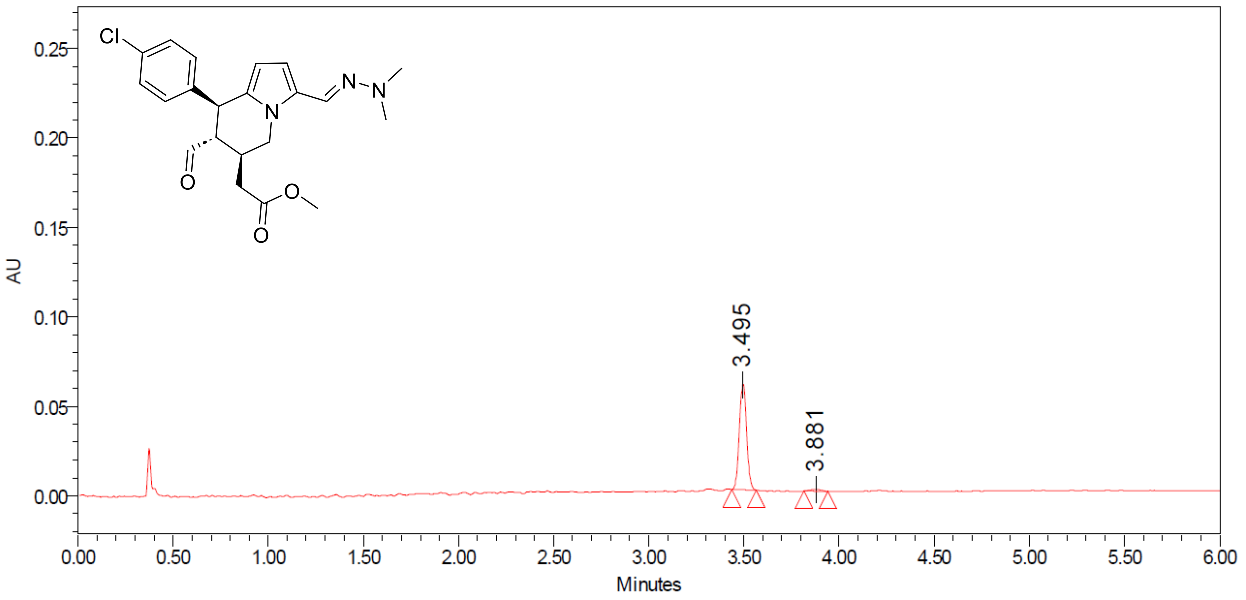

Peak Results

	RT	% Area
1	3.495	97.79
2	3.881	2.21

Methyl 2-((6*S*,7*S*,8*S*)-8-(2,4-dichlorophenyl)-3-((*E*)-(2,2-dimethylhydrazono)methyl)-7-formyl-5,6,7,8-tetrahydroindolizin-6-yl)acetate (**4j**)

Racemic sample

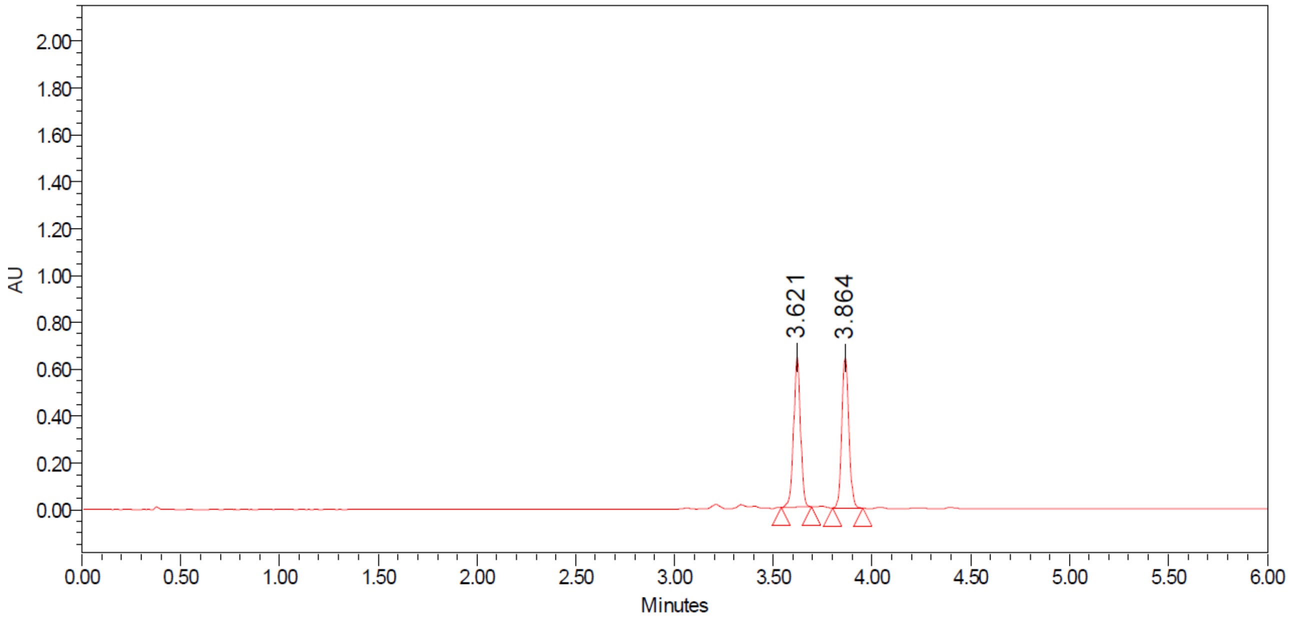

Peak Results

	RT	% Area
1	3.621	49.44
2	3.864	50.56

Enantiomerically enriched sample

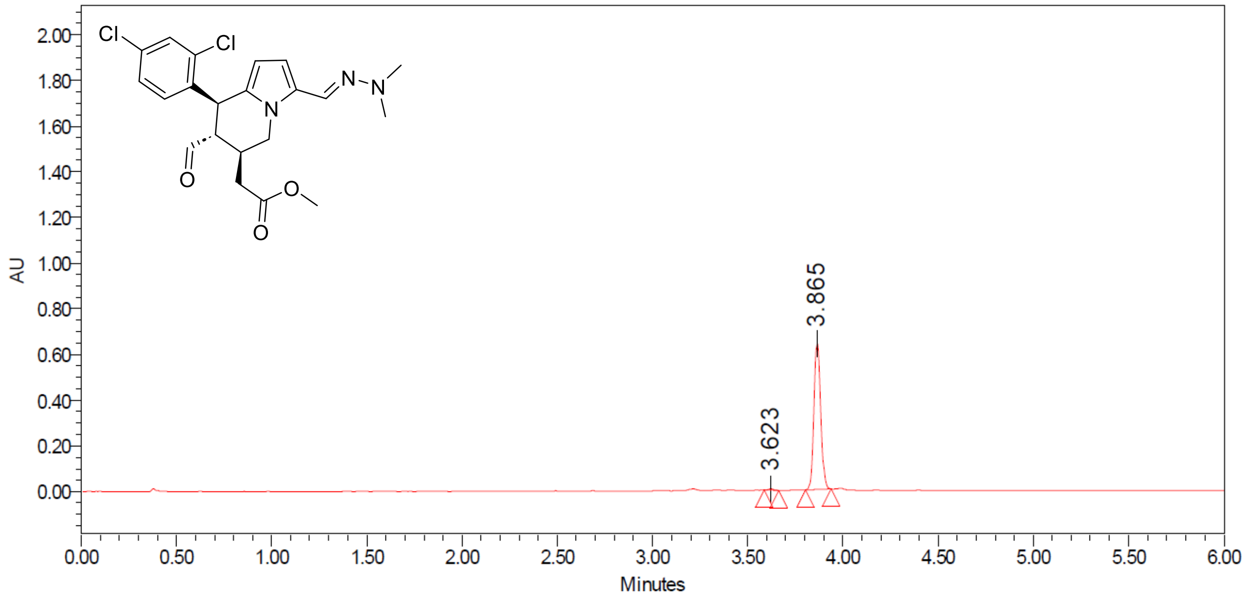

Peak Results

	RT	% Area
1	3.623	0.82
2	3.865	99.18

Methyl 2-((6*S*,7*S*,8*S*)-3-((*E*)-(2,2-dimethylhydrazono)methyl)-7-formyl-8-(naphthalen-1-yl)-5,6,7,8-tetrahydroindolizin-6-yl)acetate (**4k**)

Racemic sample

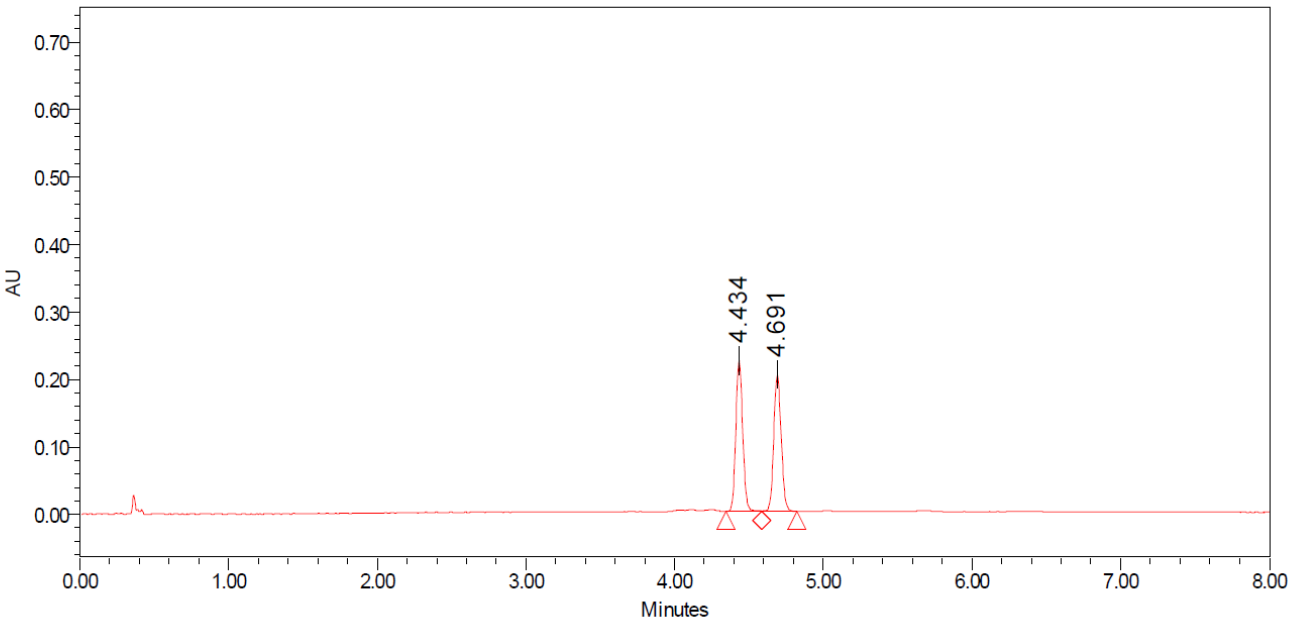

Peak Results

	RT	% Area
1	4.434	50.37
2	4.691	49.63

Enantiomerically enriched sample

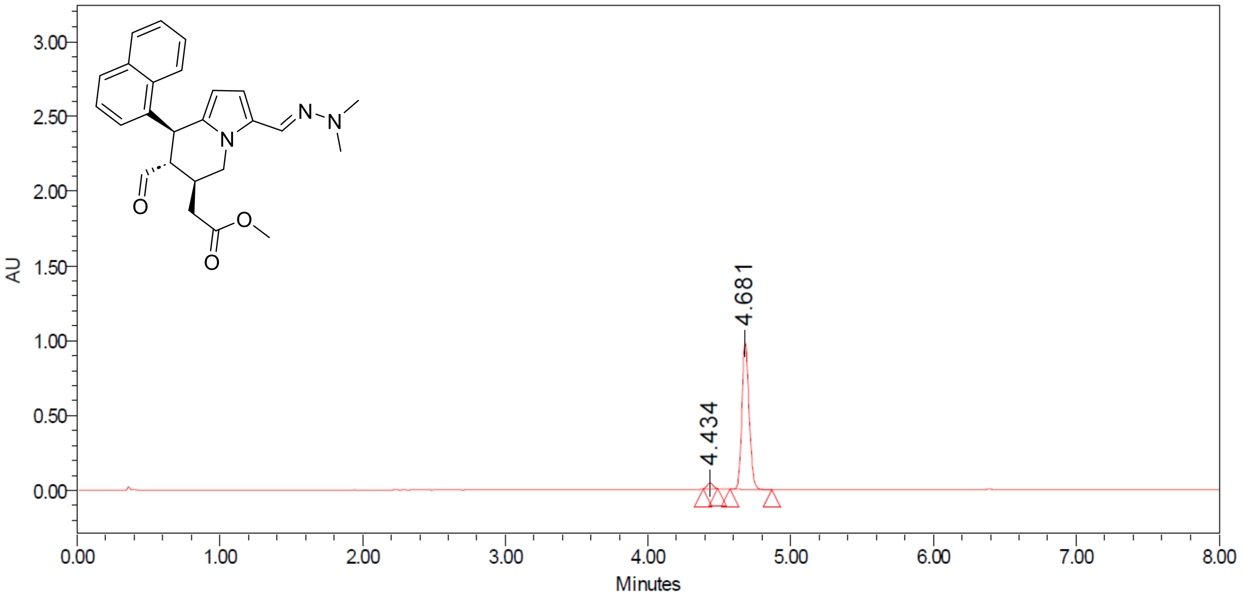

Peak Results

	RT	% Area
1	4.434	3.17
2	4.681	96.83

Methyl 2-((6*S*,7*S*,8*R*)-3-((*E*)-(2,2-dimethylhydrazono)methyl)-7-formyl-8-methyl-5,6,7,8-tetrahydroindolizin-6-yl)acetate (**4l**)

Racemic sample

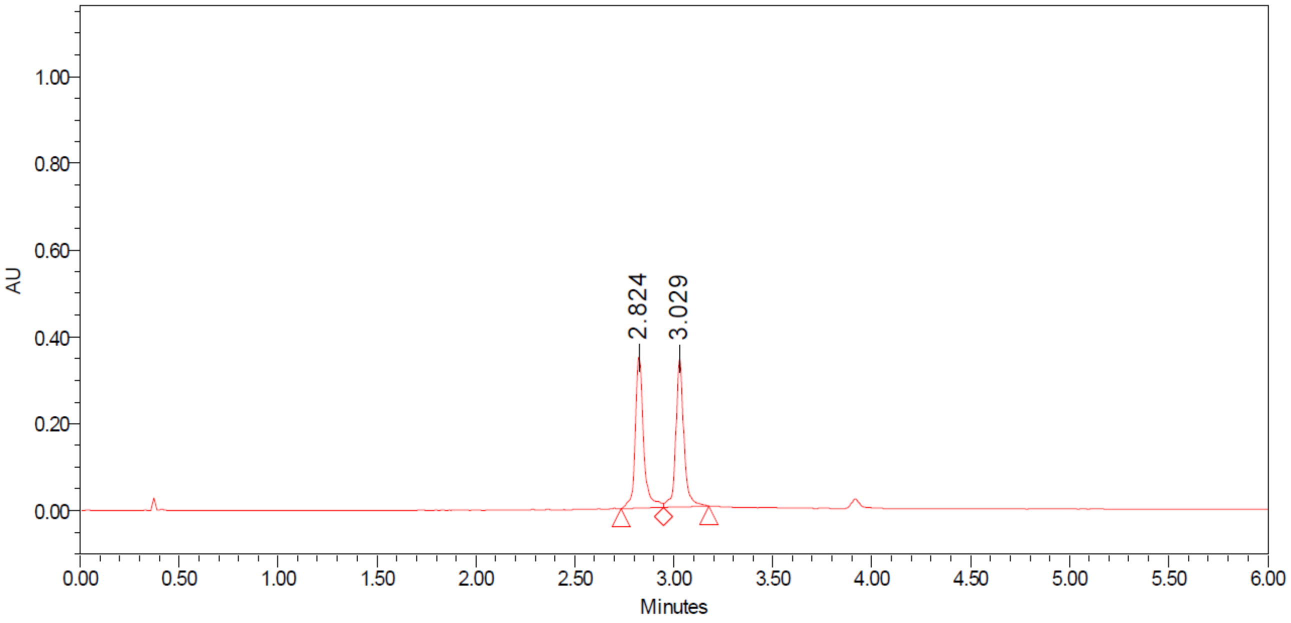

Peak Results

	RT	% Area
1	2.824	51.44
2	3.029	48.56

Enantiomerically enriched sample

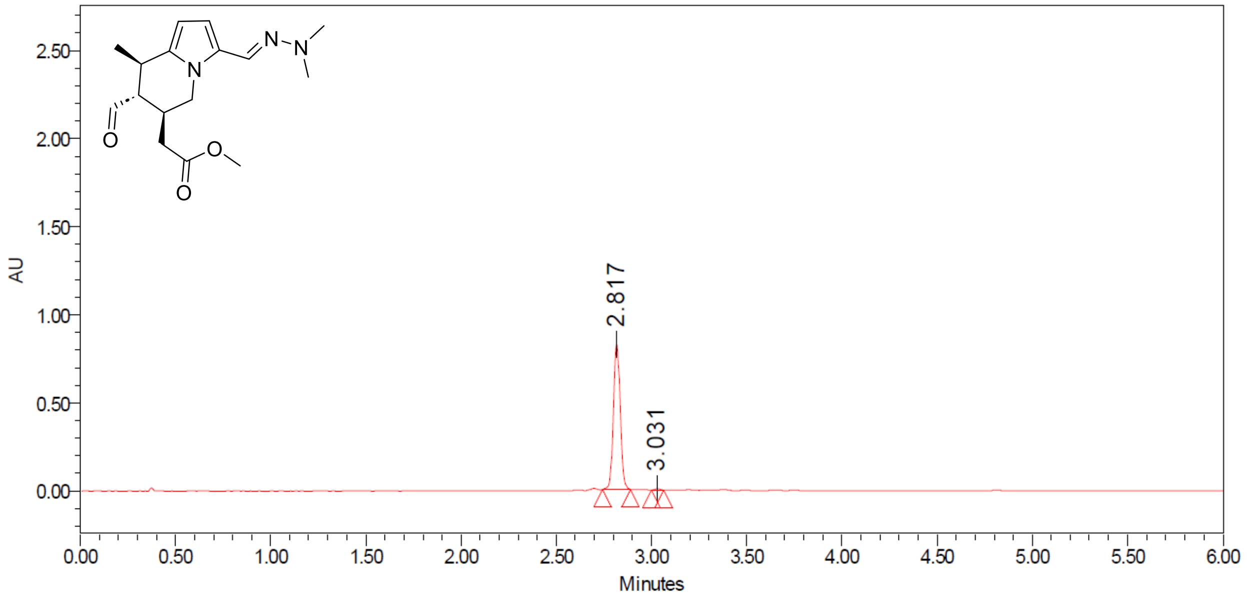

Peak Results

	RT	% Area
1	2.817	99.23
2	3.031	0.77

Methyl 2-((6*S*,7*S*,8*R*)-3-((*E*)-(2,2-dimethylhydrazono)methyl)-7-formyl-8-ethyl-5,6,7,8-tetrahydroindolizin-6-yl)acetate (**4m**)

Racemic sample

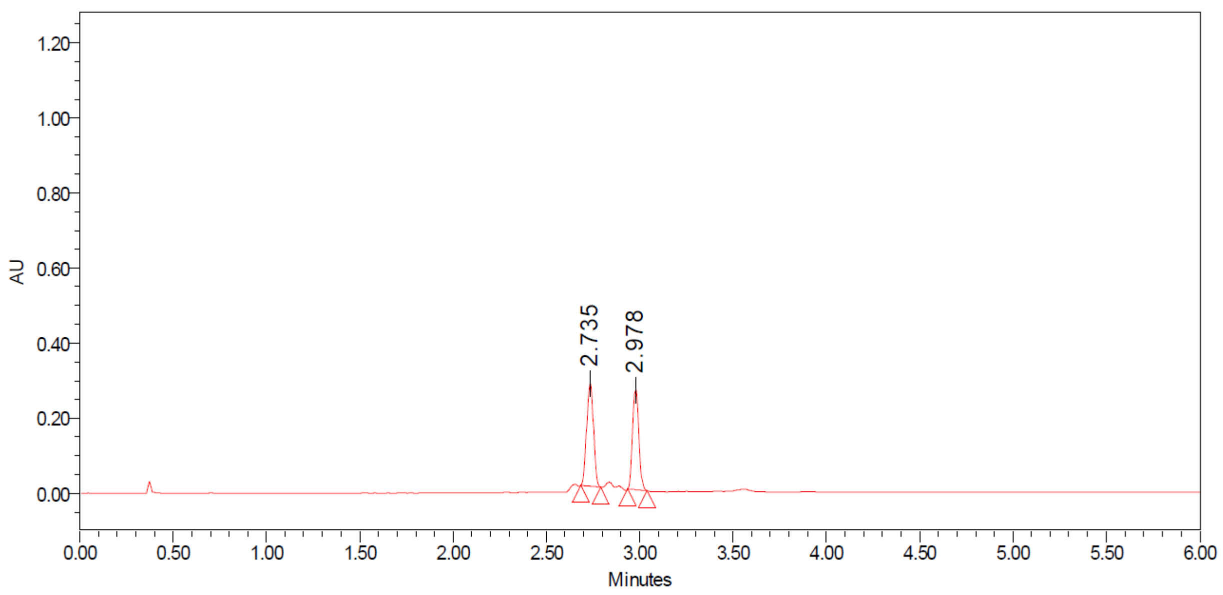

Peak Results

	RT	% Area
1	2.735	52.72
2	2.978	47.28

Enantiomerically enriched sample

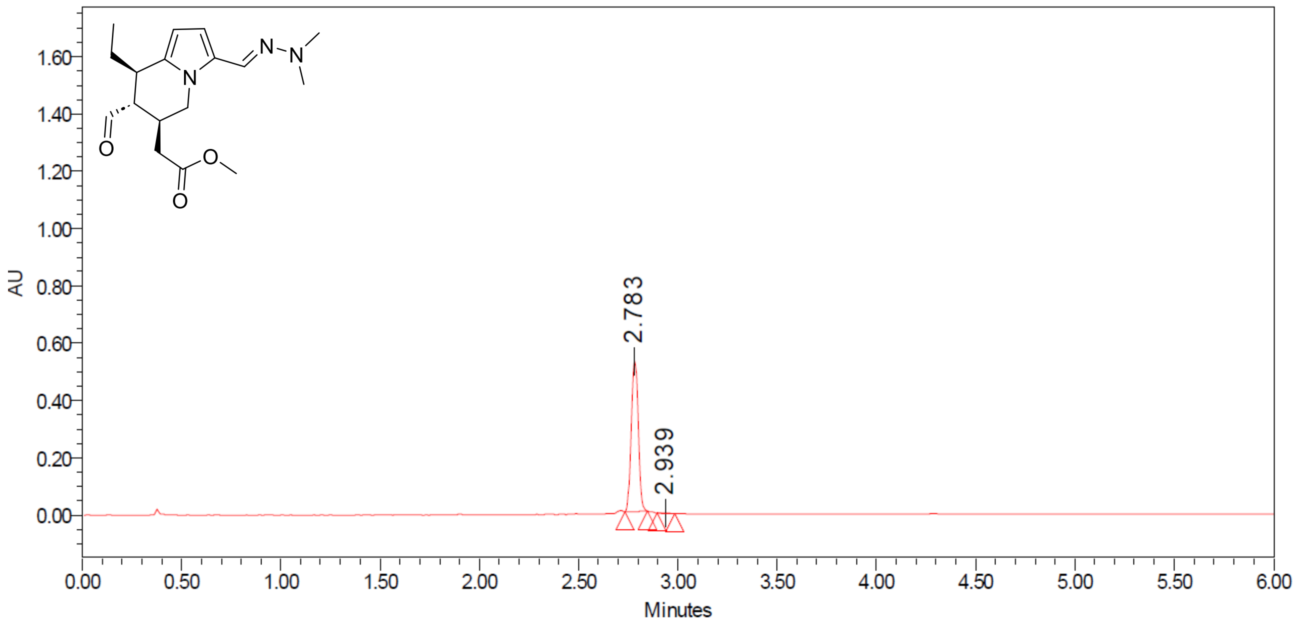

Peak Results

	RT	% Area
1	2.783	99.70
2	2.939	0.30

Methyl 2-((6*S*,7*S*,8*R*)-3-((*E*)-(2,2-dimethylhydrazono)methyl)-7-formyl-8-((*Z*)-hex-3-en-1-yl)-5,6,7,8-tetrahydroindolizin-6-yl)acetate (**4n**)

Racemic sample

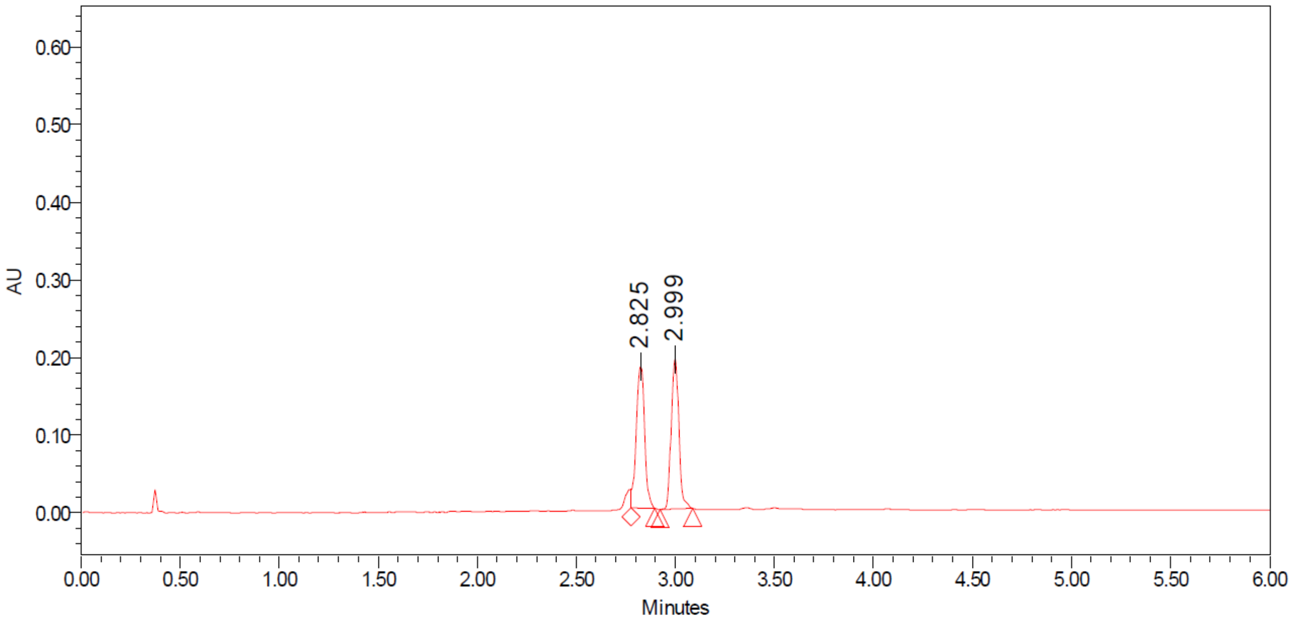

Peak Results

	RT	% Area
1	2.825	50.98
2	2.999	49.02

Enantiomerically enriched sample

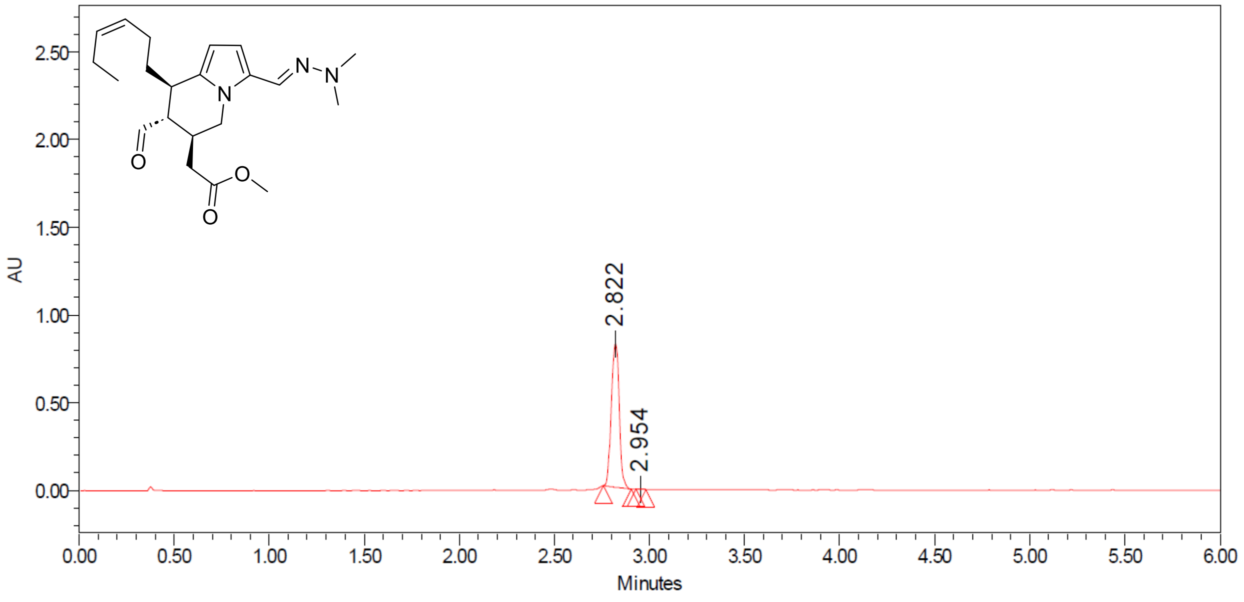

Peak Results

	RT	% Area
1	2.822	99.91
2	2.954	0.09

Methyl 2-((6*S*,7*S*,8*R*)-3-((*E*)-(2,2-dimethylhydrazono)methyl)-7-formyl-8-(phenethoxymethyl)-5,6,7,8-tetrahydroindolizin-6-yl)acetate (**4o**)

Racemic sample

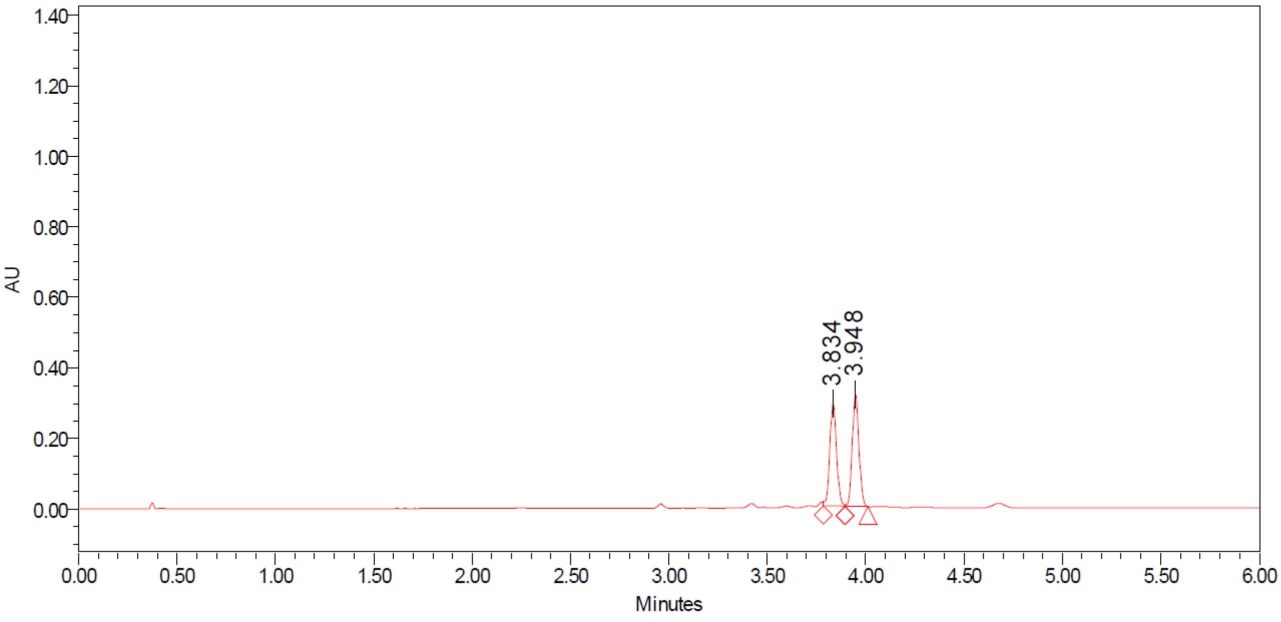

Peak Results

	RT	% Area
1	3.834	48.12
2	3.948	51.88

Enantiomerically enriched sample

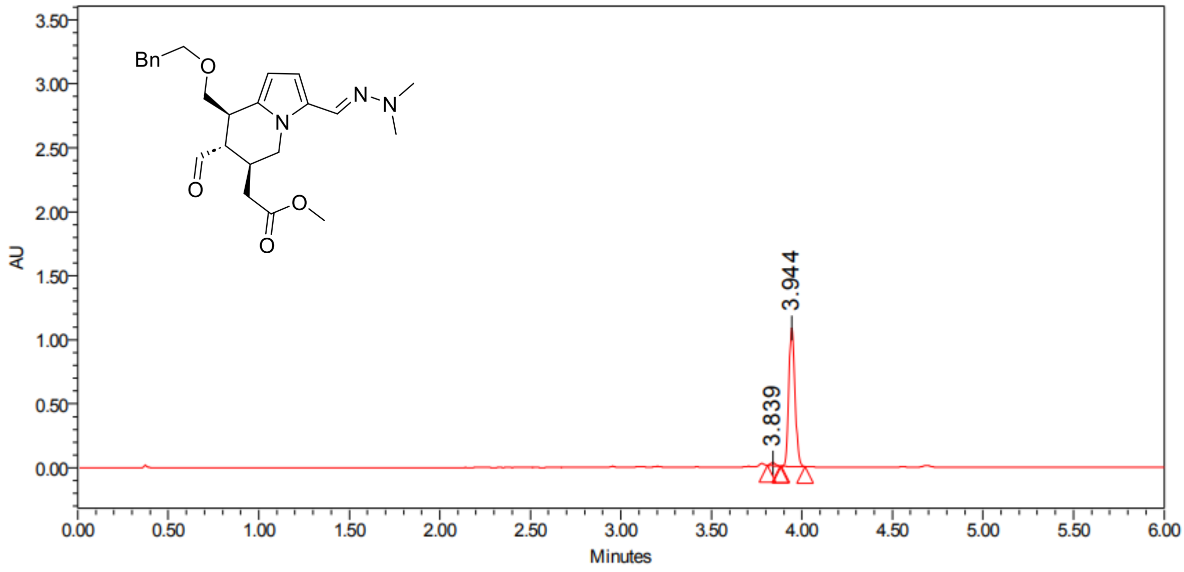

Peak Results

	RT	% Area
1	3.839	1.90
2	3.944	98.10
